# Targeting PRMT9-mediated arginine methylation suppresses cancer stem cell maintenance and elicits cGAS-mediated anticancer immunity

**DOI:** 10.1038/s43018-024-00736-x

**Published:** 2024-02-27

**Authors:** Haojie Dong, Xin He, Lei Zhang, Wei Chen, Yi-Chun Lin, Song-Bai Liu, Huafeng Wang, Le Xuan Truong Nguyen, Min Li, Yinghui Zhu, Dandan Zhao, Lucy Ghoda, Jonathan Serody, Benjamin Vincent, Leo Luznik, Ivana Gojo, Joshua Zeidner, Rui Su, Jianjun Chen, Ritin Sharma, Patrick Pirrotte, Xiwei Wu, Weidong Hu, Weidong Han, Binghui Shen, Ya-Huei Kuo, Jie Jin, Amandeep Salhotra, Jeffrey Wang, Guido Marcucci, Yun Lyna Luo, Ling Li

**Affiliations:** 1 Department of Hematological Malignancies Translational Science, Gehr Family Center for Leukemia Research, Hematologic Malignancies and Stem Cell Transplantation Institute, Beckman Research Institute, City of Hope Medical Center, Duarte, CA USA; 2 Integrative Genomics Core, Beckman Research Institute, City of Hope Medical Center, Duarte, CA USA; 3 Department of Pharmaceutical Sciences, College of Pharmacy, Western University of Health Sciences, Pomona, CA USA; 4 Suzhou Key Laboratory of Medical Biotechnology, Suzhou Vocational Health College, Suzhou, People’s Republic of China; 5 Department of Hematology, the First Affiliated Hospital, Zhejiang University School of Medicine, Hangzhou, People’s Republic of China; 6 Division of Biostatistics, Department of Computational and Quantitative Medicine, Beckman Research Institute, City of Hope Medical Center, Duarte, CA USA; 7 Department of Medicine, Division of Hematology, Lineberger Comprehensive Cancer Center, University of North Carolina, Chapel Hill, NC USA; 8 Department of Microbiology and Immunology and Department of Pathology and Laboratory Medicine, University of North Carolina School of Medicine, Chapel Hill, NC USA; 9 Department of Microbiology and Immunology, Computational Medicine Program, Lineberger Comprehensive Cancer Center, University of North Carolina, Chapel Hill, NC USA; 10 Department of Oncology and Sidney Kimmel Comprehensive Cancer Center, The Johns Hopkins University School of Medicine, Baltimore, MD USA; 11 Department of Systems Biology, Beckman Research Institute, City of Hope Medical Center, Duarte, CA USA; 12 Cancer & Cell Biology Division, The Translational Genomics Research Institute, Phoenix, AZ USA; 13 Integrated Mass Spectrometry Shared Resource, City of Hope Medical Center, Duarte, CA USA; 14 Department of Computational and Quantitative Medicine, Beckman Research Institute, City of Hope Medical Center, Duarte, CA USA; 15 Department of Immunology and Theranostics, Beckman Research Institute, City of Hope Medical Center, Duarte, CA USA; 16 Sir Run Run Shaw Hospital, Zhejiang University, Hangzhou, People’s Republic of China; 17 Department of Cancer Genetics and Epigenetics, Beckman Research Institute, City of Hope Medical Center, Duarte, CA USA; 18 Department of Hematology and HCT, City of Hope Medical Center, Duarte, CA USA; 19 Department of Pediatrics, Beckman Research Institute, City of Hope Medical Center, Duarte, CA USA

**Keywords:** Cancer, Tumour immunology

## Abstract

Current anticancer therapies cannot eliminate all cancer cells, which hijack normal arginine methylation as a means to promote their maintenance via unknown mechanisms. Here we show that targeting protein arginine *N*-methyltransferase 9 (PRMT9), whose activities are elevated in blasts and leukemia stem cells (LSCs) from patients with acute myeloid leukemia (AML), eliminates disease via cancer-intrinsic mechanisms and cancer-extrinsic type I interferon (IFN)-associated immunity. PRMT9 ablation in AML cells decreased the arginine methylation of regulators of RNA translation and the DNA damage response, suppressing cell survival. Notably, PRMT9 inhibition promoted DNA damage and activated cyclic GMP-AMP synthase, which underlies the type I IFN response. Genetically activating cyclic GMP-AMP synthase in AML cells blocked leukemogenesis. We also report synergy of a PRMT9 inhibitor with anti-programmed cell death protein 1 in eradicating AML. Overall, we conclude that PRMT9 functions in survival and immune evasion of both LSCs and non-LSCs; targeting PRMT9 may represent a potential anticancer strategy.

## Abstract (web summary)

Dong et al. demonstrate that targeting arginine *N*-methyltransferase 9 (PRMT9) inhibits cancer stem cells in the context of acute myeloid leukemia via type I interferon-associated immunity. Furthermore, they show that PRMT9 inhibition synergizes with anti-programmed cell death protein 1.

## Main

The outcomes for patients with acute myeloid leukemia (AML) remain poor^1^. Allogeneic hematopoietic stem cell transplantation has emerged as the only cure. However, its applicability is restricted^2^. Success of immunotherapies has driven interest in developing effective antileukemia drugs, including immune checkpoint inhibitors (ICIs)^3–5^. However, translation of existing T cell-leveraging strategies to AML treatment remains challenging.

The cyclic GMP-AMP synthase (cGAS)–stimulator of interferon genes (STING) signaling triggers the type I interferon (IFN) response^6^ and can prime T cell function. Specifically, preexisting functional T cells are required for responses to ICI treatment^6^. cGAS activity is stimulated by cytosolic DNA and generates cyclic GMP-AMP (cGAMP)^7,8^. Notably, cGAMP exported from tumor cells serves as an ‘immunotransmitter’ to activate the STING receptor on dendritic cells (DCs), which then activates the production of type I IFN^8^. Moreover, administration of the STING agonist blocked AML development in in vivo models^7^. However, STING agonists show only modest clinical benefits^9^. To address this challenge, we are developing a strategy based on stimulating cancer-endogenous cGAS.

Protein arginine methylation is a posttranslational modification functioning in cellular processes^10^. Protein arginine methyltransferases (PRMTs) have emerged as druggable targets. Accordingly, inhibitors of two major PRMTs to treat malignancies are under clinical trials. However, one concern is that PRMT1 and PRMT5 are responsible for most arginine methylation of essential histone markers^10^. Therefore, the relevance of other individual PRMTs to different cancers should be considered. PRMT9 is the less-known PRMT. In this study, we performed analyses that supported PRMT9 as a potential target and developed an inhibitory compound as a tool to probe PRMT9 activity.

## Results

### PRMT9 levels are elevated in leukemia stem cells

**Fig. 1:**
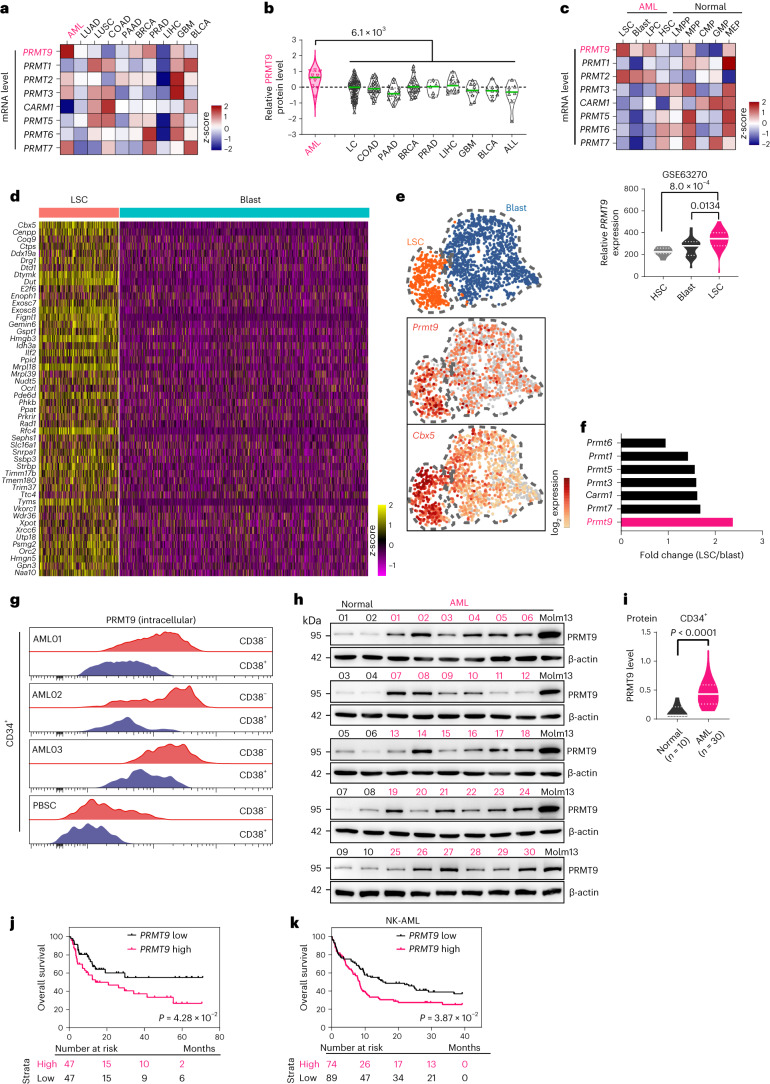
PRMT9 levels are elevated in AML. **a**, PRMTs mRNA levels in the most deadly cancer types from the TCGA PanCancer Atlas. AML (*n* = 173), lung adenocarcinoma (LUAD) (*n* = 510), lung squamous cell carcinoma (LUSC) (*n* = 484), colon adenocarcinoma (COAD) (*n* = 438), pancreatic adenocarcinoma (PAAD) (*n* = 177), breast cancer (BRCA) (*n* = 1,082), prostate adenocarcinoma (PRAD) (*n* = 493), liver hepatocellular carcinoma (LIHC) (*n* = 366), glioblastoma (GBM) (*n* = 160) and bladder carcinoma (BLCA) (*n* = 407). *Z*-scores were determined based on the average expression of each PRMT. PRMT8 was undetectable (*n* represents the number of tissue samples; https://www.cbioportal.org/). **b**, PRMT9 protein levels in AML relative to other cancer lines. Data were from the DepMap portal (https://DepMap.org/portal/). AML (*n* = 10), lung cancer (*n* = 75), COAD (*n* = 29), PAAD (*n* = 17), BRCA (*n* = 29), PRAD (*n* = 5), LIHC (*n* = 12), GBM (*n* = 11), BLCA (*n* = 9) and acute lymphoblastic leukemia (ALL) (*n* = 8). The *P* value was determined using an unpaired two-sided *t*-test (*n* represents the number of different cancer cell lines. **c**, Top: PRMTs mRNA levels in normal hematopoietic subsets from healthy donors (*n* = 7) or leukemia subsets from patients with AML (*n* = 21) in GSE63270. *Z*-scores were determined based on the average expression of each PRMT. Bottom: the violin plots show *PRMT9* expression in LSCs versus normal HSCs and in LSCs versus leukemia blasts. The LSC versus HSC *P* value was determined using an unpaired two-sided *t*-test. The LSC versus blast *P* value was determined using a paired two-sided *t*-test (*n* represents the number of patients). CMP, common myeloid progenitor; GMP, granulocyte-monocyte progenitor; LMPP, lympho-myeloid primed progenitor; MEP, megakaryocytic-erythroid progenitor; MPP, multipotent progenitor. **d**, Fifty representative genes from the MA9 mouse LSC signature. scRNA-seq of MA9 mouse bone marrow (same dataset in Fig. 5h). **e**, LSC and blast clusters from **d**. Shown are representative LSC gene (*Cbx5*) and *Prmt9* levels. **f**, Average fold change in all *Prmt* levels in an LSC versus a blast cluster based on **e**. **g**, Intracellular staining of PRMT9 in CD34^+^CD38^−^ or CD34^+^CD38^+^ populations in PBSCs from individuals with AML (*n* = 3 individuals) or normal PBSCs. **h**, PRMT9 protein levels in AML CD34^+^ (*n* = 30) versus normal PBSC (*n* = 10) counterparts. **i**, Quantitative summary of **h**. The *P* value was determined using an unpaired two-sided *t*-test (*n* represents the number of patients or healthy donors). **j**, Kaplan–Meier survival analysis of the in-house AML cohort (Supplementary Table 2; *n* = 94) after dichotomization for median *PRMT9* mRNA levels. **k**, Kaplan–Meier survival analysis of another cohort (GSE12417) after dichotomization for *PRMT9* levels below (black, *n* = 74) or above (red, *n* = 89) 9.62 log_2_-transformed intensity. The threshold was discovered by classifying patients into two clusters using the partitioning around medoids algorithm. *P* values were determined using a log-rank (Mantel–Cox) test (*n* represents the number of patients). Source data

**Extended Data Fig. 1:**
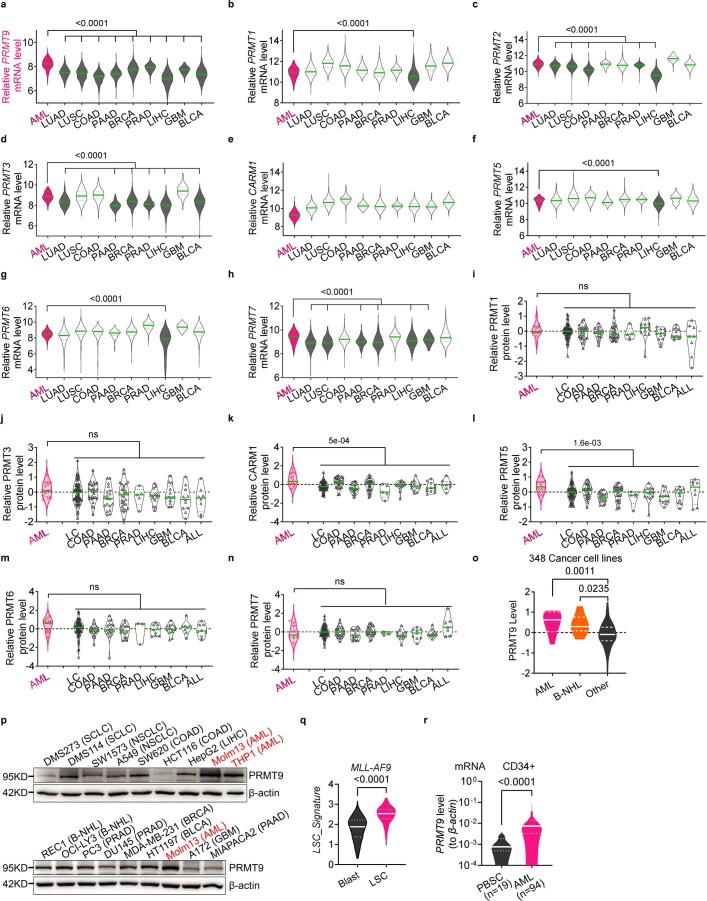
PRMT9 levels are elevated in AML. **a–h**, PRMTs mRNA levels in cancers from TCGA PanCancer Atlas. AML (n = 173), Lung adenocarcinoma (LUAD, n = 510), Lung squamous cell carcinoma (LUSC, n = 484), Colon Adenocarcinoma (COAD, n = 438), Pancreatic adenocarcinoma (PAAD, n = 177), Breast Cancer (BRCA, n = 1082), Prostate adenocarcinoma (PRAD, n = 493), Liver Hepatocellular Carcinoma (LIHC, n = 366), Glioblastoma (GBM, n = 160), Bladder Carcinoma (BLCA, n = 407). PRMT expression in AML (red) was compared with other cancers (only with significant difference were indicated). The violin plot in gray indicates PRMTs (except PRMT8 which is undetectable) level was significantly higher in AML than indicated cancer type. P values (a-h) were determined by unpaired two-sided t-tests. ‘n’ represents the number of patients. **i–n**, PRMTs protein levels in AML lines relative to those seen in lines representing the other deadly cancers. AML (n = 14), Lung Cancer (LC, n = 77), COAD (n = 29), PAAD (n = 17), BRCA (n = 29), PRAD (n = 5), LIHC (n = 12), GBM (n = 11), BLCA (n = 9) and Acute Lymphoblastic Leukemia (ALL, n = 8). Data was based on DepMap. P values were determined by unpaired two-sided t test. ‘n’ represents number of cancer cell lines. **o**, PRMT9 levels in AML (n = 10) and B-NHL (n = 8) lines relative to lines from other deadly cancers (n = 330), based on DepMap. P values were determined by one-way ANOVA. ‘n’ represents number of cell lines. **p**, PRMT9 protein levels in lines from most-deadly cancers, (n = 1). **q**, Expression level of LSC-signature-genes in LSC (n = 383 cells) and Blast (n = 1191 cells) based on MA9 scRNAseq analyses. P value was determined by unpaired two-sided t test. **r**, Q-PCR analysis of PRMT9 levels in AML CD34+ cells from an in-house cohort (n = 94 patients) and from PBSCs from healthy donors (n = 19). PRMT9 levels were normalized to β-actin. P value was determined by unpaired two-sided t test. Source data

**Extended Data Fig. 2:**
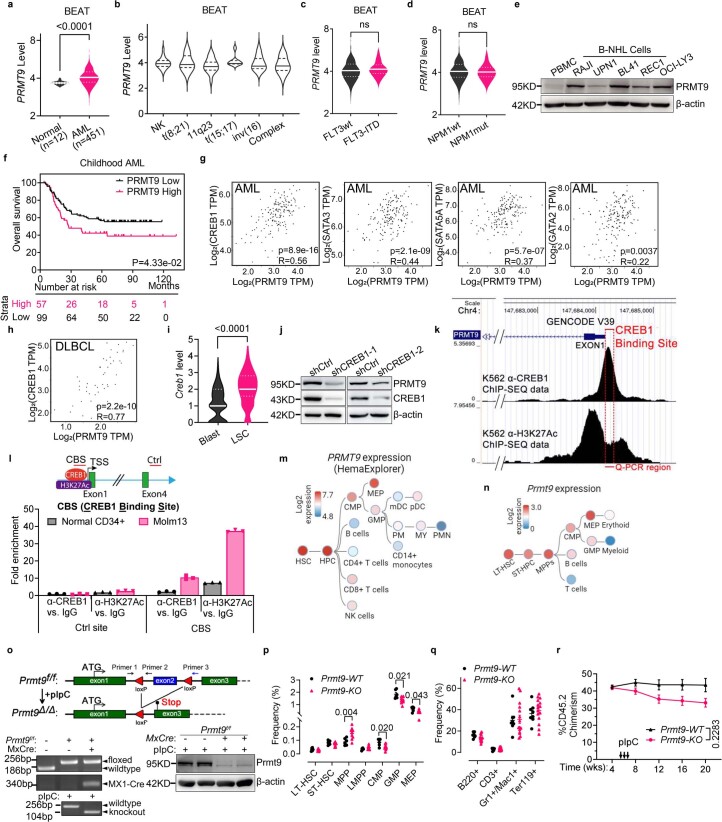
PRMT9 function is dispensable for normal hematopoiesis. **a**, *PRMT9* mRNA levels from BEAT AML dataset of mononuclear cells (MNCs) from AML cases (n = 451 patients) and healthy donors (n = 12). P value was determined by unpaired two-sided t test. **b**, *PRMT9* levels in AML cases with different cytogenetic karyotypes from BEAT AML. NK: n = 107, t (8;21): n = 11, 11q23: n = 15, t (15;17): n = 15, inv (16): n = 25, Complex: n = 32. ‘n’ represents the number of patients. **c, d**, *PRMT9* levels in BEAT AML of FLT3-ITD (c) and NPM1 (d) mutated subsets relative to their WT counterparts. FLT3wt n = 346, FLT3-ITD: n = 105; NPM1wt: n = 340, NPM1mut: n = 108. P value was determined by unpaired two-sided Mann–Whitneyt test. ‘n’ represents the number of patients. **e**, PRMT9 protein levels in B-NHL lines relative to PBMC control, (n = 1). **f**, Kaplan-Meier survival analysis of a cohort (TARGET-AML) after dichotomization for *PRMT9* levels below (green, n = 99) or above (red, n = 57) 3.55 log2-transformed intensity. The threshold is discovered via Partitioning Around Medoids (PAM). P value was determined by log-rank (Mantel–Cox) test. ‘n’ represents number of patients. **g, h**, Pearson’s correlation coefficient of *CREB1*, *STAT3*, *STAT5A*, and *GATA2* with *PRMT9* levels across TCGA AML cohort (g). Correlation of *CREB1* with *PRMT9* levels across TCGA DLBCL cohort (h). Statistics were determined by pairwise gene correlation analysis as described by GEPIA. Data was sourced from GEPIA. **i**, Expression level of *Creb1* in LSC (n = 383 cells) and Blast (n = 1444 cells) based on MA9 AML scRNAseq analyses. P value was determined by unpaired two-sided t test. **j**, PRMT9 levels after *CREB1* KD. n = 1. **k**, Anti-CREB1 and anti-H3K27Ac ChIP-seq analysis in Molm13. **l**, Upper panel, diagram showing predicted CREB1 binding sites on TSS site in the *PRMT9* promoter. Red bars indicate regions representing CBS (CREB1 Binding Site) and Ctrl sites after ChIP assay. Lower panel, one representative result of ChIP-qPCR analysis of enrichment of CREB1 and H3K27Ac at the CBS site and at a distal control site in Molm13 and normal PBSC CD34+ cells. Similar results were generated from three independent experiments. **m, n**, *PRMT9* mRNA levels in human (m) or murine (n) hematopoietic subset as indicated. Human HSCs are from GSE17054; GMP and MEP cells are from GSE11864; monocytes are from GSE11864 and E-MEXP-1242. Muirne *Prmt9* levels were normalized to Myeloid (Gr1+Mac1+) *Prmt9* mRNA levels and log2-transformed. Data was sourced from Bloodspot. **o**, Schema of the *Prmt9* targeting strategy. The lower left panel shows representative genotyping results of *Prmt9* wt, floxed and KO alleles. Lower right panel, Prmt9 protein levels in BM cells from Prmt9 WT and KO mice. **p–q**, Frequency of hematopoietic progenitors (p) and mature lineage cells (q) in mouse BM at 16 weeks after pIpC administration. (p), *Prmt9* WT (n = 6), *Prmt9* KO (n = 9); (q), *Prmt9* WT (n = 8), *Prmt9* KO (n = 15). ‘n’ represents mice number in each group. Data were presented as mean ± SEM. P values were determined by unpaired two-sided t test. **r**, Competitive transplantation of CD45.2 *Prmt9* KO BM cells with normal CD45.1 BM cells in recipient mice, n = 7 mice/group. The percentage of CD45.2 in PB was assessed. Results represent the mean ± SEM. P was determined by two-way ANOVA. Source data

We assessed PRMT levels using The Cancer Genome Atlas (TCGA) program and cancer cell lines proteomic datasets^11,12^. Among deadly cancers^13^, AML showed the highest *PRMT9* mRNA levels (Fig. 1a,b and Extended Data Fig. 1a). Other PRMTs showed comparable levels in AML and other cancers (Fig. 1a and Extended Data Fig. 1b–n). We observed elevated PRMT9 protein levels in AML relative to other cancers (Fig. 1b and Extended Data Fig. 1o,p). We next assessed all PRMT levels using a dataset that included transcriptome from leukemia and normal hematopoietic stem and progenitor cells (HSPCs)^14^. Notably, *PRMT9* levels were higher in leukemia stem cells (LSCs) relative to either normal hematopoietic stem cells (HSCs) or blasts (Fig. 1c). We next performed single-cell RNA sequencing (scRNA-seq) analysis on murine leukemic bone marrow from an MLL-AF9 (MA9) cKit^+^ cell transplant mouse model. Among the leukemia cells, we identified two clusters as LSCs and blasts (Fig. 1d,e and Extended Data Fig. 1q)^15^. Notably, *Prmt9* was the only *Prmt* whose levels showed more than a onefold increase in LSCs relative to blasts (Fig. 1f). Next, we assessed PRMT9 levels in AML specimens and normal healthy donors (peripheral blood stem cells (PBSCs)). Notably, elevated PRMT9 protein levels were seen in an LSC-enriched (CD34^+^CD38^−^) relative to a leukemia committed progenitor (CD34^+^CD38^+^) subset or to either of the normal subsets (Fig. 1g). We also found that PRMT9 protein levels were higher in the AML CD34^+^ subsets (*n* = 30; Supplementary Table 1) relative to the normal counterparts (*n* = 10) (Fig. 1h,i). Analysis of CD34^+^ cells from another cohort with AML (*n* = 94; Supplementary Table 2) and normal donors (*n* = 19) confirmed *PRMT9* upregulation (Extended Data Fig. 1r). Analysis of another dataset (*n* = 463) showed similar results (Extended Data Fig. 2a). *PRMT9* levels were not associated with any particular cytogenetic abnormality or mutation (Extended Data Fig. 2b–d). PRMT9 protein levels were also higher in diffuse large B cell lymphoma (DLBCL) cell lines (Extended Data Fig. 1o,e)^11^. We also found that higher *PRMT9* levels predicted shorter overall survival (Fig. 1j,k). Analysis of the pediatric dataset yielded similar results (Extended Data Fig. 2f).

We next analyzed chromatin immunoprecipitation followed by sequencing (ChIP–seq) data in ChIPBase v.2.0 (ref. ^16^) and observed the binding sites of relevant transcription factors within 5 kb upstream of the *PRMT9* transcription start site. *CREB1*, a known prognosticator^17^, showed the strongest correlation with *PRMT9* expression in the AML and DLBCL cohorts (Extended Data Fig. 2g,h). Interestingly, we observed an increase in *Creb1* in MA9 LSCs relative to blasts (Extended Data Fig. 2i). *CREB1* inhibition decreased *PRMT9* levels (Extended Data Fig. 2j). We next verified the significant enrichment of *CREB1* in Molm13 cells at the *PRMT9* promoter region relative to a control site (Extended Data Fig. 2k,l); the promoter region showed significant enrichment of H3K27Ac relative to normal cells (Extended Data Fig. 2l).

### *PRMT9* is dispensable for normal hematopoiesis

*PRMT9* levels were higher in HSPCs than in mature lineages^18^ (Extended Data Fig. 2m,n). We developed a conditional knockout (KO) model by crossing *Prmt9*^*loxP*/*loxP*^ with *Mx1-Cre* mice (*Mx1-Cre/Prmt9*^*loxP*^^/^^*loxP*^ or *Prmt9-cKO*). In this model, the *Prmt9 exon2* was flanked by *loxP* sites (Extended Data Fig. 2o). Sixteen weeks after pIpC administration, we observed no differences in complete blood count between *Prmt9-cKO* mice and littermate (*Prmt9*^*loxP*/*loxP*^) controls. *Prmt9* KO modestly increased the multipotent progenitor population and did not affect mature cells (Extended Data Fig. 2p,q). To evaluate the repopulation capacity of HSPCs with *Prmt9* KO, we performed a competitive transplantation. *Prmt9* KO modestly affected CD45.2 chimerism in recipients relative to controls (Extended Data Fig. 2r), suggesting that *Prmt9* function is modestly required for HSPC self-renewal under transplantation stress.

### PRMT9 ablation impairs cancer cell survival

**Fig. 2:**
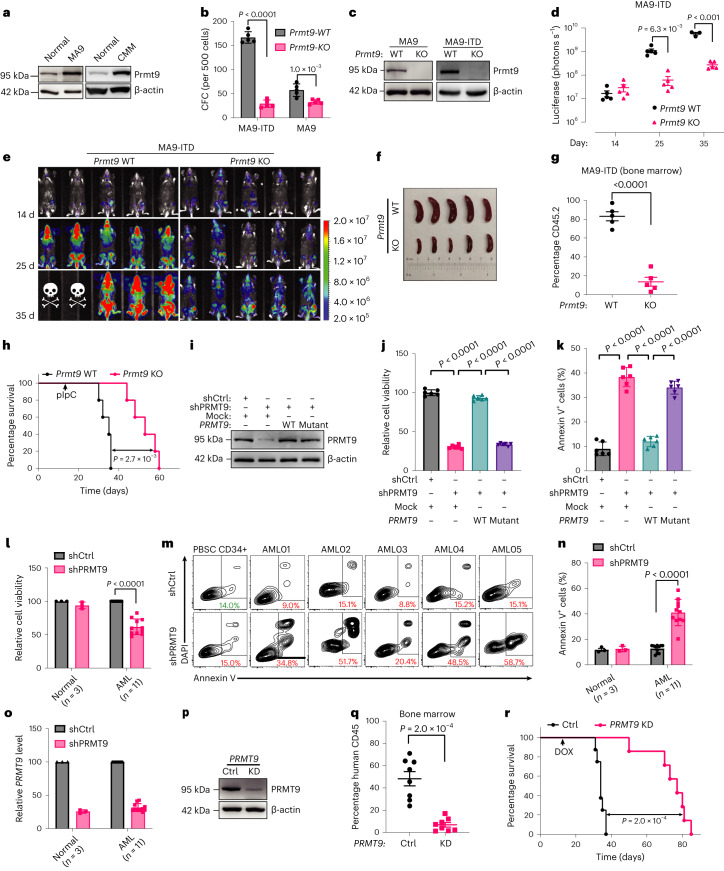
PRMT9 ablation impairs cancer cell survival. **a**, Prmt9 levels in cKit^+^ bone marrow cells from MA9 and CMM mice relative to normal counterparts (*n* = 2 independent experiments). **b**,**c**, CFC of MA9 and MA9-ITD cells after *Prmt9* KO (*n* = 5 independent cultures). **b**, Colony number after *Prmt9* deletion induction, as described previously^76^. Data represent the mean ± s.d. The *P* value was determined using an unpaired two-sided *t*-test. **c**, KO efficiency. **d**,**e**, MA9-ITD-luciferase cells (0.5 × 10^6^ cells per transplant) were injected into irradiated recipients (*n* = 5 mice per group). After engraftment, mice were treated with pIpC and assessed for engraftment using imaging (**e**). **d**, Quantitative results. Data represent the mean ± s.e.m. *P* values were established using a two-way analysis of variance (ANOVA). Color bar, luminescence radiance (photons s^−^^1^ cm^−^^2^ sr^−^^1^). **f**,**g**, Another cohort established as **d**, at the endpoint, spleen (**f**) of *Prmt9* KO and control mice was checked, and engraftment was evaluated based on the percentage of CD45.2^+^ cells (**g**) (*n* = 5 mice per group). Data represent the mean ± s.e.m. **g**, The *P* value was determined using an unpaired two-sided *t*-test. **h**, Survival analysis of MA9-ITD transplants (*n* = 5 mice per group) on *Prmt9* KD. The *P* value was determined using a log-rank (Mantel–Cox) test. **i**–**k**, Molm13 were transduced with mock or *PRMT9* WT or *PRMT9* mutant vectors resistant to shPRMT9; PRMT9 expression was detected after endogenous PRMT9 was knocked down (**i**) (*n* = 2 independent experiments). Cell viability (**j**) using an MTS assay, and apoptosis (**k**), based on annexin V staining (**j**,**k**, *n* = 6 independent experiments). **j**,**k**, Data represent the mean ± s.d. The *P* value was determined using a one-way ANOVA. **l**–**o**, AML CD34^+^ (*n* = 11 patients) or PBSC CD34^+^ (*n* = 3 healthy donors) cells were transduced with shPRMT9. Cell viability (**l**) and apoptosis (**m**,**n**) are shown. *PRMT9* levels were evaluated using quantitative PCR (qPCR) analysis (**o**). **l**,**n**,**o**, Data represent the mean ± s.d. **l**,**n**, *P* values were determined using an unpaired, two-sided *t*-test. **p**,**q**, Molm13 cells transduced with a DOX-inducible shPRMT9 were transplanted into *NSG* mice (control, *n* = 8 mice; *PRMT9* KD, *n* = 7 mice). KD efficiency was evaluated (**p**). After engraftment, mice were treated with DOX to induce *PRMT9* KD and engraftment was evaluated based on the percentage of human CD45 cells (**q**). Data represent the mean ± s.e.m. **q**, The *P* value was determined using an unpaired two-sided *t*-test. **r**, In parallel, survival was analyzed. **r**, *P* was determined using a log-rank (Mantel–Cox) test. Source data

**Extended Data Fig. 3:**
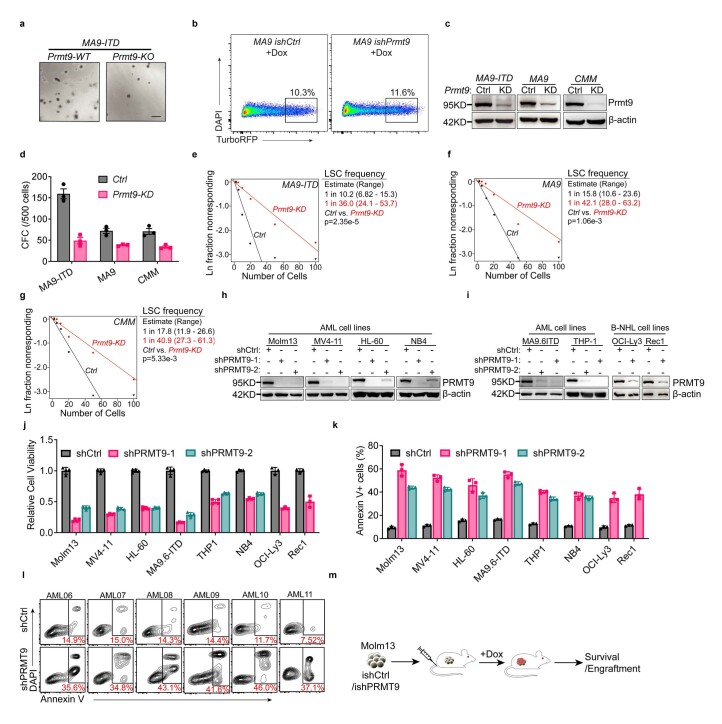
PRMT9 ablation impairs cancer cell survival. **a**, Representative image of colonies in MA9-ITD cells (n = 5 biological replicates). Scale bar: 1000 µm. **b**, Representative plots show the gating for MA9 cells transduced with Dox-inducible shCtrl and shPrmt9. **c, d**, *Prmt9* KD efficiency in indicated cells, were shown (c). CFC of indicated cells after *Prmt9* KD (d). Results represent the mean ± SD from 3 independent experiments. **e–g**, *in vitro* limiting dilution assay (LDA) assay to evaluate the LSC frequency in *Prmt9* KD engineered MA9/FLT3-ITD+ (e), MA9 (f) and CMM (g) AML cells. LSC frequency and p value were calculated using Extreme Limiting Dilution Analysis (ELDA). LSC frequencies are presented as mean ± 95% confidence interval are shown; ELDA were used to analyze χ2 test with 1 degree of freedom. **h–k**, Cancer lines as indicated were transduced with shPRMT9 to KD endogenous *PRMT9*. Cell viability (j) by an MTS assay. Apoptosis (k) by Annexin V staining. PRMT9 expression levels were detected (h, i). Results (j, k) represent the mean ± SD from at least 3 (J, n = 4; K, n = 3) independent experiments. **l**, Primary AML CD34+ cells were transduced with shPRMT9-1 and analyzed for apoptosis as above. **m**, Schema of PRMT9 inducible KD Molm13 xenografted in *NSG* mice. Briefly, Molm13 cells were transduced with a DOX-inducible shPRMT9 or control vector and transplanted into mice. Once engraftment was confirmed (>1% in PB), mice were treated with DOX to induce *PRMT9* KD. At endpoint, engraftment was evaluated based on percentage of hCD45 cells in BM. In parallel analysis, mouse survival was analyzed. Source data

We generated *Prmt9*-cKO/MA9 mice. We observed that *Prmt9* levels are elevated in MA9 mouse bone marrow cKit^+^ cells (Fig. 2a). Given that other oncogenes cooperate with MA9 cells to induce AML^19–21^, we assessed the effects of *Prmt9* KO in both MA9 single-hit and double-hit models; for the latter, we used *FLT3*-internal tandem duplication (ITD) as the second hit^22,23^. Specifically, we transduced Lin^−^Sca-1^+^cKit^+^ bone marrow cells from *Prmt9*-cKO/MA9 or control (*Prmt9* WT/MA9) mice with either a lentiviral vector expressing *FLT3*-ITD and coexpressing green fluorescent protein (GFP). Relative to the controls, colony-forming cell (CFC) growth was inhibited in *Prmt9* KO *MA9*^*+*^ bone marrow cells; more inhibition by *Prmt9* KO was seen in *FLT3*-ITD-expressing cells than in mock cells (Fig. 2b,c and Extended Data Fig. 3a). Additionally, we also transduced a doxycycline (DOX)-inducible *Prmt9* knockdown (KD) construct into MA9 cells. Those inducible constructs coexpressed red fluorescent protein (RFP) (Extended Data Fig. 3b). Consistent with the effect of *Prmt9* KO, *Prmt9* KD significantly inhibited CFC growth of MA9 and MA9/*FLT3*-ITD bone marrow cells (Extended Data Fig. 3c,d). Besides the MA9 model, we evaluated outcomes by inducing *Prmt9* KD in another CBFB-MYH11 knock-in AML model plus thrombopoietin receptor transduction (namely, CMM)^24,25^ resembling inv (16) AML. *Prmt9* levels were significantly elevated in the AML cells (Fig. 2a). *Prmt9* KD inhibited the CFC growth of CMM cells (Extended Data Fig. 3c,d). *Prmt9* KD decreased LSC frequency, as shown in an in vitro limiting dilution assay^26–28^, in all three models (Extended Data Fig. 3e–g).

We transduced MA9/*FLT3*-ITD double-hit *Prmt9* control KO cells (or controls) with a luciferase reporter. Compared to control mice, *Prmt9*-deficient mice had a slower AML progression, lower tumor burden, decreased splenomegaly and survival advantage (Fig. 2d–h).

We next assessed PRMT9 function in human cancers. KD of *PRMT9* (shPRMT9-1, shPRMT9-2) decreased cell growth and viability (Extended Data Fig. 3h–k). We also engineered Molm13 cells to express either a wild-type (WT) *PRMT9* or corresponding catalytically dead mutant^29^; both were designed to resist *PRMT9* short hairpin RNA (shRNA) 1 (that is, PRMT9/WT-R and PRMT9/MUT-R; Fig. 2i). Notably, unlike transduction with PRMT9/MUT-R, PRMT9/WT-R reversed the inhibition seen after *PRMT9* KD (Fig. 2j,k), indicating the requirement for catalysis. Moreover, *PRMT9* KD decreased the viability of AML CD34^+^ cells more potently than it did with normal counterparts (Fig. 2l–o and Extended Data Fig. 3l). Molm13 cells with the DOX-inducible shPRMT9 construct were transplanted into NOD scid gamma (*NSG*) mice (Extended Data Fig. 3m). Once engraftment was confirmed (>1% in peripheral blood), DOX was administered (Fig. 2p). Notably, mice receiving *PRMT9* KD cells exhibited decreased leukemia burden and prolonged survival, compared with control mice (Fig. 2q,r).

### PRMT9-mediated methylation promotes cell growth

**Fig. 3:**
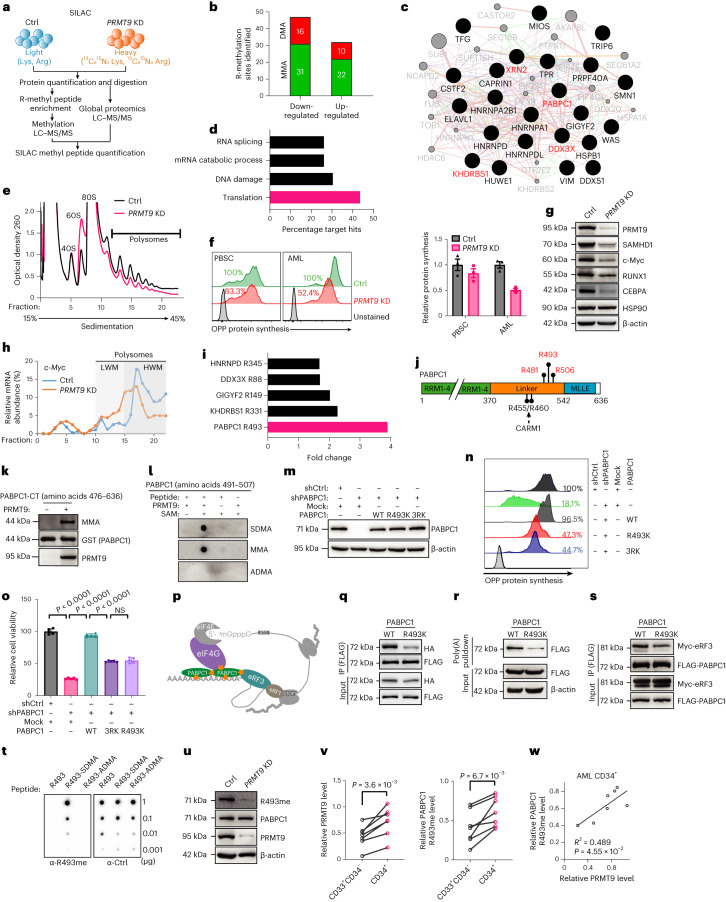
PRMT9-mediated methylation promotes cancer cell growth. **a**, SILAC workflow. **b**, Alteration of sites carrying DMA (red) or MMA (green) on *PRMT9* KD. LC–MS/MS, liquid chromatography–tandem mass spectrometry. **c**, Cytoscape^77^ visualization of proteins carrying PRMT9-regulated R-methyl peptides. **d**, Percentage of hits among all PRMT9-methylated proteins according to Gene Ontology categories. **e**, Polysome profiling of RNAs from control and *PRMT9* KD Molm13 cells. Shown is the representative trace of one of three biological replicates. **f**, AML (*n* = 3 patients) or normal PBSC CD34^+^ (*n* = 3 healthy donors) cells with *PRMT9* KD, analyzed for protein synthesis using an OPP assay. Left: representative. Right: summarized results. Data are the mean ± s.d. **g**, Validation of representative proteins from the SILAC analysis of Molm13 cells (*n* = 2 independent experiments). **h**, *c-Myc* mRNA levels in RNAs extracted from the indicated fractions in ribosome profiling. **i**, Downregulated translation factors with a methylated R site after *PRMT9* KD. **j**, Schematic model of methylated arginine at the PABPC1 C terminus. **k**, In vitro methylation assay of GST-tagged PABPC1-CT mixed with PRMT9. Methylation was analyzed using immunoblotting as indicated (*n* = 2 independent experiments). **l**, Methylation assay of PABPC1 peptides mixed with PRMT9. Methylation was analyzed as indicated (*n* = 3 independent experiments). **m**, Molm13 cells were transduced with mock or PABPC1 (WT, R493K or 3RK) vectors resistant to PABPC1 shRNA; PABPC1 expression was assessed after endogenous *PABPC1* KD (*n* = 2 independent experiments). **n**, Protein synthesis. **o**, Cell viability (*n* = 5 independent experiments). Data are the mean ± s.d. The *P* value was determined using a one-way ANOVA. NS, not significant. **p**, Schematic model of the translation function of PABPC1. **q**, 293T cells were cotransfected with HA-tagged PABPC1 plus FLAG-tagged PABPC1 (WT or R493K). Cell lysates were then subjected to FLAG pull-down and detected using immunoblotting (*n* = 1). **r**, 293T cells were transfected with FLAG-tagged PABPC1 and subjected to poly(A) pulldown, then detected using immunoblotting (*n* = 2 independent experiments). **s**, 293T cells were cotransfected with Myc-tagged eRF3 and FLAG-tagged PABPC1 and subjected to FLAG pulldown, then detected as indicated (*n* = 1). **t**, Indicated amounts of unmodified, SDMA-R493 or ADMA-R493 PABPC1 peptides (amino acids G491–T507) were spotted for a dot blot assay. PABPC1 peptides were detected using anti-R493me-specific or control antibodies. **u**, R493 methylation. PRMT9 levels after *PRMT9* KD in Molm13 (*n* = 2 independent experiments) cells. **v**, PRMT9 and R493 methylation levels in CD34^+^ subsets versus the blast (CD34^−^CD33^+^) subset from the cases with AML (*n* = 7 patients). *P* values were determined using a paired two-sided *t*-test. **w**, Pearson correlation of R493 methylation with PRMT9 levels in AML CD34^+^. The *P* value was determined using simple linear regression analysis. The immunoblot analysis is shown in Extended Data Fig. 5c. Source data

**Extended Data Fig. 4:**
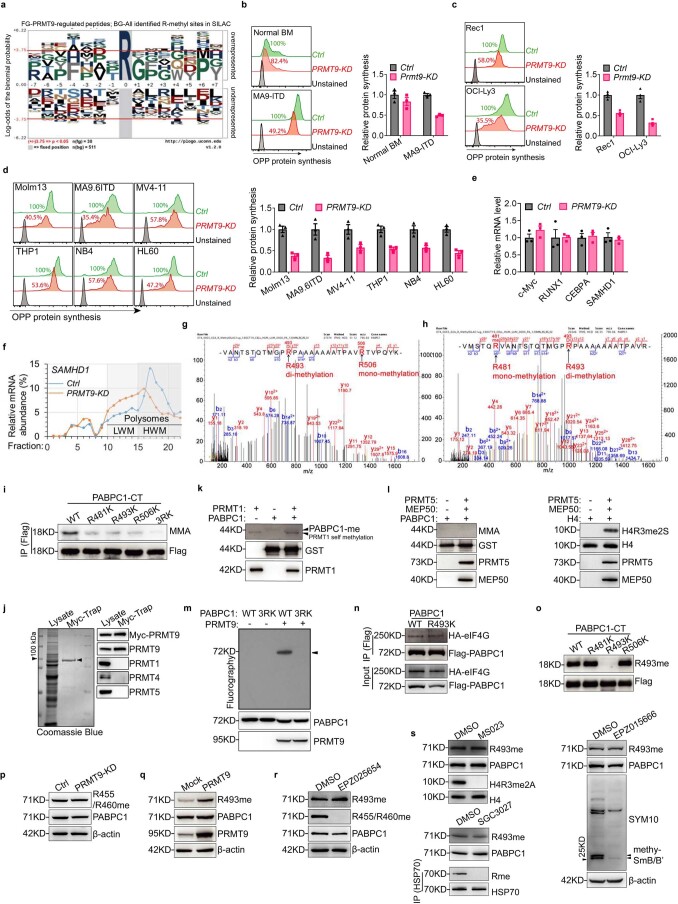
PRMT9-mediated methylation promotes cancer cell growth. **a**, iceLogo motif analysis of R-methyl peptides regulated by PRMT9. **b–d**, Protein synthesis in normal BM cKit + , MA9-ITD cells (b), B-NHL (Rec1 and OCI-Ly3) (c) and indicated AML lines (d) with shCtrl or shPRMT9. The right panel of (b-d) summarizes the results (n = 3 independent experiments). Data were mean ± SD. **e**, Indicated gene levels in Molm13 (n = 3 independent experiments). Data were mean ± SD. **f**, SAMHD1 levels in RNAs extracted from indicated fractions in a ribosome profiling assay. **g, h**, Spectra of R493, R506 and R481 methylation in LC-MS/MS. **i**, 293 T were transfected with Flag-tagged PABPC1-CT, then subjected to immunoprecipitation and immunoblot (n = 2 independent experiments). **j**, Purification of Myc-tagged PRMT9 by the Myc-Trap. Purity-check with indicated PRMTs antibodies, (n = 1). **k**, In vitro methylation assay of GST-tagged PABPC1-CT mixed with PRMT1 protein, SAM, then analyzed by immunoblot (n = 2 independent experiments). **l**, In vitro methylation assay of GST-tagged PABPC1-CT mixed with PRMT5/MEP50, SAM, then analyzed by immunoblot (n = 2 independent experiments). **m**, Ex-vivo tritium methylation assay using PABPC1-WT and -3RK (R481K/R493K/R506K) immunoprecipitated from 293 T (n = 2 independent experiments). **n**, 293 T were co-transfected with HA-tagged eIF4G plus Flag-tagged PABPC1, then subjected to pull-down and immunoblot (n = 1). **o**, 293 T were transfected with Flag-tagged PABPC1-CT, then subjected to pull-down and immunblot (n = 2 independent experiments). **p**, PABPC1 R455/R460 methylation, after *PRMT9* KD in Molm13 (n = 2 independent experiments). **q**, PABPC1 R493 methylation, after PRMT9 overexpression in Molm13 (n = 2 independent experiments). **r**, PABPC1 methylation, in Molm13 treated with EPZ056544 at 5 µM for 48 hr (n = 2 independent experiments). **s**, PABPC1 R493 methylation, in Molm13 cells treated with PRMT1i (MS023, 5 µM, 48 hr), PRMT5i (EPZ015666, 5 µM, 96 hr) or PRMT7i (SGC3027, 5 µM, 48 hr). H4R3me2A, HSP70me (IP HSP70 and detected with anti-MMA antibody), SDMA (SYM10 antibody) were positive controls for MS023, SGC3027, and EPZ015666 respectively (n = 2 independent experiments). Source data

We performed stable isotope labeling by amino acids in cell culture (SILAC)-based proteomics analysis on inducible shPRMT9-transduced or shCtrl-transduced Molm13 cells (Fig. 3a and Supplementary Tables 3 and 4). The analysis revealed 315 unique mono-methylation arginine (MMA) and 109 dimethylation arginine (DMA) sites. *PRMT9* KD resulted in marked downregulation of 16 (14.7%) DMA and 31 (9.8%) MMA sites (Fig. 3b, fold change > 1.5) in 23 unique proteins (Fig. 3c and Supplementary Table 4). However, iceLogo analysis^30,31^ did not detect any consensus sequences enriched in methyl peptides (Extended Data Fig. 4a). Among 23 proteins, ten functioned in RNA translation, seven were related to the DNA damage response and six were related to RNA catabolism (Fig. 3d and Supplementary Table 5).

We next asked whether PRMT9 regulates translation. Specifically, a sucrose density gradient assay revealed that *PRMT9* KD decreased polysome-related mRNAs levels, indicating insufficient mRNA translation^32^ (Fig. 3e). Next, through an O-propargyl-puromycin (OPP)-based assay^33^, we found decreased global protein synthesis after *PRMT9* KD in cancer cells (Fig. 3f and Extended Data Fig. 4b–d), while protein synthesis in normal CD34^+^ cells was modestly altered (Fig. 3f and Extended Data Fig. 4b). *PRMT9* KD downregulated the levels of short-lived proteins (Supplementary Table 3), while their mRNA levels were unchanged (Fig. 3g and Extended Data Fig. 4e). We also observed that *PRMT9* KD shifted *c-Myc* and *SAMHD1* transcripts from high-molecular-weight (HMW) to low-molecular-weight (LMW) polysomes (Fig. 3h and Extended Data Fig. 4f).

Among the downregulated methylated peptides we identified, the methylated PABPC1 peptide with dimethyl-R493 (R493me2), was most depleted by *PRMT9* KD (Fig. 3i). Posttranslational modifications of PABPC1 are critical for its function^34,35^. We thus defined PABPC1 as a PRMT9 substrate. Analysis of PABPC1 peptides in *PRMT9* KD versus PRMT9 WT cells revealed two more sites, monomethylation at R481 (R481me) and R506 (R506me), enriched in PRMT9 WT cells (Fig. 3j and Extended Data Fig. 4g,h). To validate the modifications, we constructed a FLAG-tagged PABPC1 C-terminal fragment (amino acids 476–636, PABPC1-CT) containing R481, R493 and R506, as well as a corresponding methylation-deficient (R to K) construct for each individual residue (R481K, R493K, R506K) or for all three residues (3RK). Only the 3RK mutation depleted PABPC1-CT methylation (Extended Data Fig. 4i). We next performed an in vitro methylation assay by incubating the glutathione *S*-transferase (GST)-PABPC1 fragment and full-length Myc-tagged PRMT9 (Extended Data Fig. 4j) or other PRMTs with SAM. Only PRMT9 promoted PABPC1-CT methylation (Fig. 3k, Extended Data Fig. 4k,l). We confirmed the methylation by an ex vivo tritium methylation assay (Extended Data Fig. 4m). Moreover, when we incubated a synthesized peptide containing R493 with PRMT9 and *S*-adenosyl methionine **(**SAM), the signal was only seen with either anti-symmetric dimethylarginine (SDMA) or anti-MMA antibody (Fig. 3l).

To assess the function of PABPC1 methylation, we ectopically expressed full-length PABPC1 WT, PABPC1-3RK or the PABPC1-R493K mutant, which are resistant to shPABPC1, and further knock down endogenous PABPC1 (Fig. 3m). WT PABPC1 rescued the *PABPC1* KD phenotypes; 3RK or R493K marginally rescued the outcomes seen after *PABPC1* KD (Fig. 3n,o). Moreover, cells expressing R493K exhibited impaired protein synthesis to the same extent as cells expressing 3RK. R493 is located in the region that mediates PABPC1 self-association on the 3′ RNA poly(A) tail^36^; thus, we asked if R493 methylation favored self-interaction (Fig. 3p). Notably, R493K pulled down less hemagglutinin (HA)-tagged PABPC1 than PABPC1 WT (Fig. 3q). Next, we performed a poly(A) agarose pull-down assay to evaluate whether R493 methylation altered PABPC1 binding to poly(A)^34^. Less FLAG-tagged PABPC1 was pulled down from R493K-expressing cells than from PABPC1-WT-expressing cells (Fig. 3r). Furthermore, because R493 is near the PABPC1 C terminus (amino acids 541–636), we asked if R493 methylation facilitated binding of the C terminus to other translational factors, such as eRF3 (ref. ^37^). We analyzed the interaction of FLAG-tagged PABPC1 variants with Myc-tagged eRF3 using co-immunoprecipitation (co-IP). R493K exhibited less affinity to eRF3 than PABPC1 WT (Fig. 3s). In contrast, R493K did not alter the PABPC1 interaction with eIF4G (Extended Data Fig. 4n), which binds to the N terminus^38^.

We next generated an antibody to detect symmetrically dimethylated R493 (R493me) (Fig. 3t). Moreover, mutation of only R493, but not R481 or R506, completely abolished the methylation signals (Extended Data Fig. 4o). Knockdown of endogenous *PRMT9* blocked PABPC1 R493 methylation but not R455 or R460 methylation (Fig. 3u and Extended Data Fig. 4p), while *PRMT9* overexpression increased R493 methylation (Extended Data Fig. 4q). Moreover, inhibitors of other PRMTs did not alter R493 methylation levels (Extended Data Fig. 4r,s). CARM1 catalyzed PABPC1 R455 and R460 methylation did not cross-regulate R493 methylation (Supplementary Fig. 1a,b). Consistent with other reports^35^, CARM1-dependent methylation did not alter RNA translation (Supplementary Fig. 1c,d).

**Extended Data Fig. 5:**
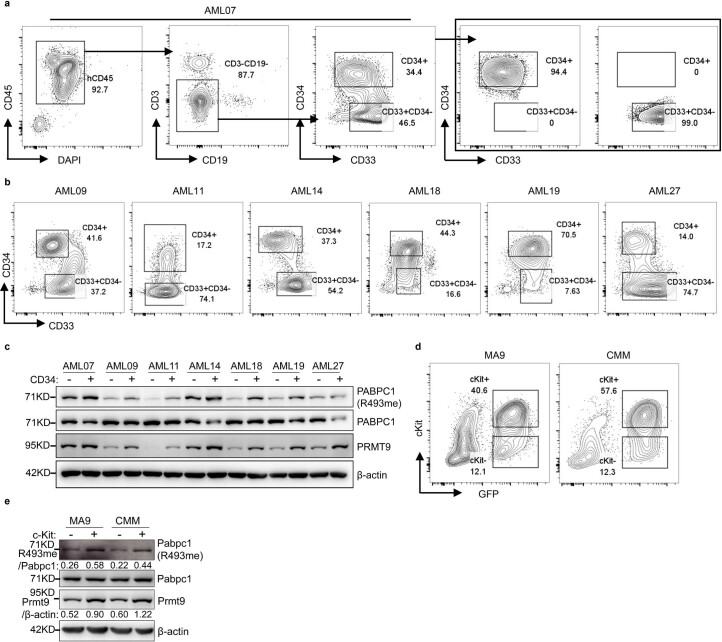
PRMT9 expression is correlated with R493 methylation. **a**, representative gating strategy of human AML sample excluding T cells and B cells, and sorting CD34+ subset and CD34-CD33+ leukemia blasts (Blasts). After sorting, the purity of LSC and Blast cells were checked again. **b**, Sorting strategy of six primary AML samples for the LSC and Blasts (n = 6 samples). **c**, PABPC1 R493me, PRMT9 levels in CD34+ subset and Blasts (n = 7 patient samples). **d**, Sorting strategy of cKit+ and cKit- indicated leukemia cells from BM of AML developed mice. **e**, Levels of PABPC1 R493me, Prmt9 in cKit+ and cKit- leukemia cells as indicated (n = 3 independent experiments). Source data

We sorted the leukemia CD34^+^ subset and CD34^−^CD33^+^ blasts from specimens (*n* = 7; Extended Data Fig. 5a–c). Notably, CD34^+^ cells expressed higher PRMT9 and R493me levels than blasts (Fig. 3v). R493me and PRMT9 levels were positively correlated (Fig. 3w). We conducted similar analyses in MA9 and CMM cells and observed higher PRMT9 and R493me levels in cKit^+^ cells relative to cKit^−^ cells (Extended Data Fig. 5d,e).

### Identification of a PRMT9 inhibitor

**Fig. 4:**
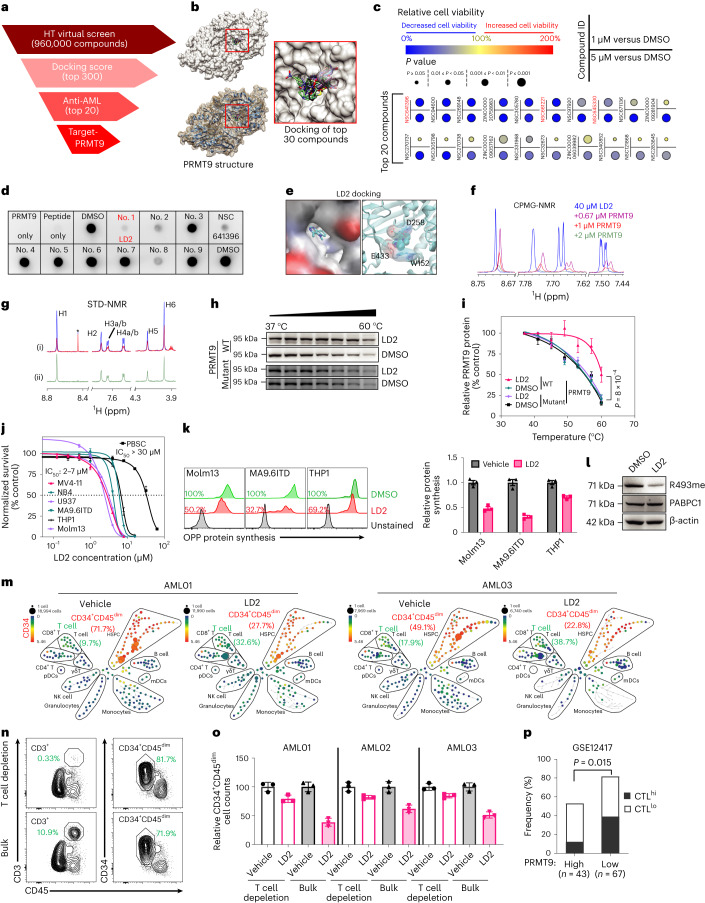
Identification of a PRMT9 inhibitor. **a**, Screening pipeline. HT, high throughput. **b**, Docking pose of the top 30 hits. **c**, Effects of the top 20 compounds on Molm13 viability. **d**, Screening of nine compounds using the R493 methylation assay. Catalytic activity was assessed using a dot blot assay with an anti-R493-specific antibody. No. 1: LD2. **e**, Three-dimensional docking model. Left: LD2 in the pocket. Right: LD2 binding sites. **f**, CPMG NMR for 40 μM LD2 (blue); LD2 in the presence of PRMT9. **g**, STD NMR. (i) Reference (blue) and saturated (red) spectra. (ii) STD spectrum showing the difference between reference and saturated spectra. Asterisk denotes impurity. **h**,**i**, Thermal shift assay (**h**) and relative PRMT9 protein (**i**) of WT mutant PRMT9 from Molm13 cells treated with 2.5 μM LD2. The catalysis inhibition curves are based on the gray intensity of blots normalized to intensity at 37 °C (*n* = 3 independent experiments). A comparison was made between LD2-treated PRMT9 WT versus LD2-treated PRMT9 mutant. **i**, Data (*n* = 3 independent replicates) are represented as the mean ± s.d. *P* values were determined using a two-way ANOVA. **j**, Half-maximal inhibitory concentration (IC_50_) of LD2 in the indicated cells. Cells were treated for 4 days with LD2. MV4-11 (*n* = 6), NB4 (*n* = 3), U937 (*n* = 3), PBSC CD34^+^ (*n* = 6), MA9.6ITD (*n* = 3), Molm13 (*n* = 3) and THP1 (*n* = 4); data are the mean ± s.d. *n* indicates independent experiments and represents the number of independent experiments. **k**, Protein synthesis in the indicated cells after treatment with 2.5 μM LD2, based on an OPP assay. Right: results in vehicle versus LD2 (*n* = 3 independent experiments). Data are presented as the mean ± s.d. **l**, R493 methylation of Molm13 cells treated as indicated (*n* = 3 independent experiments). **m**, CyTOF of AML MNCs after 4 days of treatment with LD2 (2.5 μM). The frequency of T cells and CD34^+^CD45^dim^ AML blast cells was noted. Color bar: CD34 intensity. **n**, Flow plots showing T cell and AML populations in the AML01, before and after T cell depletion. **o**, T cell depleted or bulk MNCs (*n* = 3 patients) were treated with LD2 (2.5 μM). AML blasts were determined using flow cytometry. Data are the mean ± s.d. from three independent experiments. **p**, Frequency of PRMT9^hi^ (*n* = 43) versus low (*n* = 67) AML samples displaying the CTL score high versus low signatures in GSE12417. The *P* value was determined using a two-sided Fisher exact test. *n* represents the number of patients. Source data

**Extended Data Fig. 6:**
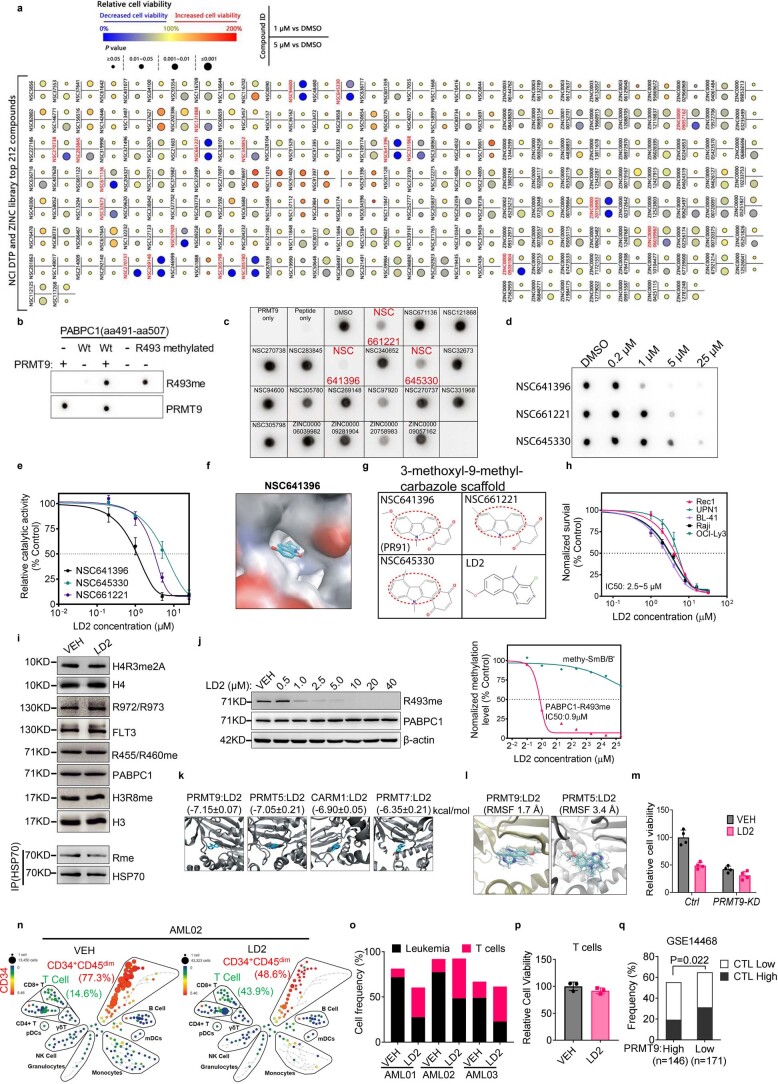
Identification of a PRMT9 inhibitor. **a**, Effects of hits (142 from NCI-DTP and 70 from ZINC library) on Molm13 viability. Cells were treated for 4 days with 1 or 5 μM compounds. For each compound, the number represents its library ID. Experiments were triplicated; P values were derived from t tests. **b**, R493 methylation assay. PABPC1 peptides (G491-T507) were incubated with PRMT9, and SAM. Catalytic activity was assessed by anti-R493 antibody. Synthesized R493me peptide as positive control. **c**, Catalysis screen of top 20 compounds. PRMT9 protein was pretreated with indicated compound (10 μM), then incubated with PABPC1 peptides and SAM. **d, e**, Catalytic activity of indicated compounds based on R493 methylation assay (d). Inhibition curves were calculated based on intensity of dots and normalized to DMSO (e). Data is mean ± SD (n = 3 independent experiments). **f, g**, NSC641396, NSC661221 and NSC645330 share a carbazole ring scaffold. LD2 is designed based on NSC641396 (g). Docking model of NSC641396 in the catalytic pocket (f). **h**, IC50 analysis of LD2 in B-NHL lines. Cells were treated for 4 days with LD2. Data were mean ± SD (n = 6 independent experiments). **i**, Substrates of PRMT1 (H4R3me2A, FLT3 R972/R973me), CARM1 (PABPC1 R455/R460me), PRMT5 (H3R8me2S), PRMT7 (HSP70me) were assessed in Molm13 treated 2 days with 2.5 μM LD2 (n = 2 independent experiments). **j**, Dose-dependent inhibition of PABPC1 R493 methylation level after 2 days of treatment with LD2 in Molm13 cells (n = 1). Cells were treated with a dose titration of 0.5–40 μM LD2 for 48 hr. The inhibition activity of LD2 to PRMT9 (right red curve) was evaluated by calculating the normalized PABPC1 R493 methylation signal. The inhibition activity of LD2 to PRMT5 (right blue curve) was evaluated by calculating the normalized SmB’B’ methylation signal (supplementary Fig. 2). **k**, Vina docking of LD2 with PRMT9, PRMT5, CARM1 or PRMT7 was performed, and the average docking scores (kcal/mol, with standard deviation) were shown. **l**, Molecular dynamics simulation analyses of LD2 with PRMT9 or PRMT5. Smaller root-mean-square-fluctuation (RMSF) of ligand (LD2) in the binding pocket of PRMT9 compared with PRMT5 was shown. **m**, Ctrl and *PRMT9* KD Molm13 were treated for 4 days with LD2 (2.5 uM), and viability was assessed. Data were mean ± SD from 4 independent experiments. **n**, CyTOF of AML MNCs after treatment for 4 days with vehicle or LD2 (2.5 μM). The frequency of CD3 + T cells and CD34+CD45dim AML blasts was noted. **o**, Relative leukemia (CD34+CD45dim) and T cell frequencies before and after LD2 treatment based on CyTOF from 3 samples. **p**, Enriched T cells were treated for 4 days with LD2 (2.5 μM) for 4 days, and cell viability was assessed (n = 3 independent experiments). Data were mean ± SD. **q**, Frequency of PRMT9 high versus low AML samples displaying the Cytotoxic T Lymphocyte (CTL) score high versus low signatures in cohort GSE14468. P value was calculated using two-sided Fisher exact chi-squared test. ‘n’ represents number of patients. Source data

We performed virtual screening. Briefly, we screened compounds for binding affinity to PRMT9; compounds were from the National Cancer Institute (NCI) and ZINC library (Fig. 4a,b). According to the highest binding affinity to the catalytic pocket, we identified top candidates to assess their activity. We used doses of 1 and 5 µM in a Molm13 cell-based viability assay (Extended Data Fig. 6a). We selected the top 20 compounds exhibiting robust cell inhibitory effects for further analysis (Fig. 4c). Specifically, we assessed their effects on PRMT9 catalysis (Extended Data Fig. 6b). Of the 20 compounds tested, three structurally similar compounds showed PRMT9 inhibition (Extended Data Fig. 6c). Among the three, NSC641396 showed the highest inhibition effects (Extended Data Fig. 6d,e).

The NSC641396 docking pose indicated that the quinone ring next to the carbazole moiety extended outside the hydrophobic pocket (Extended Data Fig. 6f). We then conducted a Tanimoto-based two-dimensional similarity search after removing the quinone ring and introducing heteroatoms at different locations of the carbazole moiety (Extended Data Fig. 6g), which yielded 69 compounds. The top nine were purchased; only nos. 1, 2 and 8 showed PRMT9 inhibition efficacy superior to or similar to that of NSC641396, with no. 1 (thereafter called LD2) being the most potent (Fig. 4d,e). Next, we confirmed direct LD2 interaction with PRMT9 protein using nuclear magnetic resonance (NMR) (Fig. 4f,g and Supplementary Table 6). We also assessed the intracellular interaction of the compound and PRMT9 protein using a cellular thermal shift assay^39,40^. Specifically, we engineered Molm13 cells to overexpress FLAG-tagged PRMT9 WT or PRMT9 mutant (W152A, D258A and E433A). All three residues were predicted LD2 binding sites (Fig. 4e). Notably, LD2 treatment led to substantial shifts in the thermal stability of PRMT9 WT but not PRMT9 mutant (Fig. 4h,i).

LD2 treatment preferentially inhibited the viability of cancer cells (Fig. 4j and Extended Data Fig. 6h) and their protein synthesis (Fig. 4k). LD2 at a relatively low dose decreased PRMT9 activity while sparing other PRMTs (Fig. 4l and Extended Data Fig. 6i–j). At a relatively high dose (20 µM), LD2 treatment slightly decreased PRMT5 activity (Supplementary Fig. 2). We also performed docking analysis of LD2 into CARM1, PRMT5, PRMT7 and PRMT9. The relatively lower docking score suggested that LD2 binds PRMT9 (−7.15 kcal mol^−1^) with greater affinity than the other PRMTs tested (Extended Data Fig. 6k). Molecular dynamic simulation analyses confirmed the stronger binding of PRMT9 by LD2 (Extended Data Fig. 6l). Finally, *PRMT9* KD in Molm13 cells reduced their sensitivity to LD2 (at 2.5 µM), suggesting that the LD2 effects at that dose are PRMT9-dependent (Extended Data Fig. 6m).

Next, we treated mononuclear cells (MNCs) from the AML specimens for 4 days^41^ with LD2 (2.5 μM) under physiological cytokine conditions^42^ and then performed cytometry by time-of-flight (CyTOF) analysis. The bone marrow subsets (Supplementary Figs. 3–5) in the vehicle group exhibited modest levels of apoptosis. AML cells from patients (*n* = 3) exhibited expansion of the immature CD34^+^CD45^dim^ subset (Fig. 4m). In these immune and leukemia cell cocultures, LD2 treatment ablated leukemia cells and relatively increased the T cell ratio (Fig. 4m, Extended Data Fig. 6n,o and Supplementary Fig. 4). Notably, LD2 treatment expanded the number of IFN-γ-expressing T cells relative to vehicle controls (Supplementary Fig. 6). We also depleted autologous CD3^+^ T cells of the AML samples (*n* = 3) before treatment (Fig. 4n). Notably, the inhibitory effects of LD2s were partially impaired by the depletion (Fig. 4o). T cell viability was not affected by LD2 treatment (Extended Data Fig. 6p). To analyze the correlation between PRMT9 activity and T cell function, we analyzed the RNA-seq results from GSE12417GSE14468 and used a reported^43^ cytotoxic T lymphocyte (CTL) score. Those scores were negatively correlated with *PRMT9* levels (Fig. 4p and Extended Data Fig. 6q).

### PRMT9 inhibition eradicates AML in vivo

**Fig. 5:**
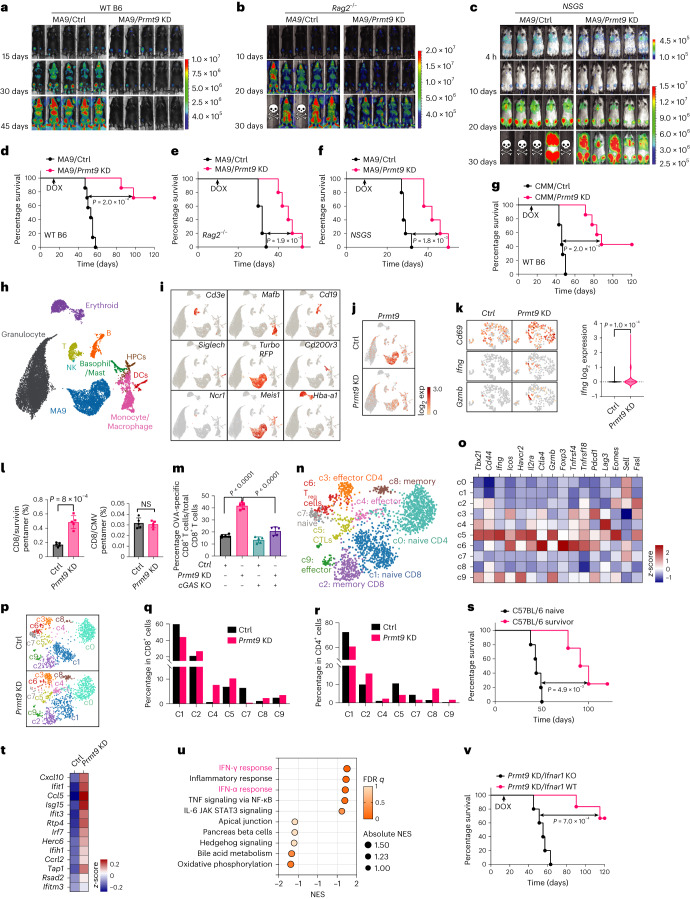
PRMT9 inhibition eradicates AML in vivo. **a**–**c**, MA9-luciferase cells were injected into B6 (**a**, *n* = 7 mice per group), *Rag2*^−*/*−^ (**b**, *n* = 5 mice per group) and *NSGS* (**c**, *n* = 5 mice per group) mice. After engraftment, mice were administered DOX water. Engraftment was tracked using imaging; color bars, luminescence radiance (photons s^−1^ cm^−2^ sr^−1^). **d**–**f**, Kaplan–Meier curves showing the survival of B6 (**d**), *Rag2*^−*/*−^ (**e**) and *NSGS* (**f**) mice. *P* values were determined using a log-rank (Mantel–Cox) test. **g**, CMM cells were injected into B6 mice (*n* = 7 mice per group). *Prmt9* KD was induced as above. The Kaplan–Meier curves show the survival of mice. *P* values were determined using a log-rank (Mantel–Cox) test. **h**,**i**, Different populations (**h**) or markers (**i**) identified in bone marrow. **j**, *Prmt9* level in the bone marrow populations. **k**, *Cd69*, *Ifng* and *Gzmb* levels in T cells of Ctrl (*n* = 249 cells) and *Prmt9* KD (*n* = 231 cells) bone marrow. Right: *Ifng* levels. Data are presented as the mean ± s.e.m. The *P* value was determined using an unpaired two-sided *t*-test. **l**, Frequency of AML-specific CD8^+^ T cells in *Prmt9* KD mice (*n* = 5 mice) relative to *Prmt9* WT controls (*n* = 5 mice). Data are the mean ± s.e.m. The *P* value was determined using an unpaired two-sided *t*-test. **m**, Indicated MA9/OVA cells were implanted into B6 mice (*n* = 5 mice per group). After engraftment, *Prmt9* KD was induced. MA9-OVA-specific T cells were assessed. Data are the mean ± s.e.m. The *P* value was determined using a one-way ANOVA test. **n**, Subpopulations identified among T cells from the spleen in the merged Ctrl and *Prmt9* KD groups. **o**, Expression levels of the indicated genes in the T cell clusters. **p**, Distribution of the clusters annotated in **n**. **q**,**r**, Percentage of clusters in CD8^+^ (**q**) or CD4^+^ (**r**) T cells annotated in **n**. **s**, Survivors of *Prmt9* KDMA9 cell-challenged mice (*n* = 4) were rechallenged with 1 × 10^6^ parental MA9 *Prmt9* KD cells (without DOX induction). Control naive C57BL/6 mice (*n* = 5 mice per group) inoculated with the same number of cells. The Kaplan–Meier curves show the survival of mice. The *P* value was determined using a log-rank (Mantel–Cox) test. **t**, Upregulated ISGs in T cells. **u**, GSEA of DEGs in bone marrow T cells after *Prmt9* KD. **v**, MA9-luciferase cells were injected into WT (*n* = 7 mice) or *Ifnar1* KO mice (*n* = 5 mice). *Prmt9* KD was induced as above. The Kaplan–Meier curves show the survival of mice. The *P* value was determined using a log-rank (Mantel–Cox) test. Source data

**Extended Data Fig. 7:**
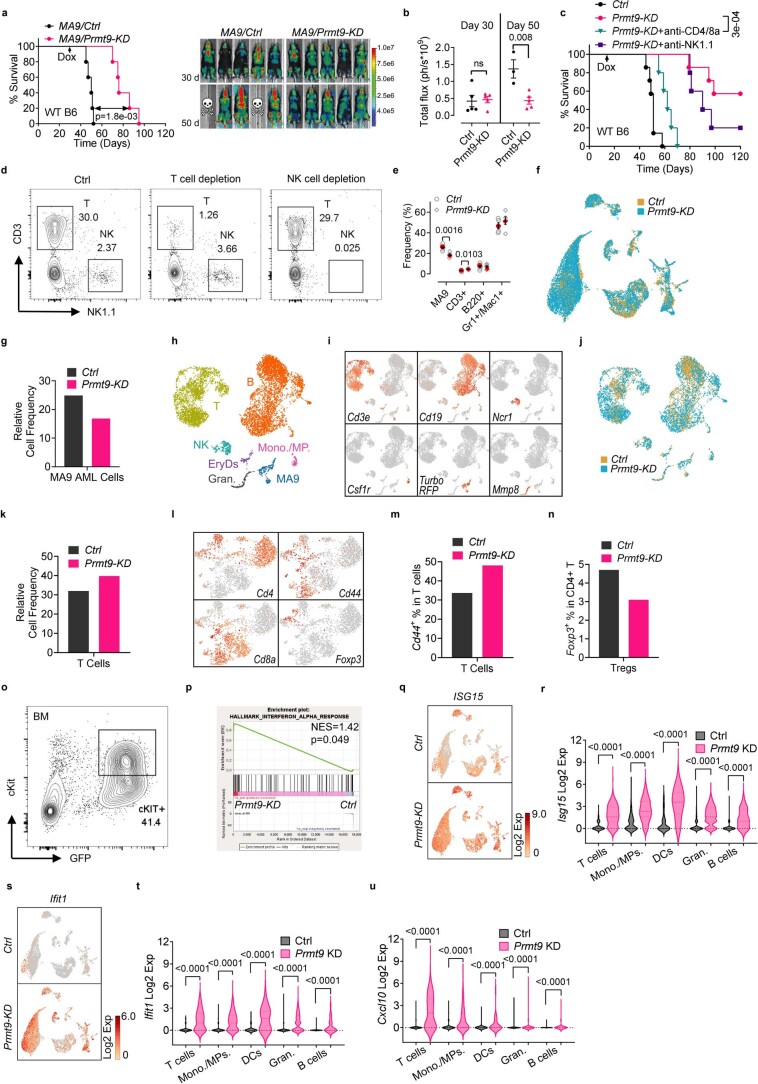
PRMT9 inhibition eradicates AML in vivo. **a**, Control or inducible *Prmt9* KD MA9-lucifase cells were injected into wild-type B6 (1×10^6^ cells per mouse, n = 5/group). After 30 days when leukemia robustly developed, mice were continuously administered with Dox. Kaplan-Meier curves show survival. P value was determined by log-rank (Mantel–Cox) test. **b**, MA9 AML burden was assessed by bioluminescence imaging over indicated days and the statistics for the quantitative results on day 30 and day 50 from bioluminescence imaging were shown, n = 5 mice/group. Data are presented as mean ± SEM. P values were determined by unpaired two-sided t test. **c**, MA9 AML cells we implanted into WT mice and evaluated progression: 1) Ctrl (*Prmt9* WT), n = 7 mice; 2) *Prmt9* KD, n = 7 mice; 3) *Prmt9* KD with T cell depletion, n = 5 mice; 4) *Prmt9* KD with NK cell depletion, n = 5 mice. Kaplan-Meier curves show survival. P value was determined by log-rank (Mantel–Cox) test. **d**, following engraftment, 1 day prior to in-vivo DOX administration to KD Prmt9, mice are administered with anti-CD4/CD8 treatment or anti-NK1.1 to deplete T or NK cells. Plot of the depletion in PB was shown. **e**, Control or *Prmt9* KD MA9-lucifase cells were injected into wild-type B6 mice (1×10^6^ cells per mouse, n = 5). Following engraftment, mice were treated with Dox in drinking water for 7 days. The plot shows the frequency of MA9, CD3 + , B220+ and Gr1 + /Mac1+ cells in BM of each mouse at the time of collection analyzed by flow-cytometry. BM and spleen cells from one representative mouse (with the frequency of each subset highlighted in red) in each group were selected and subjected to scRNA-seq analysis. Data are presented as mean ± SEM. P value of each comparison was determined by unpaired two-sided t test. **f**, identified populations in MA9 BM. **g**, Frequency of AML cells in BM. **h–j**, Different populations (h) and markers (i) identified within Ctrl and *Prmt9* KD groups merged in spleen cells. identified spleen populations are shown (j). **k**, T cell frequency in spleen cells. **l**, Expression of T cell marker genes in spleen. **m**, Frequency of Cd44+ cells in BM T cells. **n**, Frequency of Tregs (Foxp3+) in BM CD4 + T cells. **o**, Plot of leukemic BM in MA9 rechallenge. **p**, GSEA analysis of BM T cells. Normalized enrichment scores and family-wise error rate P-value was determined by the GSEA permutation method. The Normalized Enrichment Score is calculated by dividing the Enrichment Score from the actual ranking by the means of the random permutations. An enrichment P-value is calculated by comparing the observed frequency of an annotation term with the frequency expected by chance; individual terms beyond cut-off (p-value ≤ 0.05) are deemed enriched. **q–u**, (q-r) UMAP (q) and histogram (r) showing Isg15 expression in indicated BM subpopulation; (s-t) UMAP and histogram showing Ifit1 expression in indicated BM population; (u) Cxcl10 expression in indicated BM populations. Ctrl: T cells, n = 249 cells; Monocytes/Macrophages, n = 631 cells; DCs, n = 108 cells; Granulocytes, n = 3378 cells; B cells, n = 413 cells. *Prmt* KD: T cells, n = 231 cells; Monocytes/Macrophages, n = 469 cells; DCs, n = 57 cells; Granulocytes, n = 3906 cells; B cells, n = 134 cells. For (o, q, r), Data were mean ± SEM. P values were determined by unpaired two-sided t-tests. Source data

To assess whether cancer-intrinsic PRMT9 inhibition induces immune responses, we used an MA9 AML transplant model (Fig. 5a,d). These MA9 AML cells were transduced with either a DOX-inducible shPrmt9 or shCtrl construct. Congenic WT *C57BL/6* (B6) mice were used as recipients; *Rag2*^−*/*−^ (Fig. 5b) or *NSG-SGM3* (*NSGS*) (Fig. 5c) mice were also used. After engraftment, DOX was administered. *Rag2*^−*/*−^ (Fig. 5b,e) or *NSGS* (Fig. 5c,f) mice bearing *Prmt9* KD transplants survived significantly longer than mice with *Prmt9* WT transplants, but succumbed to leukemia within 60 days (Fig. 5e,f). In contrast, five of seven B6 mice receiving *Prmt9* KD transplants survived until day 120 (Fig. 5d). We established another cohort of MA9 leukemia transplants using B6 recipients and induced *Prmt9* deletion on day 30. As shown (Extended Data Fig. 7a,b), *Prmt9* KD modestly decreased AML progression.

We also performed antibody-based depletion of T or natural killer (NK) cells before in vivo DOX administration to KD *Prmt9* (Extended Data Fig. 7c,d). CD4 and CD8 T cell depletion significantly abolished *Prmt9* KD-induced AML regression, while NK depletion had minor effects (Extended Data Fig. 7c,d).

To verify the role of *Prmt9* inhibition in a different AML model, we used the CMM transplant model^25^. Thus, *Prmt9* KD-mediated leukemia elimination effects are comparable in both models (Fig. 5g). Notably, *Prmt9* KD remarkably decreased leukemia-initiating cell frequency in both models (Fig. 5d,g and Supplementary Table 7).

We performed scRNA-seq analysis of the MA9 tumor microenvironment. A cohort of WT B6 mice transplanted with DOX-inducible *Prmt9* KD AML cells was established; we evaluated the transcriptional status of all immune lineages in transplants 7 days after DOX treatment. At that time, mice receiving *Prmt9* KD cells began to exhibit decreased AML engraftment (Extended Data Fig. 7e). We then collected bone marrow and spleen cells from a representative mouse in each group for scRNA-seq. Our transcriptomes include 9,741 control and 11,291 *Prmt9* KD bone marrow cells. We visualized transcriptionally homogeneous cell clusters (Fig. 5h,i and Supplementary Fig. 7). Notably, *Prmt9* levels were more abundant in leukemic cells than other cells; *Prmt9* KD decreased tumor cell frequency relative to controls (Fig. 5j and Extended Data Fig. 7f,g) and induced T cell activation (Fig. 5k). To verify T cell function, we assessed leukemia-specific T cell responses after *Prmt9* KD using major histocompatibility complex (MHC)-survivin peptide as described in Stroopinsky et al.^44^. We confirmed the elevated expression of Birc5 (encoding survivin) in cancer relative to the other subsets (Supplementary Fig. 8). We also noted that *Prmt9* KD mice exhibited increased CD8^+^ T cells recognizing tumor survivin relative to *Prmt9* WT controls (Fig. 5l). The results were confirmed using MA9/OVA cells (Fig. 5m).

We analyzed the scRNA-seq results of spleen, where T cells are more abundant (Extended Data Fig. 7h–k). We focused on T cells (Fig. 5n,o and Extended Data Fig. 7l). Accordingly, ten distinct T cell subpopulations (c0–9) were characterized, including c0 (naive CD4^+^), c1 (naive CD8^+^), c2 (memory CD8^+^cells), c3 (effector CD4^+^cells), c8 (memory T cells), c4 and c9 (effector T (T_eff_) cells), c5 (CTLs) and c6 (regulatory (T_reg_) cells) (Fig. 5n,o). Notably, *Prmt9* KD altered the proportions of the subpopulations (Fig. 5o–r). *Prmt9* KD reduced the naive T cell subsets (c0, c1) and expanded the populations of effector and memory T cells (c2, c3, c4, c8 and c9) (Fig. 5q,r). *Prmt9* KD also increased CTLs (c5) and decreased T_reg_ cells (c6) (Fig. 5q,r). In bone marrow, *Prmt9* KD expanded *Cd44-*expressing T cells and decreased *Foxp3*-expressing T_reg_ cells (Extended Data Fig. 7m,n).

To assess immune memory, we selected primary B6 mice that had shown complete regression of MA9 tumors on *Prmt9* KD. We rechallenged them and the naive control cohort by injecting them with comparable numbers of MA9 cells. Unlike the control cohort (Fig. 5s), tumor-free mice exhibited a survival benefit after being rechallenged (Extended Data Fig. 7o).

*Prmt9* KD upregulated IFN-stimulated gene (ISG) levels in T cells (Fig. 5t). Gene set enrichment analysis (GSEA) highlighted the activation of the IFN response pathways in T cells (Fig. 5u and Extended Data Fig. 7p). Similarly, ISG upregulation, including *Isg15* (Extended Data Fig. 7q,r), *Ifit1* (Extended Data Fig. 7s,t) and *Cxcl10* (Extended Data Fig. 7u), was seen in other immune cell types. To verify that type I IFN responses underlined the outcomes, *Prmt9* KD MA9 cells were transplanted into WT recipient or type I IFN receptor KO (*Ifnar1* KO) mice. After leukemia cell engraftment, we induced *Prmt9* KD and monitored leukemia development. The anti-AML effects of *Prmt9* KD were significantly abolished on an *Ifnar1* KO background (Fig. 5v).

### Immunity after PRMT9 inhibition requires cGAS activity

**Fig. 6:**
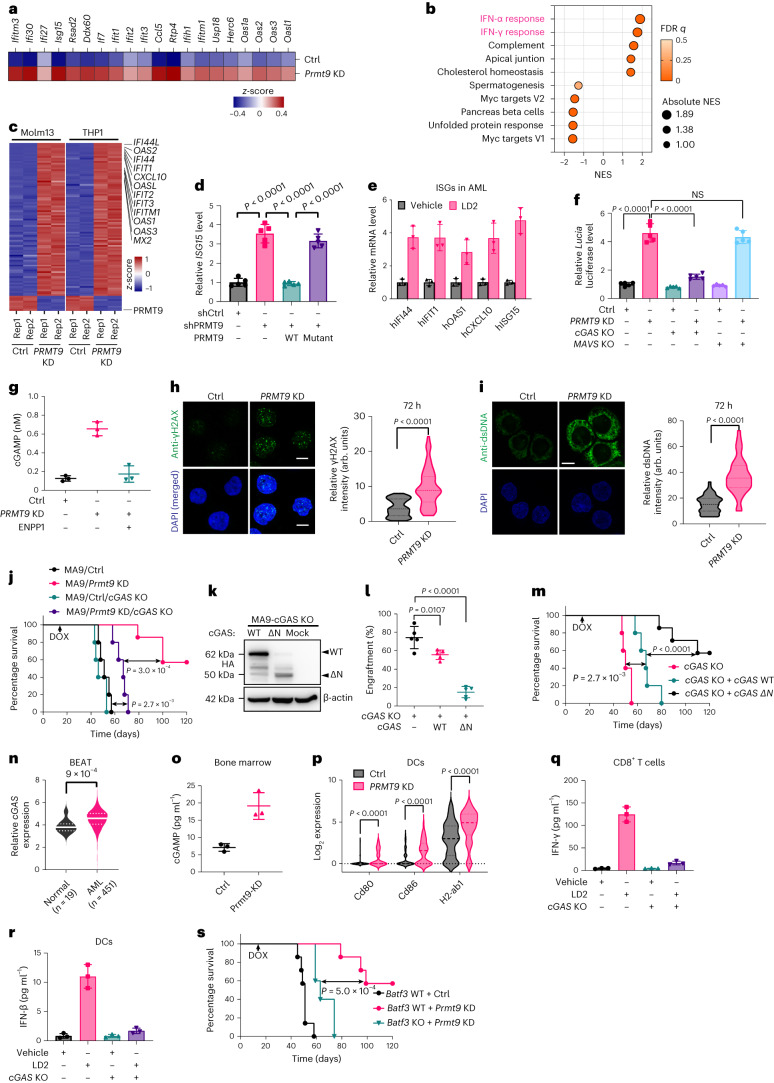
Immunity after PRMT9 inhibition requires cGAS activity. **a**, Upregulated ISGs in MA9 cells. **b**, GSEA of DEGs in *Prmt9* KD MA9 cells. **c**, Overlapped DEGs in the indicated cells (fold change > 2). NES, normalized enrichment score. **d**, *ISG15* expression in Molm13 cells with endogenous *PRMT9* KD and after rescuing with PRMT9 WT or a catalytically dead mutant (*n* = 5 independent experiments). Data are the mean ± s.d. The *P* value was determined using a one-way ANOVA. **e**, ISG levels in AML CD34^+^ cells. Data are the mean ± s.d. from three independent experiments. **f**, Luciferase activity of THP1-IRF cells engineered as indicated (*n* = 5 independent experiments). Data are the mean ± s.d. The *P* value was determined using a one-way ANOVA. **g**, cGAMP levels in engineered THP1 supernatant (*n* = 3 independent experiments). Data are the mean ± s.d. **h**, Left: immunostaining for γH2AX in THP1 cells. Right: γH2AX intensity (*n* = 100 cells per group). Scale bars, 10 μm. The *P* value was determined using an unpaired two-sided *t*-test. **i**, dsDNA using immunostaining in THP1 cells. Right: dsDNA intensity (*n* = 50 cells per group). Scale bar, 10 μm. The *P* value was determined using an unpaired two-sided *t*-test. **j**, MA9/OVA cells (Ctrl, *n* = 5 mice), *Prmt9* KD (*n* = 7 mice), Ctrl + *cGAS* KO (*n* = 5 mice) and *Prmt9* KD + cGAS WT (*n* = 5 mice)) were transplanted to establish AML. *Prmt9* KD was induced. The Kaplan–Meier curves show the survival. *P* values were determined using a log-rank (Mantel–Cox) test. **k**, *cGAS* KO *MA9* cells were transduced with inducible HA-tagged cGAS WT or ΔN. Exogenous cGAS was then assessed (*n* = 1). **l**,**m**, *cGAS* KO (*n* = 5 mice), *cGAS* WT (*n* = 5 mice) or *cGAS-*ΔN MA9 (*n* = 7 mice) cells were transplanted. DOX was given to induce expression of cGAS variants. **l**, AML engraftment was assessed. Data are the mean ± s.e.m. *P* values were determined using a one-way ANOVA. **m**, For another cohort, Kaplan–Meier curves show survival. *P* values were determined using a log-rank (Mantel–Cox) test. **n**, *cGAS* levels in BEAT AML cases (*n* = 451 patients) and healthy donors (*n* = 19). The *P* value was determined using an unpaired two-sided *t*-test. **o**, cGAMP levels in the bone marrow of mice (*n* = 3 mice per group). Data are the mean ± s.d. **p**, Expression of *Cd80*, *Cd86* and *H2-ab1* in the DCs of the scRNA-seq of Ctrl (*n* = 108 cells) and *Prmt9* KD (*n* = 57 cells) bone marrow. *P* values were determined using an unpaired two-sided *t*-test. **q**,**r**, LD2-pretreated MA9/OVA cells were cocultured with bone marrow-derived DCs. DCs were then cocultured with OT-I transgenic CD8^+^ T cells. **q**, IFN-γ production by CD8^+^ T cells. **r**, IFN-β production by DCs. *n* = 3 independent experiments. Data are the mean ± s.d. **s**, MA9 AML cells were implanted into *Batf3* WT or KO mice: (1) *Prmt9* KD/*Batf3* KO (*n* = 5 mice), (2) *Prmt9* KD/*Batf3* WT (*n* = 7 mice) and (3) *Prmt9* WT/*Batf3* WT (*n* = 7 mice). Kaplan–Meier curves show survival. *P* values were determined using a log-rank (Mantel–Cox) test. Source data

**Extended Data Fig. 8:**
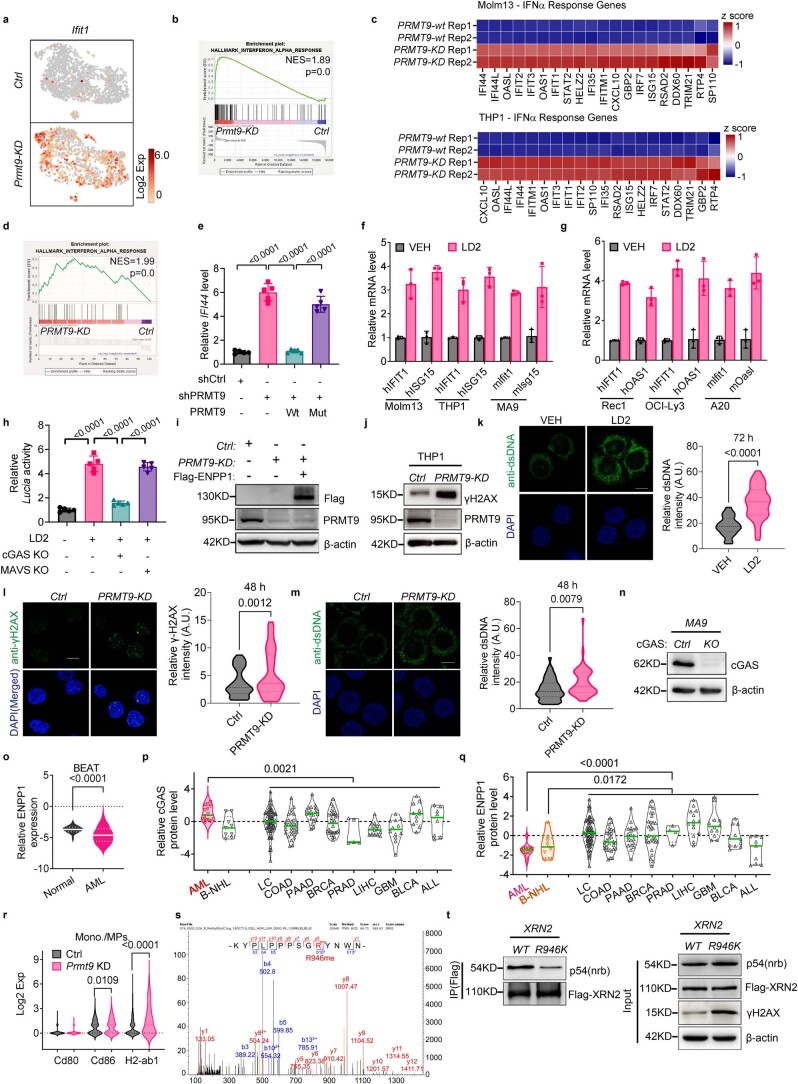
Immunity following PRMT9 inhibition requires cGAS activity. **a**, Ifit1 expression in AML cells from BM scRNA-seq. **b**, GSEA analysis of BM T cells. Normalized enrichment scores and family-wise error rate P-value was determined by the GSEA permutation method. **c, d**, RNA-seq of common DEG in *PRMT9* KD versus Ctrl cell lines. Heatmap (c) and GSEA (d) show upregulation of IFNα response genes upon *PRMT9* KD. The color code represents z scores for differential gene expression. For (b) and (d), the normalized enrichment scores and family-wise error rate P-value was determined by the GSEA permutation method. The Normalized Enrichment Score is calculated by dividing the Enrichment Score from the actual ranking by the means of the random permutations. An enrichment P-value is calculated by comparing the observed frequency of an annotation term with the frequency expected by chance; individual terms beyond cut-off (p-value ≤ 0.05) are deemed enriched. **e**, IFI44 expression in Molm13 engineered as indicated (n = 5 independent experiments). Data were mean ± SD. P value was determined by one-way ANOVA. **f, g**, Levels of selected ISG genes (f) in AML and B-NHL lines after 2 days LD2 treatment (n = 3 independent experiments). Data were presented as mean ± SD. **h**, Lucia activity of engineered THP1 after LD2 treatment (n = 5 independent experiments). Data were mean ± SD. P value was determined by one-way ANOVA. **i**, Levels of PRMT9 and Flag-tagged ENPP1 in engineered THP1 cells, (n = 1). **j**, Levels of γH2AX in THP1 cells, (n = 1). **k**, dsDNA by immunostaining in THP1 cells after LD2 treatment for 72 hr. Violin plots (right) summarized dsDNA intensity (n = 50 cells/group). Scale bar, 10 μm. **l**, Immunostaining for γH2AX in Ctrl and *PRMT9* KD THP1 cells. Violin plots (right) summarized γH2AX intensity (n = 100 cells/group). Scale bar, 10 μm. **m**, dsDNA by immunostaining in Ctrl and *PRMT9* KD THP1 cells. Violin plots (right) summarized dsDNA intensity (n = 50 cells/group). Scale bar, 10 μm. P values (k,l,m) were calculated by unpaired two-sided t test. **n**, Levels of cGAS in Ctrl and cGAS-KO MA9 cells (n = 2 independent experiments). **o**, ENPP1 mRNA levels in the BEAT AML (n = 451 AML cases; n = 19 healthy donors). The P value was by unpaired two-sided t test. **p-q**, cGAS and ENPP1 expression in AML and B-NHL lines compared with other cancer cell lines. AML (n = 14), B-NHL (n = 9), LC (n = 77), COAD (n = 30), PAAD (n = 20), BRCA (n = 30), PRAD (n = 5), LIHC (n = 14), GBM (n = 12), BLCA, (n = 11) and ALL (n = 8). P values were by unpaired two-sided t test. ‘n’ represents number of cell lines. **r**, Cd80, Cd86, H2-ab1 expression in monocytes/macrophages from BM, from scRNA-seq. Ctrl, n = 631 cells; *Prmt9* KD, n = 469 cells. Data were mean ± SEM. P values were determined by unpaired two-sided t-test. **s**, Representative spectrum of XRN2 R946 methylation in SILAC-based methyl-peptide quantitative LC-MS/MS. **t**, 293 T were transfected with Flag-tagged XRN2, then for immunoprecipitation and the interactor detected by immunoblot, (n = 1). γH2AX was detected in the input lysate. Source data

By analyzing the scRNA-seq transcriptomes of MA9 cells, we observed upregulation of multiple ISGs after *Prmt9* KD (Fig. 6a and Extended Data Fig. 8a). GSEA showed top enrichment of the IFN-α and IFN-γ pathways after *Prmt9* KD (Fig. 6b and Extended Data Fig. 8b). Consistently, transcriptome profiling of the AML lines confirmed innate immune signaling activation by targeting Prmt9 (Fig. 6c and Extended Data Fig. 8c,d). To determine if innate immune activation was associated with PRMT9 activity, we compared ISG expression in Molm13 cells overexpressed WT or catalytic mutant PRMT9 after KD of endogenous *PRMT9*. Notably, only expression of PRMT9 WT, but not the mutant rescued ISG upregulation (for example*, ISG15*, *IFI44*) (Fig. 6d and Extended Data Fig. 8e). LD2 treatment also stimulated ISGs expression (Fig. 6e and Extended Data Fig. 8f,g).

We used THP1-*Lucia* luciferase to monitor IFN regulatory factor (IRF) signaling downstream of innate immune sensors, including the double-stranded DNA (dsDNA) sensor cGAS or dsRNA sensors. *PRMT9* KD or LD2 treatment of THP1-*Lucia* luciferase increased luciferase signals (Fig. 6f and Extended Data Fig. 8h); an increase was blocked by deletion of cGAS. *PRMT9* KD also enhanced cGAS activity, as evidenced by increased cGAMP (Fig. 6g). Overexpression of ENPP1, which degrades cGAMP, abrogated this effect (Fig. 6g and Extended Data Fig. 8i). *PRMT9* KD increased γH2AX levels, which is indicative of DNA damage (Fig. 6h and Extended Data Fig. 8j,l), and promoted the accumulation of cytoplasmic dsDNA (Fig. 6i and Extended Data Fig. 8k,m). To determine whether tumor-intrinsic cGAS activity was required for *Prmt9* KD-mediated immunity, MA9-OVA cells with *cGAS* KO (Extended Data Fig. 8n) were transduced with either inducible shPrmt9 or shCtrl and implanted into WT recipients. Unlike controls, *cGAS* KO mice did not show the tumor-specific T cell response seen after *Prmt9* KD (Fig. 5m). Survival advantages were abolished on a *cGAS* KO background (Fig. 6j). To test the outcomes of cGAS activation in cancer cells, we transduced *cGAS* KO MA9 cells with an inducible cGAS-activating mutant (ΔN)^45^ or corresponding *cGAS* WT (Fig. 6k) and then implanted parental (*cGAS* KO), *cGAS* WT or ΔN-expressing cells into B6 mice to analyze leukemogenesis. A reduced AML burden was seen in ΔN transplants (Fig. 6l). Mice with AML cells exhibiting cGAS activation showed significantly extended survival relative to other groups (Fig. 6m). cGAS levels were remarkably high in AML relative to normal donors (Fig. 6n), while ENPP1 levels were relatively lower in AML (Extended Data Fig. 8o). Moreover, among deadly cancers, AML cells exhibited the highest cGAS and lowest ENPP1 levels (Extended Data Fig. 8p,q).

We hypothesized that the T cell priming effects seen after *Prmt9* KD could be mediated by increases in the immune transmitter cGAMP. Indeed, we observed elevated cGAMP levels in bone marrow fluid from *Prmt9* KD MA9 mice (Fig. 6o). Moreover, the single-cell transcriptomes of DCs and macrophages revealed increased levels of *Cd86* and MHC class II (*H2-ab1*), suggesting activation (Fig. 6p and Extended Data Fig. 8r). To determine whether the T cell priming effects seen after Prmt9 inhibition were due to antigen cross-presentation by DCs, we cocultured LD2-pretreated *cGAS* KO or *cGAS* WT MA9/OVA cells with bone marrow-derived DCs and then purified the DCs, which were exposed to naive OT-I^+^CD8^+^ T cells. We observed increased IFN-γ production by CD8^+^ T cells after coculture with DCs purified from the LD2-pretreated AML group (Fig. 6q). *cGAS* KO antagonized the DC-mediated cross-priming capacity, based on decreased IFN-γ production (Fig. 6q). We then assessed IFN-β production by DCs after coculture with MA9-OVA cells pretreated with LD2 and observed increased IFN-β production (Fig. 6r), an effect abolished by *cGAS* KO, suggesting that tumor *cGAS* activation underlies type I IFN signaling in DCs. We further assessed DC function in *Prmt9* KD-induced AML regression using *Batf3* KO mice because Batf3 is critical to cross-prime T cells^46^. Specifically, we implanted *Batf3* WT or KO mice with AML cells for assessment of AML progression. Relative to *Batf3* WT mice, *Batf3* KO partially decreased the *Prmt9* KD-induced survival advantage (Fig. 6s).

### Loss of XRN2 methylation underlies *cGAS* activation

**Fig. 7:**
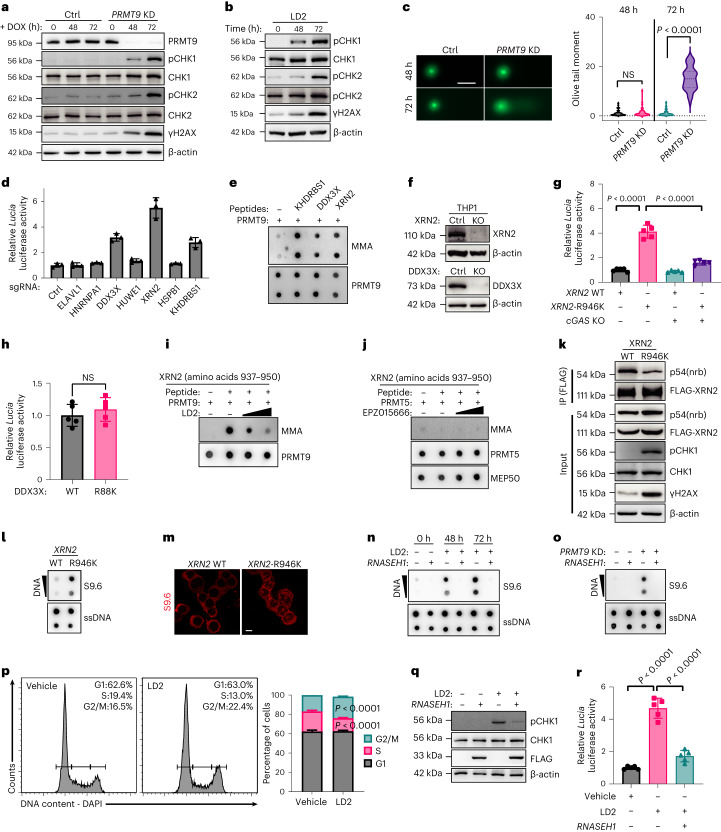
Loss of XRN2 methylation underlies *cGAS* activation. **a**,**b**, Phospho-CHK1, CHK2 and γH2AX levels after *PRMT9* KD (**a**) or LD2 (**b**) in THP1 (*n* = 2 independent experiments). **c**, Comet assay of THP1 after *PRMT9* KD for 48 and 72 h. Right: summary of each group (*n* = 50 cells). Scale bar, 50 μm. The *P* value was determined using an unpaired two-sided *t*-test. **d**, Luciferase activity of THP1-IRF cells after KO of the indicated genes. Data are the mean ± s.d. from three independent experiments. **e**, Methylation assay of KHDRBS1 (amino acids 326–339), XRN2 (amino acids 937–950) or DDX3X (amino acids 80–92) peptides. Methylation was analyzed using an anti-MMA antibody (*n* = 2 independent experiments). **f**, XRN2 and DDX3X levels after respective KO (*n* = 2 independent experiments). **g**, Luciferase activity of WT and *cGAS* KO THP1-IRF cells. gRNA-resistant *XRN2* WT and R946K constructs were ectopically expressed in THP1-IRF cells (*n* = 5 independent experiments). A reporter assay was performed using the cells with KO endogenous XRN2. Data are the mean ± s.d. The *P* value was determined using a one-way ANOVA. **h**, Luciferase activity of THP1-IRF cells (*n* = 5 independent experiments). gRNA-resistant DDX3X WT or R88K constructs were ectopically expressed in THP1-IRF cells. A reporter assay was performed using the cells with KO endogenous DDX3X. Data are the mean ± s.d. The *P* value was determined using an unpaired two-sided *t*-test. **i**,**j**, In vitro methylation of XRN2 peptides with PRMT9 (**i**) or PRMT5 (**j**) with increased dose of LD2 (**i**) or EPZ015666 (**j**) (*n* = 1). **k**, XRN2-engineered THP1 cells were prepared for IP using anti-FLAG beads; interactors were detected as indicated (*n* = 2 independent experiments). **l**,**m**, R-loop signals by dot blots (**l**, *n* = 2) or immunostaining (**m**) in THP1 cells. Scale bar, 10 μm. ssDNA, single-stranded DNA. **n**,**o**, R-loop signals in *RNASEH1*-overexpressed THP1 cells treated with LD2 (2.5 μM) (**n**) or *PRMT9* KD (**o**) (*n* = 2 independent experiments). **p**, Cell cycle of THP1 cells treated for 48 h with LD2 (2.5 μM), *n* = 5 independent experiments. Right: statistics. Data are the mean ± s.d. Right: *P* values were determined using a one-way ANOVA. **q**, Phospho-CHK1 in engineered THP1 cells treated with LD2 (2.5 μM) (*n* = 2 independent experiments). **r**, Luciferase activity of THP1-*Lucia* luciferase cells treated with (2.5 μM) LD2 (*n* = 5 independent experiments). Data are the mean ± s.d. The *P* value was determined using a one-way ANOVA. Source data

PRMT9 inhibition in THP1 cells via LD2 (48 h) or shRNA significantly increased Rad3-related (ATR) signaling, whereas γH2AX elevation and changes in ataxia-telangiectasia-mutated (ATM) signaling were modest (Fig. 7a,b). However, we observed remarkably increased levels of γH2AX and pCHK2 after 72 h of PRMT9 inhibition (Figs. 6h and 7a,b), corresponding with the formation of DNA double strand breaks (Fig. 7c). These findings suggest that PRMT9 inhibition triggers an early insult that selectively activates ATR.

We next asked whether any PRMT9 substrate functions in the DNA damage response and whether its loss underlies ATR activation and cGAS stimulation. SILAC showed that seven of the 23 most downregulated methylated proteins after *PRMT9* KD (Fig. 3c) regulate the DNA damage response (Fig. 3d and Extended Data Fig. 8s). To determine whether KO of any of them phenocopied the PRMT9 inhibition effects, we electroporated THP1-*Lucia* luciferase cells with Cas9 protein and bound guide RNA (gRNA) targeting the respective candidate genes^47^. Of the seven genes, KO of *XRN2*, *DDX3X* or *KHDRBS1* increased reporter activity (Fig. 7d,f); an in vitro methylation assay confirmed PRMT9 catalysis (Fig. 7e). We then ectopically expressed gRNA-resistant full-length XRN2 WT or DDX3X WT complementary DNAs or corresponding methylation-deficient constructs (*XRN2*-R946K or *DDX3X*-R88K) in THP1-*Lucia* luciferase cells and the corresponding *cGAS* KO/THP1-*Lucia* luciferase line, then KO the corresponding endogenous genes. Notably, *XRN2*-R946K expression increased THP1 reporter activity, an effect blocked by *cGAS* deletion (Fig. 7g), while methylation-deficient DDX3X did not increase reporter activity (Fig. 7h). We confirmed that *XRN2*-R946K is methylated by PRMT9, based on in vitro methylation and responses to LD2 treatment (Fig. 7i,j).

We next focused on the exoribonuclease XRN2 whose C terminus interacts with p54nrb to prevent R-loop formation^48^. SILAC analysis revealed that among all XRN2 R residues, only R946 methylation levels were altered by *PRMT9* KD (Supplementary Table 8). To determine if R946 methylation promotes XRN2 recruitment by p54nrb, we performed co-IP analysis. FLAG-tagged XRN2 interaction with p54nrb was decreased in the presence of R946K (Fig. 7k and Extended Data Fig. 8t). Relative to *XRN2* WT, expression of the gRNA-resistant *XRN2*-R946K mutant in THP1 cells engineered to lack endogenous XRN2 promoted R-loop formation (Fig. 7l,m), resulting in ATR activation and γH2AX elevation (Fig. 7k). Consistently, LD2 treatment or *PRMT9* KD promoted excess R-loop formation and activated ATR signaling as early as 48 h after treatment (Fig. 7a,b,n,o). Cell cycle analysis after LD2 treatment showed an increased percentage of G_2_/M (Fig. 7p). Finally, *RNASEH1* overexpression to resolve R-loops (Fig. 7n) decreased ATR/CHK1 activation, partially rescuing the THP1-luciferase reporter activity induced by LD2 (Fig. 7q,r). Collectively, these results indicate that loss of XRN2 methylation contributes to DNA damage by PRMT9 inhibition.

### Combining LD2 with an ICI ablates cancers

**Fig. 8:**
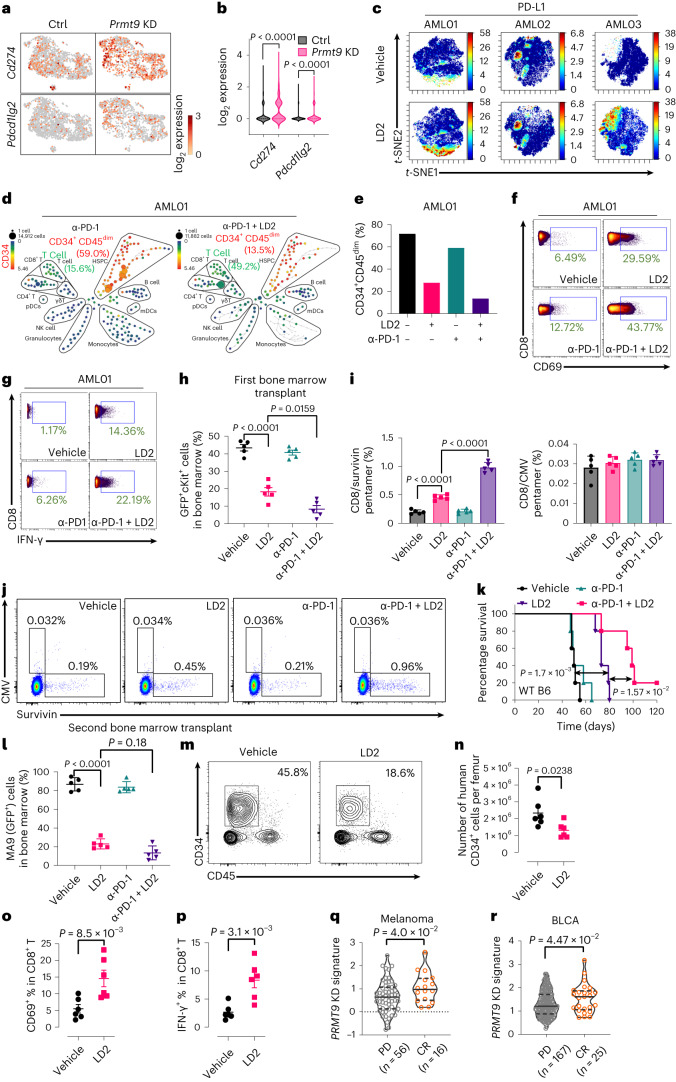
Combining LD2 with an ICI ablates AML. **a**,**b**, Uniform manifold approximation and projection (UMAP) (**a**) and histogram (**b**) showing *Cd274* (*PD-L1*) and *Pdcd1lg2* (*PD-L2*) expression in MA9 cells from the scRNA-seq analysis of Ctrl (*n* = 1,827 cells) and *Prmt9* KD (*n* = 1,124 cells) leukemic cells. Data are the mean ± s.e.m. *P* values were determined using unpaired two-sided *t*-tests. **c**, CyTOF of AML MNCs treated with LD2 (2.5 µM for 4 days), colored according to the expression of PD-L1 based on the CD34^+^CD45^dim^ subsets (*n* = 3 patients). **d**, CyTOF of AML MNCs after treatment. The frequency of CD3^+^ T cells and CD34^+^CD45^dim^ AML blasts were noted. The color bar shows the intensity of CD34 expression. **e**, Relative leukemia cell (CD34^+^CD45^dim^) frequencies of AML01 in **d** and Fig. 4m. **f**,**g**, CD69 (**f**) and IFN-γ (**g**) levels in CD8^+^ T cells in AML01. **h**–**j**, MA9 cells were transplanted (**i**, *n* = 5 mice per group). We treated AML-bearing mice for 3 weeks with vehicle, a PD-1 inhibitor (10 mg kg^−1^ intraperitoneally every other day), LD2 (10 mg kg^−1^ intravenously twice a day) or LD2 plus PD-1 inhibitor. After treatment, leukemic progenitor (GFP^+^cKit^+^) engraftment was assessed (**h**). MA9-specific CD8^+^ T cells were assessed (representative plots are shown in **j**). Cytomegalovirus (CMV)-specific pentamers were the negative control. **i**, Histograms. **j**, Data summary. Data are the mean ± s.e.m. **h**,**i**, *P* values were determined using a one-way ANOVA. **k**, As in **h**, Kaplan–Meier curves show the survival of mice (*n* = 5 mice per group). *P* values were determined using a log-rank (Mantel–Cox) test. **l**, Secondary transplantation (*n* = 5 mice per group) based on bone marrow cells from the first transplants (**h**); MA9 (GFP^+^) cells in the bone marrow were assessed. Data are the mean ± s.e.m. *P* values were determined using a one-way ANOVA. **m**–**p**, Two million AML MNCs were implanted intrafemorally into an irradiated MHC class I and 2 DKO mouse (**n**,**o**,**p**, *n* = 6 mice per group). After engraftment, mice were treated with vehicle or LD2 (10 mg kg^−1^ intravenously twice a day). After 3 weeks of treatment, the number and frequency of leukemic CD34^+^ cells (**m**,**n**) and frequencies of CD8^+^ T cells expressing CD69 (**o**) and IFN-γ (**p**) were assessed. Data are the mean ± s.e.m. **n**,**p**, *P* values were determined using an unpaired two-sided *t*-test. **q**,**r**, *PRMT9* KD gene signature levels in the indicated ICI-treated cohorts of patients enrolled in clinical trials against melanoma (**q**) and BLCA (**r**) cancer with CR and PD^6,50,51^. Single-sample GSEA was applied. Violin plots were used to compare the distribution of NES between groups. Statistical comparisons were carried out using unpaired two-sided *t*-tests. *n*; represents the number of patients. Source data

**Extended Data Fig. 9:**
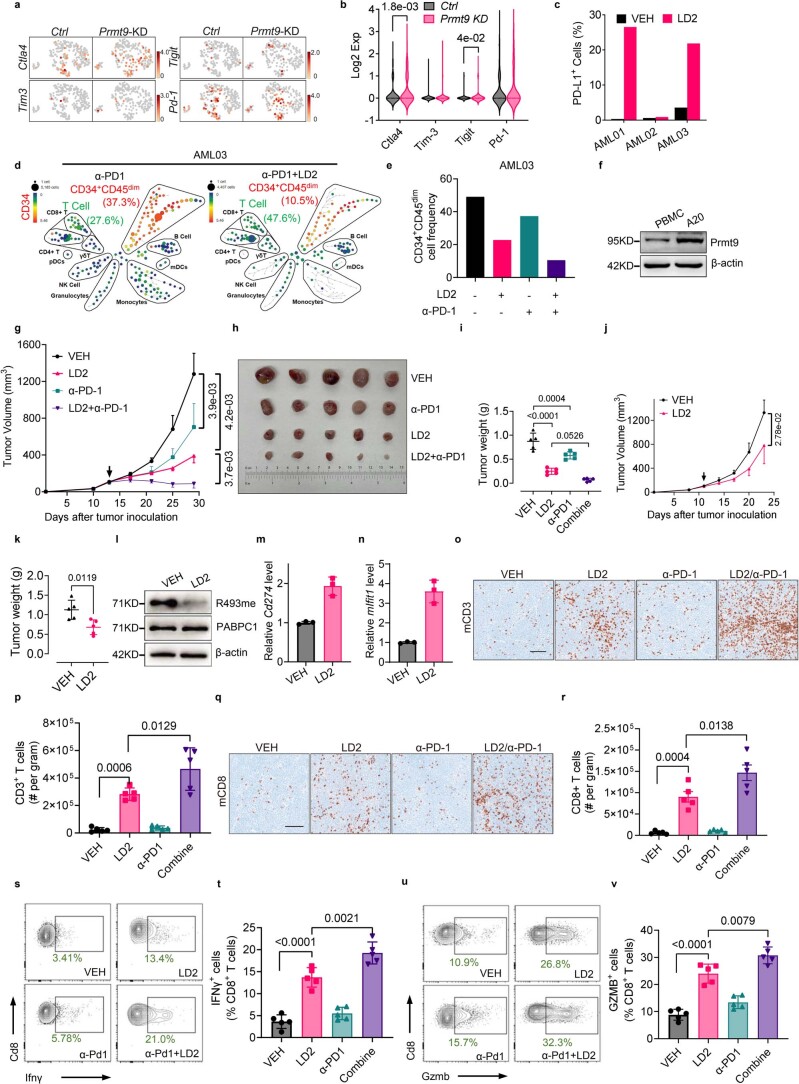
Combining LD2 with an ICI ablates cancers. **a, b**, UMAP (a) and histogram (b) showing indicated gene expression in T cells from scRNA-seq of Ctrl (n = 249 cells) and *Prmt9* KD (n = 231 cells) BM T cells. Data were mean ± SEM. P values were unpaired two-sided t-test. **c**, Frequency of PD-L1+ cells among leukemic subset (CD34+CD45dim) following treatment LD2 (2.5 µM, 4 days), n = 3 samples. **d, e**, CyTOF of AML MNCs after indicated treatment. The frequency of CD3 + T and CD34+CD45dim AML blasts were noted (d). Relative leukemia (CD34+CD45dim) cell frequencies of (d and right panel of Fig. 4m) were shown in (e). **f**, Prmt9 levels in normal mouse PBMCs or A20 (n = 2 independent experiments). **g, h**, Balb/C mice were inoculated with A20 cells (n = 5 mice/group). Tumor bearing mice were treated with isotype control (VEH), anti-PD1 mAb (10 mg/kg/i.p./q.o.d./2 wks), LD2 (100 mg/kg/ i.t./q.d./2 wks) or combination (n = 5 mice/group). Tumor volume was monitored (g) and pictures were acquired (h). **i**, Weight of A20 tumors in each group (n = 5 mice/group); **j, k**, *NSGS* mice (n = 10 mice) were inoculated with A20 cells. Tumor bearing mice were treated with vehicle or LD2 (100 mg/kg/i.t./ q.d./2 wks, n = 5 mice/group). Final tumor weights are shown (k). Data (g, i, j,k) were mean ± SEM. P values were determined by two-way ANOVA (g, j), one-way ANOVA (i) or unpaired two-sided t test (k). **l**, Levels of R493 methylation in A20 cells treated with LD2 (n = 2 independent experiments). **m, n**, Levels of Cd274 (m) and mIfit1 (n) cells in A20 treated with LD2 (n = 3 independent experiments). Data were mean ± SD. **o**, Representative image of anti-mCD3 IHC staining in indicated groups. Scale bar, 100 μm. **p**, Quantification of CD3 T cells, based on the number of cells per gram of tumor in indicated treatment groups (n = 5 mice/group). Data are presented as mean ± SD. P values were determined by one-way ANOVA. **q, r**, Representative image of anti-mCD8. Scale bar, 100 μm. (r) Quantification of CD8 T cells in indicated groups (n = 5 mice/group). **s–v**, Representative plots showing IFNγ (s) and GZMB (u) expression in CD8 + T cells in indicated treatment groups. Quantification of IFNγ + (t) and GZMB + (v) cells among CD8 + T cells in indicated groups (t, v- n = 5 mice/group). Data (r, t, v) are mean ± SEM. P values (r, t, v) were determined by one-way ANOVA. Source data

Based on the scRNA-seq results, among the reported relevant immune checkpoint proteins, *Prmt9* KD significantly upregulated *PD-L1* in cancer cells (Fig. 8a,b), although *PD-L2* and *CTLA-4* were also modestly upregulated (Fig. 8a,b and Extended Data Fig. 9a,b). Notably, in two of three primary AML cocultures, upregulation of *PD-L1* on Prmt9 inhibition was seen (Fig. 8c and Extended Data Fig. 9c).

To determine if a PRMT9 inhibitor synergizes with PD-1 monoclonal antibody (mAb) treatment, we treated AML samples for 4 days ex vivo. The combination elicited T cell expansion and reduced tumor cell frequency (Fig. 8d,e and Extended Data Fig. 9d,e). Activation of T cells was seen among combination-treated cells (Fig. 8f,g).

We next investigated cooperation between PRMT9 inhibitor and αPD-1 treatment using an A20 lymphoma syngeneic model (Extended Data Fig. 9f). Once A20 tumors reached 100 mm^3^, we treated mice with isotype control (vehicle), anti-PD-1 mAb (10 mg kg^−1^ intraperitoneally every other day for 2 weeks), LD2 (100 mg kg^−1^ intratissue injection once a day for 2 weeks) or combined LD2 and anti-PD-1. Tumor volumes were monitored through the end (Extended Data Fig. 9g). A humane endpoint was reached in a vehicle group mouse on day 29. The tumor size of the combined treatment was smaller than that of the vehicle (Ctrl) group starting on day 17. LD2 administration alone significantly decreased tumor size relative to controls after day 21 (Extended Data Fig. 9g–i). We also evaluated LD2 single treatment effects in *NSGS* mice xenografted with A20 cells; treatment modestly decreased A20 tumor growth (Extended Data Fig. 9j,k), probably because of its effects on translation (Extended Data Fig. 9l). Notably, A20 tumor weight in BALB/c mice was reduced in the combination group versus the vehicle controls (Extended Data Fig. 9h,i). LD2 treatment upregulated ISGs and *PD-L1 (Cd274)* (Extended Data Fig. 9m,n). We also observed an increased number of tumor-infiltrating T cells or CD8^+^ T cells after LD2 treatment or combination treatment (Extended Data Fig. 9o–r). Moreover, a remarkable increase in the number of active CD8^+^ T cells was seen in the LD2 single treatment group; the effects were enhanced by the combination treatment (Extended Data Fig. 9s–v).

We next evaluated the combination treatment in an MA9 AML transplant model. We treated AML-bearing mice for 3 weeks with vehicle, anti-PD-1 mAb (10 mg kg^−1^ intraperitoneally every other day), LD2 or LD2 plus anti-PD-1. LD2 was administered at a dose of 10 mg kg^−1^ intravenously twice a day. After treatment, compared to LD2 only, combination treatment significantly decreased leukemia engraftment and expanded tumor-specific T cells (Fig. 8h–j). Notably, combination treatment extended mouse survival and decreased LSC activity (Fig. 8k,l).

**Extended Data Fig. 10:**
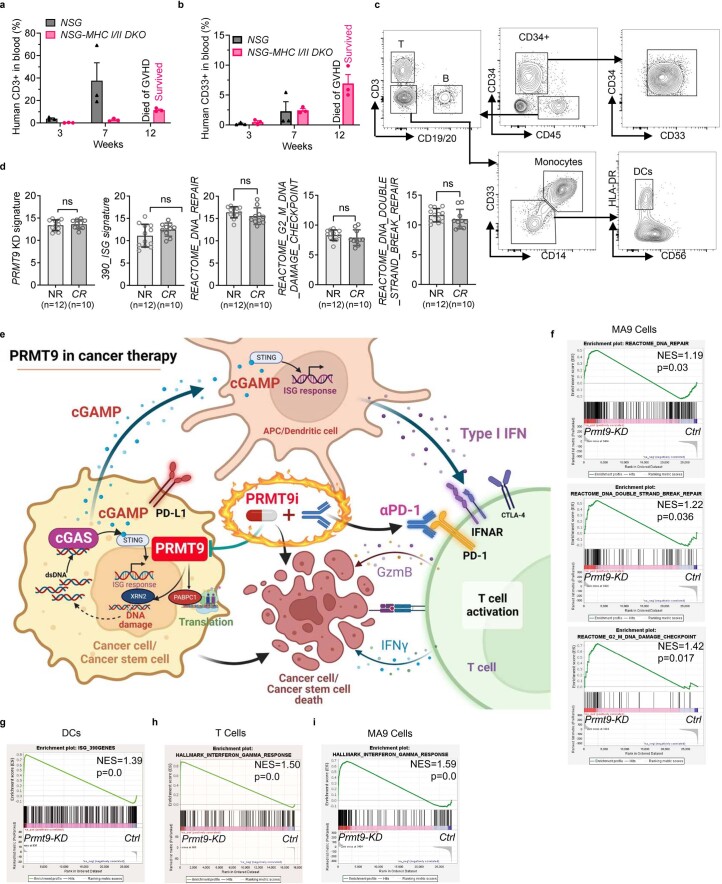
Humanized AML mouse model establishment. **a, b**, Two million AML MNCs were implanted intra-femorally into an irradiated *MHCI/II1/2* double-KO (*DKO*) *NSG* or regular *NSG* mice (n = 3 mice/group). After transplant, engraftment of CD3 + T cells, CD33+ cells in PB were monitored for 12 weeks. Then, BM engraftment of human hematopoietic subsets including T cells, monocytes and DCs, as well as the immature CD33 + CD34+CD45dim subset were assessed. Data were mean ± SEM. **c**, Representative gating of hematopoietic subsets from *DKO* mouse BM. **d**, Single sample gene set enrichment analysis (ssGSEA) of AML trial dataset GSE183415 which contains samples with clinical responses to PD-1 inhibitors (Complete Response [CR, n = 10] vs. No Response [NR, n = 12]) using the following gene signature: *PRMT9* KD signature, 390 ISG signature, Reactome DNA repair signature, Reactome Double strand break repair signature, and Reactome G2M DNA damage checkpoint signature. ‘n’ represents patient numbers in indicated group. Data are presented as mean ± SD, statistical comparisons were performed using unpaired two-sided t-test, ‘ns’ indicates no significance. **e**, Proposed model. **f**, ‘REACTOME’ signature ‘DNA Repair’, ‘DNA Double Strand Break Repair’, ‘G2M DNA Damage Checkpoint’ plots of GSEA of MA9 AML cells (*Prmt9* KD vs. Ctrl). **g**, 390 ISGs genes signature plot of GSEA of BM DCs (*Prmt9* KD vs. Ctrl). **h**, ‘Hallmarks’ signature Interferon Gamma Response plot of GSEA of BM T cells (*Prmt9* KD vs. Ctrl). **i**, ‘Hallmarks’ signature Interferon Gamma Response plot of GSEA of BM MA9 AML cells (*Prmt9* KD vs. Ctrl). For GSEA analysis (g-i), Normalized enrichment scores and family-wise error rate P-value was determined by the GSEA permutation method. The Normalized Enrichment Score is calculated by dividing the Enrichment Score from the actual ranking by the means of the random permutations. An enrichment P-value is calculated by comparing the observed frequency of an annotation term with the frequency expected by chance; individual terms beyond cut-off (p-value ≤ 0.05) are deemed enriched. Source data

We established a humanized AML model. Specifically, in a cohort of MHC class I and II double-KO (DKO) *NSG* mice, we implanted 2 million MNCs from an AML specimen using intrafemoral injection of each DKO mouse. DKO mice showed long-term engraftment of T and CD33^+^ cells (Extended Data Fig. 10a,b), without acute graft-versus-host disease, consistent with other reports^49^. Importantly, we confirmed bone marrow engraftment of human hematopoietic subsets (Extended Data Fig. 10c) and observed selective expansion of the immature CD33^+^CD34^+^CD45^dim^ subset (Extended Data Fig. 10c). We then divided mice into vehicle and LD2 treatment groups. After 3 weeks of treatment, we observed decreased numbers of leukemic CD34^+^ cells (Fig. 8m,n) and increased numbers of active CD8^+^ T cells (Fig. 8o,p) in LD2 relative to the control group.

Also, we assessed the correlation between PRMT9 activity and the response of PD-1 and PD-L1 inhibitors using clinical datasets^6,50,51^. To do that, we defined the *PRMT9* KD gene signature established from RNA-seq analysis of *PRMT9* KD versus Ctrl AML lines (Fig. 6c and Supplementary Table 9). The signature consists of 102 differentially expressed genes (DEGs) common to two AML cell lines (fold change > 2, *P* < 0.05). Notably, higher levels of the *PRMT9* KD gene signature were positively associated with complete response (CR) to ICI versus progressive disease (PD) in two clinical cohorts (Fig. 8q,r). Relevant to AML, we failed to detect any correlation of the signature with clinical responses to PD-1 inhibitors using the only available dataset (Extended Data Fig. 10d).

## Discussion

PRMT9^hi^ LSCs may give rise to immune-evasive leukemia blasts. Our results reveal that targeting PRMT9 not only ablates LSCs but stimulates an anticancer immune response to achieve maximal therapeutic effects. This strategy, when combined with an ICI, could approach a cure. Specifically, our approach targets the arginine methyltransferase PRMT9 to ablate AML LSCs by downregulating the synthesis of short-lived oncoproteins; targeting PRMT9 also induces DNA damage-mediated activation of cGAS and release of cGAMP, thereby cross-priming T cells via a type I IFN response. Moreover, we identified that the lead compound LD2 as a potent inhibitor of PRMT9 activity that promotes robust anti-AML activity (Extended Data Fig. 10e).

PRMT9, one of two SDMA-forming PRMTs, is characterized by a unique duplicated methyltransferase domain^29,52^. In this study, we used a quantitative proteomic method to profile changes in global arginine methylation on PRMT9 knockdown and identified undefined targets. Specifically, methylation at residue R493 enables the PABPC1 protein to bind to the mRNA poly(A) tail, promoting translation. Moreover, XRN2 methylation at R946 may allow complex formation with p54nrb to prevent the DNA double-strand breaks associated with the role of XRN2 in resolving the R-loop (RNA/DNA hybrid) structure^48^. Indeed, PRMT9 inhibition or expression of *XRN2*-R946K in AML cells promoted R-loop formation and ATR signaling, which underlies cGAS activation in cancer cells. Moreover, PRMT9 did not catalyze cGAS methylation (Supplementary Fig. 9).

Our study demonstrates that tumor elimination induced by Prmt9 deletion relies on type I IFN responses. scRNA-seq analysis revealed that the changes in T cell subpopulations seen after *Prmt9* KD are associated with immune memory. Other studies used high-dose cytotoxic chemotherapies that dampen immune responses^53–55^. Interestingly, we found that neither *Prmt9* KO nor LD2 treatment perturbed T cell function.

How does PRMT9 inhibition in cancer cells elicit a distinct response in T cells? In this study, we showed that cGAS-dependent dsDNA sensing by cancer cells is critical for the effects of T cell priming. Notably, leukemia cells express higher levels of cGAS relative to normal counterparts from healthy donors. On *PRMT9* KD, cancer cells accumulate cytosolic dsDNA, providing abundant substrate for cGAS catalysis (Fig. 6i). Such changes in dsDNA are partially due to DNA damage induced by the loss of XRN2 methylation seen after PRMT9 inhibition. Interestingly, GSEA of single-cell transcriptomes from *PRMT9* KD versus control MA9 cells showed significant enrichment of DNA damage response gene signatures (Extended Data Fig. 10f), confirming an association between PRMT9 inhibition and DNA damage.

Moreover, PRMT9 inhibition also downregulated SAMHD1 (Fig. 3g), which antagonizes cGAS–STING activity as reported previously^56^. Consequently, cGAS-activating cancer cells can produce high levels of the immunotransmitter cGAMP. Among all cancers, leukemia cells express the lowest levels of ENPP1, which hydrolyzed cGAMP (Extended Data Fig. 8o,q), allowing sustained cGAMP production in cancer cells. Extracellular cGAMP may be transferred via gap junctions from cancer cells to DCs^57^. Indeed, scRNA-seq analysis of the MA9 model revealed ISG upregulation in DCs (Extended Data Fig. 10g). Moreover, subsequent GSEA analysis showed upregulation of the IFN-α response pathway in T cells from *Prmt9* KD AML bone marrow (Extended Data Fig. 7p). As an outcome of the type I IFN response, T cells (Fig. 5k) exhibited IFN-γ upregulation after *Prmt9* KD. Indeed, we observed significant enrichment of IFN-γ response genes in MA9 cancer cells and in T cells (Extended Data Fig. 10h,i).

Collectively, we showed a biological role for PRMT9 in cancer. We developed a small molecule inhibitor blocking PRMT9 activity. Our study also prompts an appraisal of anticancer drugs with consideration of their impact on immune cells within the tumor microenvironment and provides a rationale for further evaluation of PRMT9 inhibition combined with a PD-1/PD-L1 inhibitor against AML.

## Methods

### Ethics statement

This study follows ethical regulations. Experiments using patient specimens were approved in part by the institutional review boards of City of Hope Comprehensive Cancer Center (COHCCC) and conducted in accordance with the Declaration of Helsinki (2013). Samples were acquired as part of the COHCCC institutional review board-approved clinical protocol no. 18067. All mouse experiments were completed in accordance with the Guidelines for the Care and Use of Laboratory Animals and were approved by the Institutional Animal Care and Use Committee (IACUC) at COHCCC. Experiments were performed in accordance with a protocol approved by the COHCCC ICUC (no. 15046). The maximum tumor size (humane endpoint) permitted by IACUC is 15 mm (diameter). All animals were euthanized before tumor size reached 15 mm in diameter. Maximum tumor size did not exceed 15 mm.

### Patient cells

De-identified, clinically annotated primary patient samples including those derived from peripheral blood or bone marrow were obtained from patients with AML at COHCCC. The annotations are shown in Supplementary Table 1. Normal cells derived from peripheral blood were obtained from the COHCCC. Informed written consent was completed and acquired from all involved participants before sample acquisition. MNC separation, CD34^+^ cell enrichment or CD3^+^ T cell depletion was performed as described previously^58^.

### Cell culture

Molm13 (catalog no. ACC 554, DSMZ), MV4-11 (catalog no. CRL-9591, ATCC), THP1 (catalog no. TIB-202, ATCC), NB4 (catalog no. ACC 207, DSMZ), U937 (catalog no. CRL-1593.2, ATCC), HL-60 (catalog no. CCL-240, ATCC), MA9.6ITD and RAJI (catalog no. ACC 319, DSMZ), UPN1 (catalog no. CVCL_A795, Cellosaurus), BL41 (catalog no. ACC 160, DSMZ), Rec1 (catalog no. ACC 584, DSMZ), OCI-Ly3 (catalog no. ACC 761, DSMZ) and A20 (a gift from Y. Fu) were cultured in Roswell Park Memorial Institute (RPMI) 1640 medium with 10% FCS as described previously^58,59^. All other cell lines, including 293FT (catalog no. R70007, Thermo Fisher Scientific), DMS273 (a gift from R. Salgia), DMS114 (a gift from R. Salgia), SW1573 (a gift from E. Wang), A549 (a gift from E. Wang), SW620 (catalog no. CCL-227, ATCC), HCT116 (catalog no. CCL-247, ATCC), HepG2 (catalog no. HB-8065, ATCC), PC3 (a gift from S. Priceman), DU145 (a gift from S. Priceman), MDA-MB-231 (catalog no. CRM-HTB-26, ATCC), HT1197 (catalog no. CRL-1473, ATCC), A172 (catalog no. CRL-1620, ATCC), MIAPACA2 (catalog no. CRM-CRL-1420, ATCC) and HT1080 (catalog no. CCL-121, ATCC) were cultured in DMEM with 10% FCS. MA9.6ITD cells (MLL-AF9 plus *FLT3*-ITD) were established by J. Mulloy^60^. The human primary normal and AML CD34^+^ cells used for transduction were maintained as described previously^59^. Specifically, as noted in that paper, the medium was StemSpan SFEM (STEMCELL Technologies) supplemented with 50 ng ml^−1^ recombinant human stem cell factor (SCF), 100 ng ml^−1^ Flt3 ligand (Flt3L), 100 ng ml^−1^ thrombopoietin, 25 ng ml^−1^ interleukin-3 (IL-3) and 10 ng ml^−1^ IL-6 (PeproTech). Mouse AML cells were cultured in RPMI 1640 medium with cytokines (mouse IL-3, 10 ng ml^−1^; mouse IL-6, 10 ng ml^−1^; mouse SCF, 30 ng ml^−1^; Supplementary Table 10) as described previously^59^.

### Mice

In all experiments, male and female, 6–10-week-old, WT C57BL/6J (strain no. 000664, The Jackson Laboratory), B6(Cg)-*Rag2*^*tm1.1Cgn*^/J (strain no. 008449, *Rag2*^−*/*−^, The Jackson Laboratory), B6(Cg)-*Ifnar1*^*tm1.2Ees*^/J (strain no. 028288, *Ifnar1*^−*/*−^, The Jackson Laboratory), *Kmt2a*^*tm2(MLLT3)Thr*^/KsyJ (strain no. 009079, MLL-AF9 knock-in, The Jackson Laboratory), B6.129S(C)-*Batf3*^*tm1Kmm/*^*J* (strain no. 013755, *Batf3*^−*/*−^, The Jackson Laboratory), NOD.Cg-*Prkdc*^*scid*^ *Il2rg*^*tm1Wjl*^/SzJ (strain no. 005557, *NSG*, The Jackson Laboratory), NOD.Cg-*Prkdc*^*scid*^ *Il2rg*^*tm1Wjl*^ Tg(CMV-IL3,CSF2,KITLG)1Eav/MloySzJ (strain no. 013062, *NSGS*, The Jackson Laboratory) and NOD.Cg-*Prkdc*^*scid*^ *H2-K1*^*b-*^^*tm1Bpe*^ *H2-Ab1*^*em1Mvw*^ *H2-D1*^*tm1Bpe*^ *Il2rg*^*tm1Wjl*^*/*SzJ (strain no. 025216, *NSG-MHC I/II DKO*, The Jackson Laboratory) mice were used. B6-Ly5.1 (CD45.1, NCI 564) and BALB/c (NCI 028) mice were available from an outside vendor. Male and female mice were housed at the COH Animal Resource Center. All care and experimental procedures followed established institutional guidelines. The mouse room is conditioned with a 14 h light–10 h dark cycle, temperatures of 65–75 °F and 40–60% humidity. The procedure was run in accordance with a protocol approved by the IACUC at COHCCC.

Mouse experiments were performed once: Fig. 2d,e,h (male and female; five WT B6 mice per group); in Fig. 2f,g (male and female; five WT B6 mice per group); Fig. 2q (male and female; eight *NSGS* mice per group); Fig. 2r (male and female; eight *NSGS* mice for Ctrl, seven *NSGS* mice for *Prmt9* KD); Extended Data Fig. 2p (male and female; six *Prmt9*^*loxP*/*loxP*^*/Mx1Cre*^−^ mice for *Prmt9* WT, nine *Prmt9*^l^^oxP/loxP^*/Mx1Cre*^+^mice for *Prmt9* KD); Extended Data Fig. 2q (male and female; eight *Prmt9*^*loxP*/*loxP*^*/Mx1Cre*^−^ mice for *Prmt9* WT, 15 mice (*Prmt9*^*loxP/loxP*^*/Mx1Cre*^+^) for *Prmt9* KD); Extended Data Fig. 2r (male and female; seven B6-Ly5.1 mice per group); Fig. 5d (male and female; seven WT B6 mice per group); Fig. 5e (male and female; five *Rag2*^−*/*−^ mice per group); Fig. 5f(male and female; five *NSGS* mice per group); Fig. 5g (male and female; seven WT B6 mice per group); Fig. 5s (male and female; five WT B6 mice for naive mice, four survival mice from Fig. 5d for survivors); Fig. 5v (male and female; five *Ifnar1*^−*/*−^ mice for *Ifnar1* KO, six WT B6 mice for *Ifnar1* WT); Fig. 6j (male and female; seven WT B6 mice for the *Prmt9* KD group, five WT B6 mice for each of the other three groups); Fig. 6m (male and female; seven WT B6 mice for *cGAS* KO + *cGAS*ΔN group, five WT B6 mice for each of the other two groups); Fig. 6s (seven WT B6 mice for each Batf3 WT group, five *Batf3*^−*/*−^ mice for the *Batf3* KO group); Extended Data Fig. 7c (seven WT B6 mice for the Ctrl and *Prmt9* KD groups, five WT B6 mice for the T and NK cell depletion groups); Extended Data Fig. 9g–i (five *BALB/c* mice per group); and Extended Data Fig. 9j,k (five *NSGS* mice per group). scRNA-seq and bulk RNA-seq were performed once per sample and are shown in Figs. 1e, 5h and 6c. If not otherwise specified, in vitro experiments were repeated at least three times.

### DNA constructs and oligonucleotides

The CD530-EF1A-IRES-GFP vectors were purchased from System Biosciences. The CD530-EF1A-T2A-GFP vectors were modified from CD530-EF1A-IRES-GFP, replacing IRES with T2A sequences. Full-length WT or LDIG-to-AAAA mutant PRMT9 (ref. ^29^) were cloned into CD530-EF1A-IRES-GFP vectors. FLAG-tagged XRN2 and FLAG-tagged DDX3X variants, and FLAG-tagged either full-length WT or C-terminal (amino acids 436–636) PABPC1 or R493K, R481K, R506K or 3RK mutants were cloned into the CD530-EF1A-T2A-GFP vector. All plasmids were synthesized by Genscript. shRNAs targeting human *PRMT9*, mouse *Prmt9*, *PABPC1* and *CREB1* were purchased from Sigma-Aldrich (MISSION shRNA) and cloned into pLKO-SFFV-RFP, as described elsewhere^58^. cGAS WT and the activation mutant ΔN were purchased from Addgene and constructed into a DOX-inducible expression vector. SMARTvectors with shPRMT9 were purchased from Dharmacon (Horizon Discovery). The oligonucleotides used are listed in Supplementary Table 11.

### Compounds

Compounds were sourced from the NCI Developmental Therapeutics Program (DTP), ZINC libraries or MolPort. The PEGylated liposome packaging of LD2 used for animal treatment was prepared using the thin film hydration method. Lipids (distearoylphosphatidylcholine, cholesterol and DSPE-PEG(2000) at a ratio of 3:1:0.2) plus compound were dissolved in chloroform; then, organic solvent was separated in a vacuum to form a thin film. Subsequently, lipids were hydrated in PBS, pH 7.4, at 60 °C to form liposomes.

### Lentiviral transduction

Virus production was as described previously^61^. HEK 293T cells were transfected with pMD2.G and psPAX2 packaging vectors plus lentivectors designed to overexpress or knock down genes using the calcium phosphate method as described previously^61^. Supernatants containing virus particles were filtered and concentrated. Viral infection was performed as described previously^61^.

### qPCR

RNA was prepared according to the TRIzol reagent protocol. After generation of complementary DNA, qPCR with reverse transcription was performed as described previously^59^. The primers used are listed in Supplementary Table 11.

### IP and immunoblotting

Cell lysates were prepared in a buffer containing 50 mM Tris, pH 7.4, 150 mM NaCl and 1 mM EDTA supplemented with protease inhibitors. Cell lysates were incubated with anti-FLAG beads or interested primary antibody (Sigma-Aldrich) overnight and denatured for immunoblotting. Proteins of interest were probed with primary and secondary antibodies. Signals were detected using the SuperSignal West Pico or Femato kits. All immunoblots were imaged using the G:BOX Chemi XX6 gel doc system and quantified with the ImageJ software (NIH).

### ChIP–qPCR

Samples were prepared according to the protocol of the SimpleChIP Plus Enzymatic Chromatin IP Kit (catalog no. 9005, Cell Signaling Technology). Immunoprecipitates were exposed to anti-CREB1 (catalog no. SC-240, Santa Cruz Biotechnology) and anti-H3K27Ac antibodies, plus Protein G magnetic beads. After reversing, DNA was enriched; this was followed by qPCR.

### Flow cytometry

Cells derived from the bone marrow or spleen samples were washed with PBS containing 1% FCS and then passed through a single-cell strainer and subjected to lysis of red cells. Before flow cytometry, cells were stained with the indicated antibodies in the same buffer. Flow cytometry analysis was performed. Data analysis was performed using FlowJo v.10. Molm13 cell engraftment in mice was determined using an anti-human CD45 antibody. CD45.2^+^ donor cells from transplants were determined using anti-mouse CD45.1 and CD45.2 antibodies. Mouse HSPCs were determined by staining with anti-mouse lineage antibody, including cKit, Sca-1, CD16 and CD32, and CD34 antibodies and a lineage antibody cocktail, including anti-mouse CD3, CD4, CD8, CD11b, CD11c, CD19, CD41, Ter119, B220, IgM, NK1.1, Gr-1 and interleukin-7 receptor subunit alpha (IL-7Rα). Anti-mouse Mac1, Gr-1, B220 and Ter119 were used to define mouse bone marrow differentiation. We also detected antigen-specific T cells in tumors as described previously^44^. For intracellular staining, fixed cells were incubated once with antibodies against IFN-γ (clone XMG1.2) and granzyme B (clone QA16A02). To define the human primary samples, we used the following markers: T cells (CD3^+^), B cells (CD19^+^/CD20^+^), monocytes (CD14^+^) and DCs (HLA-DR^+^CD34^−^CD33^−^CD3^−^CD19^−^CD20^−^CD14^−^CD56^−^), as well as the immature CD33^+^CD34^+^CD45^dim^ subset. CD69 and IFN-γ staining was used to determine T cell status. For the cell cycle studies, fixed cells were stained with 4,6-diamidino-2-phenylindole (DAPI).

### Competitive transplantation

Bone marrow cells (0.5 × 10^6^per transplant) from CD45.2^+^ *Prmt9*^loxP^^/*l*^^oxP^*MxCre*^*+*^ or *Prmt9*^loxP/loxP^*MxCre*^−^ mice were combined with CD45.1^+^ bone marrow cells (at 1:1 ratio) and then implanted into lethally irradiated (900 cGy) B6-Ly5.1 mice by intravenous injection. Peripheral blood samples were collected and assessed with CD45.1 and CD45.2 antibodies. Mouse recipients were induced with pIpC (InvivoGen) intraperitoneally 15 mg kg^−1^ every other day for 7 days; CD45.2^+^ chimerism in peripheral blood was assessed every 4 weeks.

### Limiting dilution assays

For the limiting dilution assays, to evaluate LSC frequencies, AML cells were suspended in Colony Forming Cell growth medium with DOX to induce *Prmt9* KD and plated in multi-well plates. To evaluate the frequency of leukemia-initiating cells in vivo, bone marrow cells isolated from Ctrl or *Prmt9* KDMA9 AML mice were injected intravenously into sublethally conditioned recipient mice, as described in Supplementary Table 7. The number of recipient mice with leukemia development was determined in each group. The frequency of LSCs and LICs was determined using the ELDA software.

### AML mouse model and in vivo bioluminescence imaging

To assess the effect of *Prmt9* KO and KD in vivo, MA9 or CMM cells were transduced with lentiviral vectors harboring a luciferase reporter. Cells were used for intravenous inoculation into sublethally irradiated CD45.1 B6 mice or WT B6, *Rag2*^−*/*−^ or *NSGS* mice. As for bioluminescence imaging, mice were administered 150 mg kg^−1^ d-luciferin (GoldBio) within PBS, followed by analysis using Lago X. Bioluminescent signals were quantified using the Aura imaging software (Spectral Instruments Imaging). Total values were determined using the regions of interest and photons s cm^2^ sr. To identify the immune subsets contributing to leukemia regression after *Prmt9* KD, we performed antibody-based depletion with an initial dose of combined anti-CD4 and anti-CD8 treatment or anti-NK1.1 treatment administered 1 day before in vivo DOX administration to *Prmt9* KD mice. Antibodies (400 μg) were injected intraperitoneally twice the first week, and then at 200 μg twice weekly to maintain NK or T cell depletion. To assess DC function in *Prmt9* KD outcomes, we implanted Batf3 WT or *Batf3* KO mice with AML cells for further evaluation.

### Assessment of cell growth, apoptosis and colony formation

Cell growth was assessed using the CellTiter-Glo Assay Kit (Promega Corporation). Apoptosis was determined using annexin V or DAPI. Colony formation capacity was determined as described previously^58,59^.

### SILAC-based quantitative proteomics analysis

#### Proteomics sample preparation

For SILAC, Molm13 cells were cultured in SILAC RPMI 1640 medium (catalog no. 88365, Thermo Fisher Scientific) with 10% FCS (catalog no. A3382001, Thermo Fisher Scientific) and either light l-lysine (catalog no. 89987, Thermo Fisher Scientific) and l-arginine (catalog no. 89989, Thermo Fisher Scientific) for control cells, or heavy lysine (catalog no. 88209, Thermo Fisher Scientific) and l-arginine (catalog no.89990, Thermo Fisher Scientific) for inducible *PRMT9* KD cells, for at least ten passages to ensure full incorporation of light or heavy l-lysine and l-arginine.

After 3 days of DOX induction in both control and *PRMT9* KD cells, light-labeled and heavy-labeled cells were combined at 1:1 ratio. Cells were washed and centrifuged at 300*g* for 5 min. Cell pellets were lysed in 9 M urea with protease and phosphatase inhibitors in HEPES (pH 8.0) buffer. Samples underwent four cycles of sonication for 30 s each using a microtip sonicator (VibraCell VCX130, Sonics & Materials) operating at 50% amplitude. Lysates were centrifuged at 20,000*g* for 15 min; protein quantification was performed by using a bicinchoninic acid (BCA) assay. An equal amount of extracted protein from heavy and light SILAC culture was mixed for further digestion. The sample was first reduced by incubation with dithiothreitol (DTT) (5 mM, 55 °C) and then alkylated by incubation with iodoacetamide (10 mM) in the dark. The sample was diluted fourfold before sequential digestion first with LysC (2 h) and then overnight with Trypsin Gold. Digestion was quenched using trifluoroacetic acid and the sample was desalted using 0.7 ml of a Sep-Pak Classic C18 column (Waters). Eluted peptides were speedvac’d to dryness and reconstituted in 1.4 ml immunoaffinity purification buffer followed by peptide quantification using a BCA assay. We subjected 5% of peptides to global quantitative proteomics analysis and 95% of the rest to methyl-R peptide enrichment. This consisted of sequential incubation of peptides with anti-MMA antibody beads (catalog no. 12235, Cell Signaling Technology) and anti-SDMA antibody beads (catalog no. 13563, Cell Signaling Technology). Enriched peptides were reconstituted in 10 µl loading solvent (98% water, 2% acetonitrile, 0.1% formic acid); 1 µg of nonenriched peptides was used for global protein identification.

#### Results acquisition

Data were obtained on an Orbitrap Fusion Lumos mass spectrometer (methylated peptides) or Orbitrap Eclipse with FAIMS Pro interface (unmodified peptides) coupled to a U3000 RSLCnano LC system with running binary solvent A (0.1% formic acid in water) and solvent B (0.1% formic acid in acetonitrile) at 300 nl min^−1^. Methylated peptides (5 µl per injection) were directly loaded on a 25 cm EasySpray C18 column and eluted over a 120-min gradient as follow: 80 min with 2–19% B, 20 min with 19–30% B, 5 min with 30–98% B, followed by 2 min of high organic wash and return to initial conditions in 1 min. Unmodified peptides (1 µg peptides, 5 µl per injection) were directly loaded on a 50-cm EasySpray C18 column and eluted over 240 min using the following gradient: 12 min with 2–5% B, 158 min with 5–19% B, 40 min with 19–30% B, 9 min with 30–90% B, followed by 4 min of high organic wash and return to initial conditions in 2 min. Using a duty cycle of 3 s (Lumos) or 1 s (Eclipse) per FAIMS CV (−40/−60/−80), most abundant precursors were fragmented using higher-energy collisional dissociation (32% normalized collisional energy on Eclipse and 35% normalized collisional energy on Lumos) and measured in the ion trap. Dynamic exclusion was set to 60 s to prevent resampling of previously analyzed precursors.

#### Proteomics data analysis

MS raw files were searched against the human UniProt protein database (downloaded in 2020, 42,373 entries) and a common contaminant database using MaxQuant v.1.6.17.0. The results were filtered to 1% protein and site false discovery rate (FDR). The resulting methyl peptide SILAC ratios obtained from the MaxQuant evidence.txt output file were normalized to their protein SILAC ratios before further analyses^62^.

### R-methyl analysis

Motif analysis was performed using the iceLogo web application as described previously^30^.

### Polysome profiling

We performed polysome profiling as described previously^28^. Engineered Molm13 cells were DOX-induced for 3 days to delete PRMT9 expression and then treated for 5 min with 100 μg ml^−1^ cycloheximide. After treatment, cells were collected and lysed. We prepared sucrose density gradients (15–45% w/v) using a Gradient Master (BioComp Instruments). Then, the supernatant from the cell lysates was separated using centrifugation and fractionation. The collected RNA was further assessed in the qPCR analysis.

### OPP protein synthesis assay

Protein synthesis was assessed by using the Click-iT Plus OPP Assay Kit (Thermo Fisher Scientific), with modifications. Briefly, treated cells were exposed to Click-iT OPP, then washed with PBS and fixed. After permeabilization for 15 min, cells were reacted with cocktail, then analyzed using flow cytometry.

### In vitro methylation assay

The assay was performed in a 30-µl reaction with 50 mM Tris HCl, pH 7.4, 50 mM NaCl, 50 mM KCl, 1 mM MgCl_2_ and 1 mM DTT buffer. Specifically, 1 µg purified PABPC1-CT protein or synthesized peptides, 1 µg purified PRMT9 protein and 5 µM of SAM (Cayman Chemical) were combined. Methylated proteins and peptides were detected with immunoblot or dot blot assays using anti-pan-SDMA, anti-pan-MMA, anti-pan-ADMA or our in-house PABPC1 R493me antibody. The R493me antibody was created by Genemed Synthesis. For the ex vivo tritium labeling of the methylation assay, 1 µg purified PRMT9 protein, 1 µg HA-tagged PABPC1 WT or corresponding PABPC1-R481K/R493K/R506K (3RK) protein, which were immunoprecipitated from 293T cells, and 1 µl *S*-adenosyl-l-[methyl-3H] methionine (78 Ci mmol^−1^) was added to a 30 µl reaction mixture at 30 °C for 1 h. Samples were separated and transferred to polyvinylidene membranes for further assessment.

### PRMT9 structure-based virtual screening

The crystal structure of human PRMT9 (Protein Data Bank (PDB) ID 6PDM; 2.45 A resolution) was used for virtual screening. Missing loops were added using a molecular operating environment loop modeler. A box size of 25 × 21 × 27 Å^3^ centered around the cocrystalized chemical probe was used for screening, which includes both the SAM pocket and catalytic pocket in the N-terminal methyltransferase domain (amino acids 150–520). To rank the binding affinity, parallel AutoDock Vina^63,64^ runs were conducted on a local computer cluster. Seven hundred thousand compounds from the ZINC library were selected using the following criteria: molecular weight 350–450, log *P* < 3, total charge −2e to +2e and availability. In addition, we also screened the NCI library (NCI DTP 260,000 compounds). Each ligand was docked ten times and ranked according to the lowest binding energy score. After screening, we purchased the top 300 candidates (142 of them were available) from the NCI DTP and the top 100 candidates (70 of them were available) from the ZINC library to assess anti-AML activity. To estimate lead compound selectivity, we also performed Vina docking of LD2 into human CARM1 (PDB ID 5U4X), PRMT5 (PDB ID 4X61), PRMT7 (PDB ID 4M38) and PRMT9. To compare LD2 binding to PRMT5 versus PRMT9, we carried out two replicas of 100-ns molecular dynamics simulation of LD2 docked into each.

### Saturation transfer difference and Carr–Purcell–Meiboom–Gill NMR assays

Maltose binding protein (MBP)-tagged PRMT9 core methyltransferase domain (150–474) protein was expressed and purified by Genscript. Briefly, the PRMT9 core methyltransferase domain sequence was inserted into the pMAL-c5X vector between the Nde I and EcoR I sites. Tagged protein was expressed in BL21 and purified on an MBP column, followed by Superdex 200 and Q Sepharose columns. Proteins were sterile-filtered and lyophilized after extensive dialysis against the NMR buffer (50 mM NaH_2_PO_4_, pH 7.5). Deuterium oxide-based sodium phosphate buffer was used with 5% DMSO-d_5_. For the STD NMR assay, the molar ratio of LD2 to PRMT9 was 60:1 in which the concentration of PRMT9 was 0.67 μM; 50 μM trimethylsilylpropanoic acid-d_4_ was used as the internal reference. The molar ratios between PMRT9 and LD2 were 1:20, 1:40 and 1:60, in addition to a control sample with free LD2. LD2 concentration in the Carr–Purcell–Meiboom–Gill (CPMG) experiments was 40 µM. The NMR saturation transfer difference (STD) experiments were carried out at 25 °C on a 700-MHz Bruker Ascend system equipped with a 5-mm triple resonance cryogenic probe as described previously^65^. The CPMG experiment was performed as described previously^66^. Data were analyzed using Bruker TopSpin v.3.6.

### Thermal shift assay

We also assessed whether LD2 binds to PRMT9 directly in vivo; to do so, a cellular thermal shift assay was performed as described previously^39,40^. We first engineered Molm13 cells to overexpress FLAG-tagged PRMT9 WT or PRMT9 mutant (W152A, D258A and E433A; all three residues are predicted drug and PRMT9 binding sites). Five million cells were pretreated with 2.5 µM LD2 overnight. DMSO was used as the control. Cells were aliquoted in each tube and heat-shocked using Thermal Cycler at the indicated temperatures. Cells were then lysed for the immunoblot assay. Experiments were performed using three biological replicates.

### Primary AML MNC culture, mass cytometry staining, acquisition and analysis

Two million MNCs from AML bone marrow specimens were cultured per well in 24-well plates in IMDM plus 20% FCS under physiological cytokine conditions as described previously^41,42^ (granulocyte-macrophage colony-stimulating factor in 200 pg ml^−1^, granulocyte colony-stimulating factor in 1 ng ml^−1^, SCF in 200 pg ml^−1^, IL-6 in 1 ng ml^−1^, macrophage inflammatory protein-1 alpha in 200 pg ml^−1^ and leukemia inhibitory factor in 50 pg ml^−1^). We then used the EasySep Dead Cell Removal Kit (STEMCELL Technologies) to ensure more than 95% living cells before culture. Cells were treated with vehicle (dimethylsulfoxide), 2.5 μM LD2, anti-PD-1 (pembrolizumab, 10 µg ml^−1^, SIM0010, Bio X Cell) or LD2 plus anti-PD-1 for 4 days at 37 °C. On day 4, cells were pretreated for 6 h with brefeldin A and subjected to CyTOF immunostaining with customized surface or intracellular marker antibodies, according to Fluidigm CyTOF protocols (PN400279A4). An untreated peripheral blood mononuclear cell sample from a healthy donor served as a control for phenotyping. Samples were acquired on a Fluidigm Helios. Data were normalized and saved as FCS files before analysis using the Cytobank software (https://premium.cytobank.org/). After data were cleaned up, spanning-tree progression analysis for density-normalized events was used to cluster AML cells and immune cell subpopulations based on the median level of each.

### In silico analysis of CTL levels in primary AML samples

For *CD8A*, *CD8B*, *GZMA*, *GZMB* and *PRF1*, the average expression levels of these genes were used to estimate CTL levels in AML samples^43,67^. We carried out in silico tests to calculate the ratio of PRMT9^hi^ and PRMT9^lo^ patients exhibiting high versus low CTL scores using both GSE144688, which includes 526 samples of patients with AML, and GSE12417, which includes 163 patient samples. For each patient, high versus low CTL scores were decided according to cutoff of 0.5 for the *z*-score. A Fisher’s exact test was used to assess significance.

### scRNA-seq

#### Library preparation

Bone marrow cells in MA9-transplanted mice, and bone marrow and spleen cells in Ctrl and *Prmt9* KD mice administered DOX in drinking water over 7 days, were collected for analysis. Single cells were resuspended in 0.4% BSA and loaded to generate an emulsion of single-cell gel beads. Approximately 5,000–10,000 cells were loaded per channel. Libraries were prepared using the Single Cell 3′ Library & Gel Bead, Single Cell 3′ Chip and i7 Multiplex Kits, according to the Single Cell 3′ Reagent Kits v2 User Guide (part no. CG00052 Rev A). Libraries were sequenced on an Illumina HiSeq 4000 system.

#### Data processing

We used the Cell Ranger Single Cell Software Suite to perform single-cell 3′ gene counting and aggregation of multiple samples to generate raw counts, cell barcodes and gene features. The R package Seurat was run as the platform to implement all data processing procedures^68^.

#### Quality control, normalization and batch removal

Cell quality control was executed as follows: the minimum detected genes (3) in each cell; the minimum number of cells (200) related to each gene; and the maximum fraction (0.2%) of counts from mitochondrial genes per cell barcode. The high-count depth threshold (2,000) was used to filter out potential doublets. Then, the count matrix was normalized to obtain the correct relative gene expression abundance between cells^69^. Then, the R package Harmony was applied to remove batch effects due to biological differences between cell types or states.

#### Feature selection, dimension reduction and visualization

To retain informative genes with high variability, genes with small variations (below 2) among all cells were filtered out. Then, the dimensions of count matrices were reduced using dedicated dimension reduction algorithms, such as UMAP and *t*-distributed stochastic neighbor embedding (*t*-SNE). Two-dimensional visualization outputs were then generated using the leading reduced components in the UMAP and *t*-SNE plots.

#### Clustering and annotation

UMAP-related processed data were regarded as the input of cell clustering. Neighborhood distances among all cells were determined to infer the identity of each cell. Then, clusters were acquired via specified distance metrics (Euclidean distance). Furthermore, for each cluster, the R package MAST was used to deduce significant DEGs. These DEGs were considered markers of a cluster and were used for annotation purposes. Annotations were conducted manually by comparing marker genes with the literature and arranging cell categories. In addition, automatic annotation of cell clusters was done using the R package SingleR, as described previously^70^. By combining both annotation styles, the final cell type labels of each cluster were acquired.

#### GSEA (for T cell and MA9 clusters)

For the cell type clusters of interest, GSEA was performed based on preordered genes ranked using MAST-derived (−log_10_(*P*_adj_) × sign (log fold change)) with 1,000 permutations^71^. The gene sets of the Hallmark, Kyoto Encyclopedia of Genes and Genomes, chemical genetic perturbation and Gene Ontology-Biological Process categories of the Molecular Signatures Database were considered as the signatures. Finally, specific enriched genes within a cluster were visualized by averaging their expression among all cells in that cluster. Key enriched gene expression was rescaled by *z*-scores and visualized in the heatmap.

#### T cell subset identification

scRNA-seq uncovered ten distinct T cell clusters (c0–c9). c0 cells expressed *Cd4* and *CD62L*, but not the effector and memory T cell marker *Cd44* or T cell activation genes. Thus, c0 was defined as naive CD4^+^ T cells. Similarly, c1 cells expressed *Cd8a* and *CD62L* but not *Cd44* or other T cell activation markers and were defined as naive CD8^+^ T cells. c2 cells expressed *Cd8a*, *Cd44* and *Sell,* and intermediate levels of *Tbx21* (T-bet) and *Eomes*, and represented a memory CD8^+^ T cell population. c3 cells expressed high *Cd4*, *Cd44* and *Icos*, *Ctla4, Tnfrsf4* and *Pdcd1*, but did not express *CD62L* and were defined as activated and effector CD4^+^ T cells. c5 cells expressed *Cd44* and showed the highest levels of *Ifng*, *Gzmb*, *Icos*, *Tim-3*, *Il2ra*, *Tnfrsf18* and *Lag3*, considered as differentiated CTLs. c6 cells were defined as T_reg_ cells because they express *Cd4*, *Il2ra (Cd25)* and *Foxp3*. c4, c7, c8 and c9 cells contained both CD4^+^ and CD8^+^ T cells. c4 and c9 showed lower levels of activation markers, and lower *CD62L* and higher *Cd44*, suggesting that they represent T_eff_ cell populations. c7 expressed only the naive T cell marker *CD62L*, indicating a naive population, while c8 expressed lower *CD62L* and higher *Cd44*, but did not express other T cell activation markers, suggesting it represents a memory T cell population.

### Bulk RNA-seq analysis

Total RNA was prepared using the TRIzol reagent (Thermo Fisher Scientific). RNA quality (RNA integrity number) was assessed and sequenced on an Illumina HiSeq 2500 system. RNA-seq reads were aligned with default settings. Count data were normalized. Genes were defined as differentially expressed if the fold change was less than 1.5 or less than 0.67, with an FDR less than 0.05, and at least one sample showing reads per kilobase per million mapped reads greater than 1. We performed hierarchical clustering of DEGs using Cluster v.3.0 with Pearson correlation distance and average linkage, and visualized them with Java TreeView. Enrichment analysis on the pathways of Hallmark, Kyoto Encyclopedia of Genes and Genomes and chemical genetic perturbation was performed using GSEA.

### Detection of cGAMP

cGAMP levels were detected as reported elsewhere^72,73^. THP1 cells were DOX-treated to induce *PRMT9* KD for 2 days; serum-free Phenol Red RPMI (Thermo Fisher Scientific) medium was replaced for another 24 h. Conditioned medium was collected and cGAMP levels were detected using the Enzyme Immunoassay Kit (Arbo Assays). To determine cGAMP levels in the bone marrow microenvironment of control and *Prmt9* KD mice, bone marrow fluid was collected by centrifuging tibias and femurs at 8,000 rpm for 15 s; then, cGAMP levels were assessed.

### *Lucia* luciferase reporter assay

WT (catalog no. thpd-nfis, InvivoGen), *cGAS* KO (catalog no. thpd-kocgas, InvivoGen) and *MAVS* KO (catalog no. thpd-komavs, InvivoGen) THP1-Dual cells were used for the reporter assay. The purchased THP1-Dual cells (InvivoGen) were derived from the human THP1 monocyte line harboring the *Lucia* gene. Reporter cells were further engineered with inducible *PRMT9* shRNA or control shRNA. After DOX treatment to *PRMT9* KD or LD2 to inhibit PRMT9 in these cells, *Lucia* luciferase activity was determined as described by the manufacturer (InvivoGen) by adding QUANTI-Luc reagents and read with a FilterMax F5 microplate reader (Molecular Devices).

### Immunofluorescence microscopy

Cells were spun onto glass coverslips, fixed and incubated with primary anti-dsDNA (AE-2), γH2AX or S9.6 antibodies, then with secondary antibody. Slides were then mounted in 90% glycerol solution containing DAPI (Thermo Fisher Scientific) and examined under a ZEISS LSM 880 confocal microscope.

### Comet assays

We used the OxiSelect Comet Assay Kit (Cell Biolabs). Briefly, after *PRMT9* KD, THP1 cells were mixed with prewarmed (37 °C) Comet agarose at a 1:10 ratio (v/v), then loaded onto the top of the Comet agarose base layer. Slides were immersed for 60 min in lysis buffer at 4 °C, which was washed with prechilled alkaline solution. After three washes with prechilled Tris/Borate/EDTA buffer, slides were subjected to electrophoresis at 1 V cm^−1^ for 15 min, and then rinsed twice with deionized water. Comets were examined under a widefield ZEISS Axio Observer 7 fluorescence microscope. Approximately 50 cells were determined using the OpenComet software in Image J and shown as olive tail moments^74,75^.

### Gene editing in THP1 cells

THP1 reporter cells were electroporated with ribonucleoprotein complexes consisting of Cas9 protein and sgRNAs in the Neon Transfection System; 20 μmol l^−1^ guide RNA (gRNA) (as listed in Supplementary Table 11) were mixed at a 1:1 ratio. KO efficiency was assessed using immunoblot analysis.

### Cocultures of bone marrow DCs and T cells

As described previously^57^, bone marrow cells were cultured with complete RPMI medium containing 20 ng ml^−1^ granulocyte-macrophage colony-stimulating factor (PeproTech). Fresh medium was added on days 3 and 6. CD8^+^ T cells were isolated from the spleens of OT-1 transgenic mice. MA9-OVA cells were pretreated for 2 days with LD2 and then cocultured overnight with collected bone marrow-derived DCs. Supernatants were collected for IFN-β assessment. Bone marrow-derived DCs were selected using a CD11c^+^ selection kit (STEMCELL Technologies) and cocultured for 48 h with OT-1 CD8^+^ T cells. IFN-γ supernatants were assayed using a mouse IFN-γ Flex Set Cytometric Bead Array.

### MA9 AML model in vivo treatment and assessment of leukemia-specific immunity

Once leukemia cells were engrafted, MA9 syngeneic transplant mice were treated for 3 weeks with vehicle control, LD2, single anti-PD-1 mAb (catalog no. BE0146, Bio X Cell, 10 mg kg^−1^ intraperitoneally every other day) or LD2 plus anti-PD-1 antibody. LD2 was administered at 10 mg kg^−1^ intravenously twice a day, based on the preliminary pharmacokinetic and pharmacodynamic results. Mice were assessed for overall survival or killed directly to assess MA9 cell engraftment in bone marrow and perform staining with survivin-specific pentamers to assess MA9-specific immunity as described elsewhere^44^. Briefly, the bone marrow of MA9 mice was stained with anti-CD8 together with survivin-specific pentamers. CMV-specific pentamers were the negative controls. The percentage of survivin or CMV pentamer-positive CD8 T cells was assessed using flow cytometry. Secondary transplantations were performed to evaluate LSC activity in each group by assessing MA9 cell engraftment in the bone marrow.

### Humanized model

The model was established using MHC class I and II DKO *NSG* mice^49^. To do so, we implanted 2 million MNCs from AML specimens intrafemorally into an irradiated DKO *NSG* mouse. After transplantation, MHC-deficient mice showed long-term (approximately 12 weeks in peripheral blood) engraftment of T and CD33^+^ cells without developing acute graft-versus-host disease. A panel of human lineage and progenitor cell markers (CD45, CD33, CD34, CD14, CD19, CD20, CD3, CD56, HLA-DR) was used to define T cells, B cells, monocytes, DCs and immature CD33^+^CD34^+^CD45^dim^ cells. Mice were divided into two groups and treated with vehicle or LD2. Three weeks later, the number and frequency of leukemic CD34^+^ cells and the number of CD8^+^ T cells expressing CD69 and IFN-γ were assessed.

### Non-Hodgkin lymphoma tumor models

A20 cells (3 × 10^6^) were subcutaneously implanted into syngeneic BALB/c mice. When tumor volume reached 100 mm^3^, mice were randomized into treatment groups. Tumor-bearing mice were treated with isotype control (vehicle), anti-PD-1 mAb (10 mg kg^−1^ intraperitoneally every other day for 2 weeks), LD2 (100 mg kg^−1^ intratissue injection daily for 2 weeks) or a combination of LD2 with anti-PD-1. Tumor volume was monitored through the end of the study when a humane endpoint was reached. The maximum tumor size (humane endpoint) permitted by the IACUC is 15 mm (diameter). All animals were euthanized before tumor size reached 15 mm in diameter. The microenvironmental components of tumors were analyzed using immunohistochemistry (IHC) and intracellular staining followed by flow cytometry.

### IHC

Fixed A20 tumors were embedded in paraffin. Four-micrometer-thick sections on slides were incubated for 1 h at 60 °C, deparaffinized and then rehydrated before IHC staining. Slides were blocked with 3% H_2_O_2_. Slides were subjected to antigen retrieval for 15 min at 120 °C in citrate buffer, treated with Tris-buffered saline and incubated for 1 h with anti-mouse CD3 or anti-mouse CD8 antibody. After washing, slides were incubated with secondary antibody. Slides were developed and counterstained with Mayer’s hematoxylin solution. Slides were scanned using whole slide imaging and analyzed using the NDP.view2 software (Hamamatsu).

### Analysis of tumor-infiltrating cells

Portions of fresh A20 tumors were cut into small pieces, then dissociated with type IV collagenase, type IV DNase and type V hyaluronidase at 37 °C for 30 min. Cell suspensions were passed through a 70-μm strainer and centrifuged at 300*g* for 5 min. Cells were stained for 30 min using a Live-or-Dye Fixable Viability Stain Kit (catalog no. 32018, Biotium). Next, cells were stained with immune cell surface markers (mouse CD45-allophycocyanin, mouse CD3-allophycocyanin/cyanine 7, mouse CD4-Alexa Fluor 700 and mouse CD8-Brilliant Violet 605). After two washes, cells were fixed and permeabilized, then intracellularly stained with mouse IFN-γ-phycoerythrin and granzyme B-fluorescein isothiocyanate antibodies in the permeabilization for the flow analysis. Results were analyzed with FlowJo v.10 (FlowJo LLC).

### Statistics and reproducibility

Studies involving independent cohorts of mice were typically performed once, with several exceptions stated in the figure legends. No specific statistical tests were applied to determine sample size; size was established according to our previous experience with the models used. Accordingly, we typically used experimental cohorts of 5–7 mice. The experiments were not randomized. Investigators were not blinded to allocation during the experiments and outcome assessments. Data collection and analysis by all investigators were not performed blinded to the conditions of the experiments. No data were excluded from the analyses.

In general, data from independent experiments are shown as the mean ± s.d. or s.e.m. Statistics were determined using an unpaired, two-tailed Student’s *t*-test, a two-way ANOVA, a one-way ANOVA and a two-sided Fisher’s exact test. Survival results were analyzed with a log-rank (Mantel–Cox) test and expressed as Kaplan–Meier survival curves. Prism (GraphPad Software) was used for the statistics; the detailed methods are described in each individual figure legend.

### Reporting summary

Further information on research design is available in the Nature Portfolio Reporting Summary linked to this article.

### Supplementary information

Supplementary Information Supplementary Figs. 1–9 and Tables 1–11.

Reporting Summary

Supplementary Tables Supplementary Tables 1–11.

Supplementary Data 1 Numerical source data for Supplementary Figs. 1c,d and 8b.

### Source data

Source Data Fig. 1 Unprocessed immunoblots.

Source Data Fig. 1 Statistical source data.

Source Data Fig. 2 Unprocessed immunoblots.

Source Data Fig. 2 Statistical source data.

Source Data Fig. 3 Unprocessed immunoblots.

Source Data Fig. 3 Statistical source data.

Source Data Fig. 4 Unprocessed immunoblots.

Source Data Fig. 4 Statistical source data.

Source Data Fig. 5 Statistical source data.

Source Data Fig. 6 Unprocessed immunoblots.

Source Data Fig. 6 Statistical source data.

Source Data Fig. 7 Unprocessed immunoblots.

Source Data Fig. 7 Statistical source data.

Source Data Fig. 8 Statistical source data.

Source Data Extended Data Fig. 1 Unprocessed immunoblots.

Source Data Extended Data Fig. 1 Statistical source data.

Source Data Extended Data Fig. 2 Unprocessed immunoblots.

Source Data Extended Data Fig. 2 Statistical source data.

Source Data Extended Data Fig. 3 Unprocessed immunoblots.

Source Data Extended Data Fig. 3 Statistical source data.

Source Data Extended Data Fig. 4 Unprocessed immunoblots.

Source Data Extended Data Fig. 4 Statistical source data.

Source Data Extended Data Fig. 5 Unprocessed immunoblots.

Source Data Extended Data Fig. 6 Unprocessed immunoblots.

Source Data Extended Data Fig. 6 Statistical source data.

Source Data Extended Data Fig. 7 Statistical source data.

Source Data Extended Data Fig. 8 Unprocessed immunoblots.

Source Data Extended Data Fig. 8 Statistical source data.

Source Data Extended Data Fig. 9 Unprocessed immunoblots.

Source Data Extended Data Fig. 9 Statistical source data.

Source Data Extended Data Fig. 10 Statistical source data.

## Data Availability

The scRNA-seq and bulk RNA-seq data that support the findings of this study have been deposited in the Gene Expression Omnibus under accession nos. GSE217195 and GSE217396. The PRMT9 SILAC proteomics data have been deposited in PRIDE under accession no. PXD039441. The human cancer PRMT9 expression data were derived from the TCGA Research Network (http://cancergenome.nih.gov/). The dataset derived from this resource that supports the findings of this study is available in the Source data and Supplementary information. A previously published reference dataset of bone marrow samples from newly diagnosed patients with AML and healthy age-matched controls, the BEAT AML dataset, can be found at http://vizome.org/aml/. The TARGET-AML data can be found under accessions nos. GSE14468, GSE12417, GSE63270 and GSE183415. Other published datasets and information are also available at https://DepMap.org/portal/, https://www.cbioportal.org/ (TCGA PanCancer Atlas Studies), https://www.fobinf.com/ (BloodSpot) and GEPIA http://gepia.cancer-pku.cn/ (GEPIA). Source data for all the main figures, extended data figures and supplementary information have been provided as source data files or supplementary files. All other data supporting the findings of this study are available from the corresponding author upon reasonable request. Source data are provided with this paper.

## Code availability

No custom code was created. All the packages used in this study are open-source R packages. Briefly, Seurat v.5.0.0 was used for scRNA-seq data quality control, normalization, feature selection, dimension reduction and visualization. Harmony v.1.1.0 was used for batch removal between different scRNA-seq samples. MAST v.1.26.0 was used for the differential gene expression analysis between scRNA-seq UMAP clusters. SingleR v.2.2.0 was used for the scRNA-seq cell type annotations. edgeR v.3.42.4 was used for differential gene expression analyses between bulk RNA-seq samples. In addition, GSEA v.4.0.3 was implemented for all the pathway enrichment work.
