# Supplementary material for: Targeting PRMT9-mediated arginine methylation suppresses cancer stem cell maintenance and elicits cGAS-mediated anticancer immunity
Source: Nat Cancer. 2024 Feb 27;5(4):601–24. doi: 10.1038/s43018-024-00736-x (PMC11056319; doi:10.1038/s43018-024-00736-x)

nature cancer

Article https://doi.org/10.1038/s43018-024-00736-x

Targeting PRMT9-mediated arginine methylation suppresses cancer stem cell maintenance and elicits cGAS-mediated anticancer immunity

In the format provided by the authors and unedited

Supplementary Fig.1

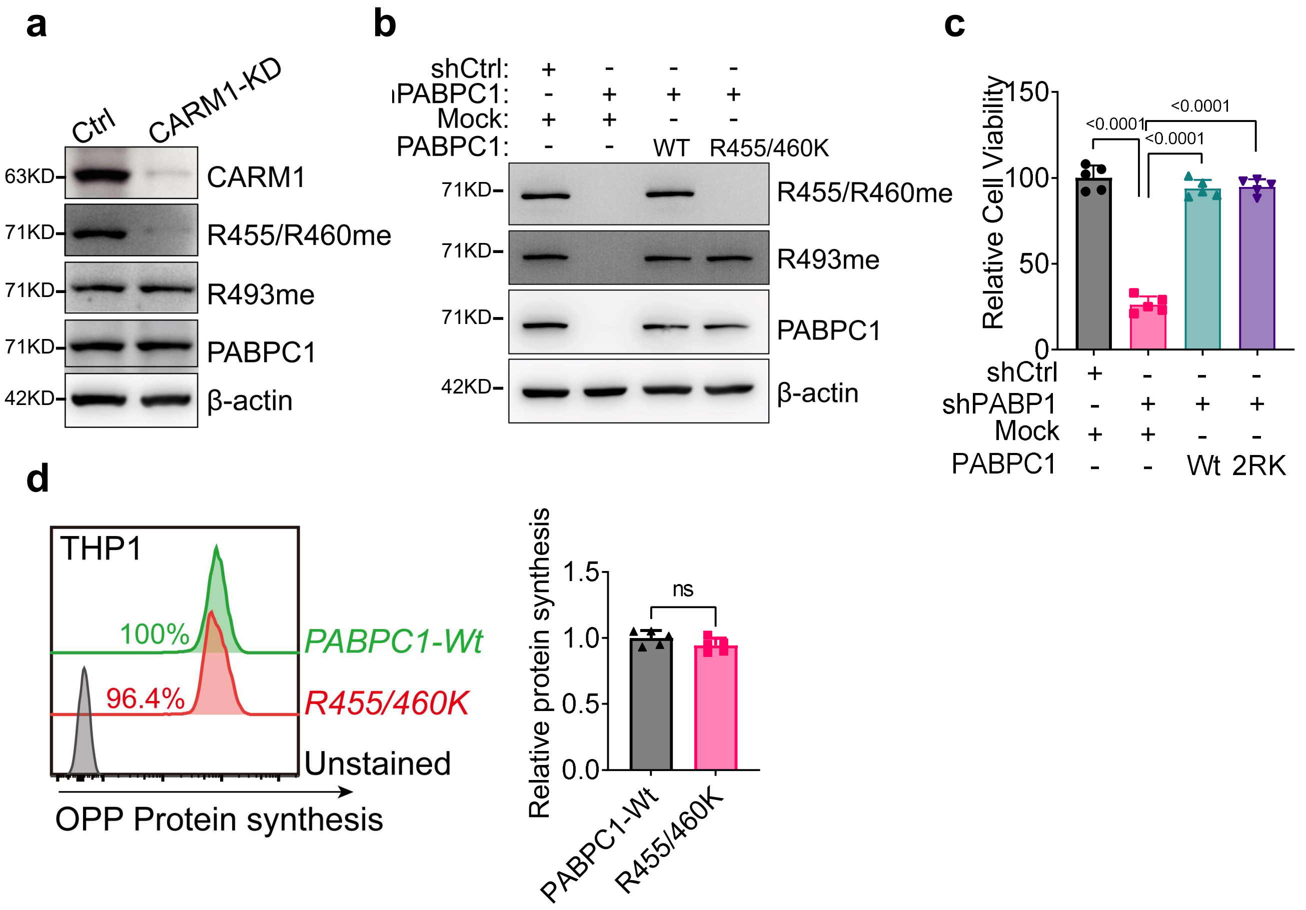

Supplementary Fig.1 CARM1 does not catalyze PABPC1 methylation at R493.

(a) Protein levels of PABPC1 R455/460, R493 methylation in Molm13 cells after CARM1 KD (n=2). (b-c) Molm13 cells were transduced with Mock or PABPC1 (WT or R455/460K) expression vectors resistant to PABPC1 shRNA, and PABPC1 expression was assessed by western blot after the cells with endogenous PABPC1 KD (b). Cell viability was assessed by an MTS assay (c), n=5 independent experiments . Data are presented as mean ± SD. P values each comparison was determined by one-way ANOVA. (d) Protein synthesis of PABPC1-WT or R455/460K mutant Molm13 cells were assessed by an OP-Puro assay. Bar chart (right) summarizes results in PABPC1-WT vs. R455/460K group. Data are presented as mean ± SD. P value was determined by unpaired two-sided t test.

Supplementary Fig.2

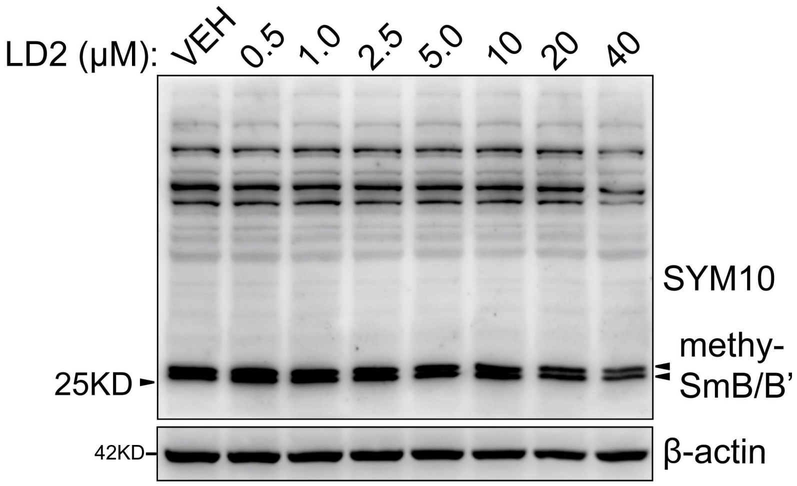

Supplementary Fig.2 LD2 at a low concentration does not inhibit PRMT5.

Dose-dependent inhibition of cellular SmB/B' (PRMT5 substrate) methylation level after 2 days of treatment with LD2 in Molm13 cells (n=1). Cells were treated with a dose titration of 0.5–40 μM LD2 for 48 hr. The inhibition activity of LD2 to PRMT5 was evaluated by calculating the SmB'B' methylation signal normalizing to the β-actin total level.

Supplementary Fig.3

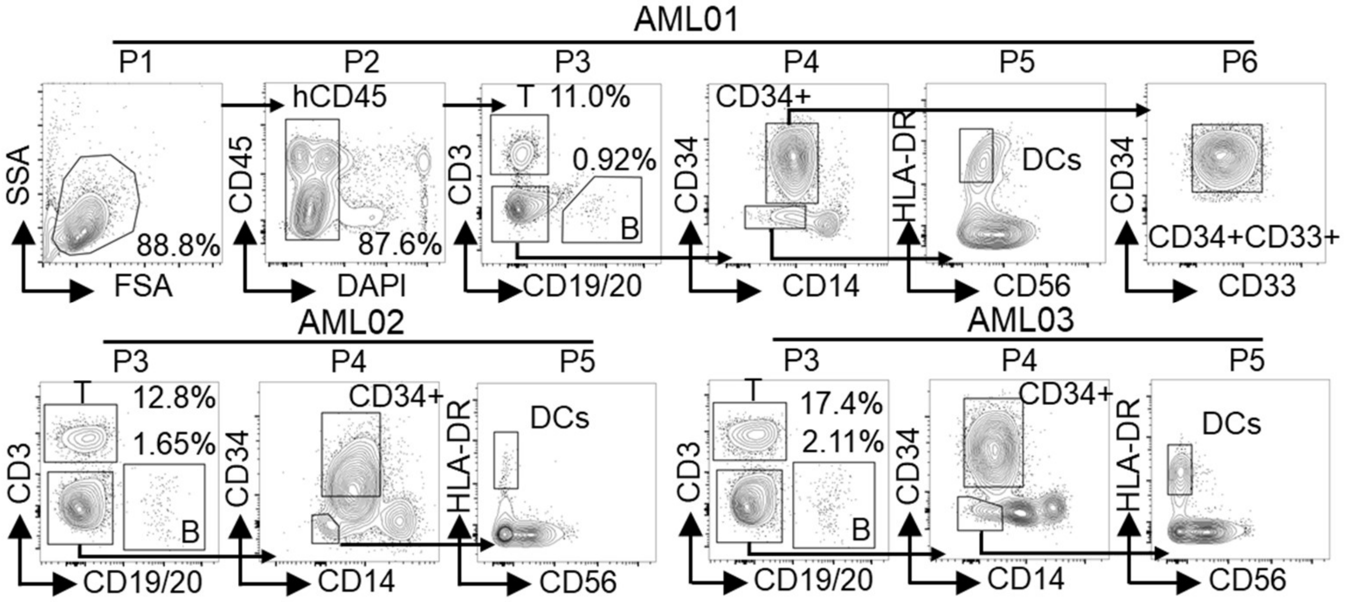

Supplementary Fig.3 Gating strategy of AML samples for ex-vivo treatment.

After thawing the samples, we enriched viable cells by removing dead cells (ensuring > 95% live cells) and then treated MNCs with vehicle (0.1%DMSO), LD2 (2.5 μM). The gating strategy for the hematopoietic subsets in the three AML samples was shown including T cells (CD3+), B cells (CD19+/CD20+), and DCs (HLA-DR+CD34-CD33-CD3-CD19-CD20-CD14-CD56-), and leukemic cells (CD34+CD33+).

Supplementary Fig.4

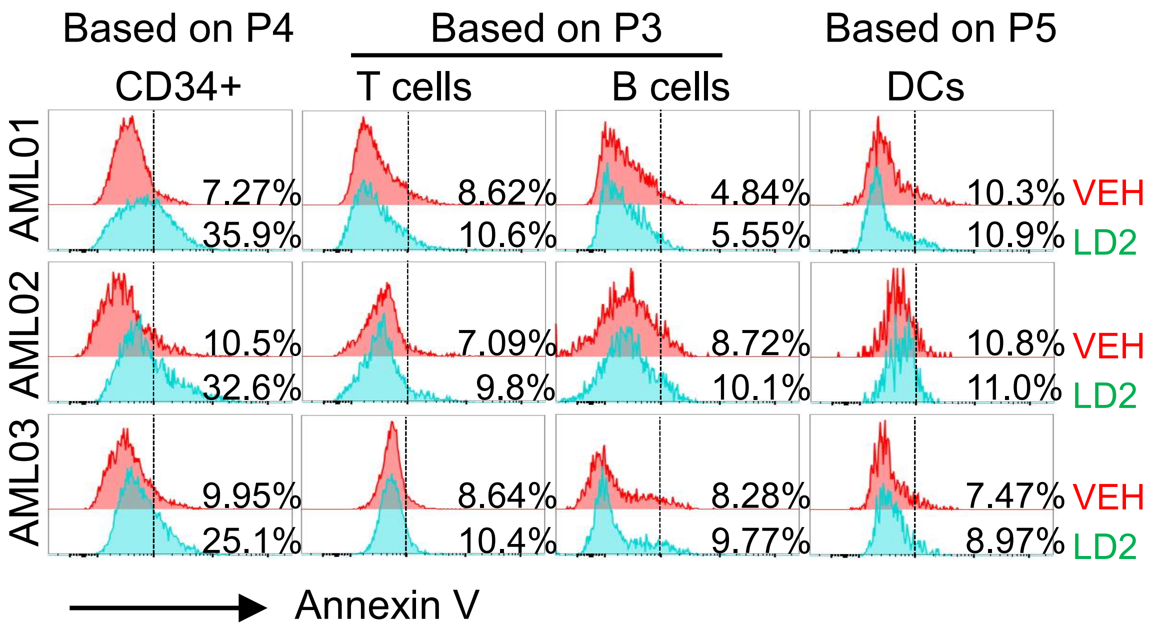

Supplementary Fig.4 Apoptosis of AML cells after ex-vivo treatment.

After 4 days treatment of vehicle (VEH) or LD2, survival of leukemic (CD34+CD33+) and immune subsets, including T cells (CD3+), B cells (CD19+/CD20+), and DCs (HLA-DR+CD34-CD33-CD3-CD19-CD20-CD14-CD56-), were assessed by Annexin-V staining. Histogram plots showed the percentage of apoptosis cells in DAPI negative cells in leukemic cells, T cells, B cells, and DCs (subsets gating shown as in Supplementary Fig.3.)

Supplementary Fig.5

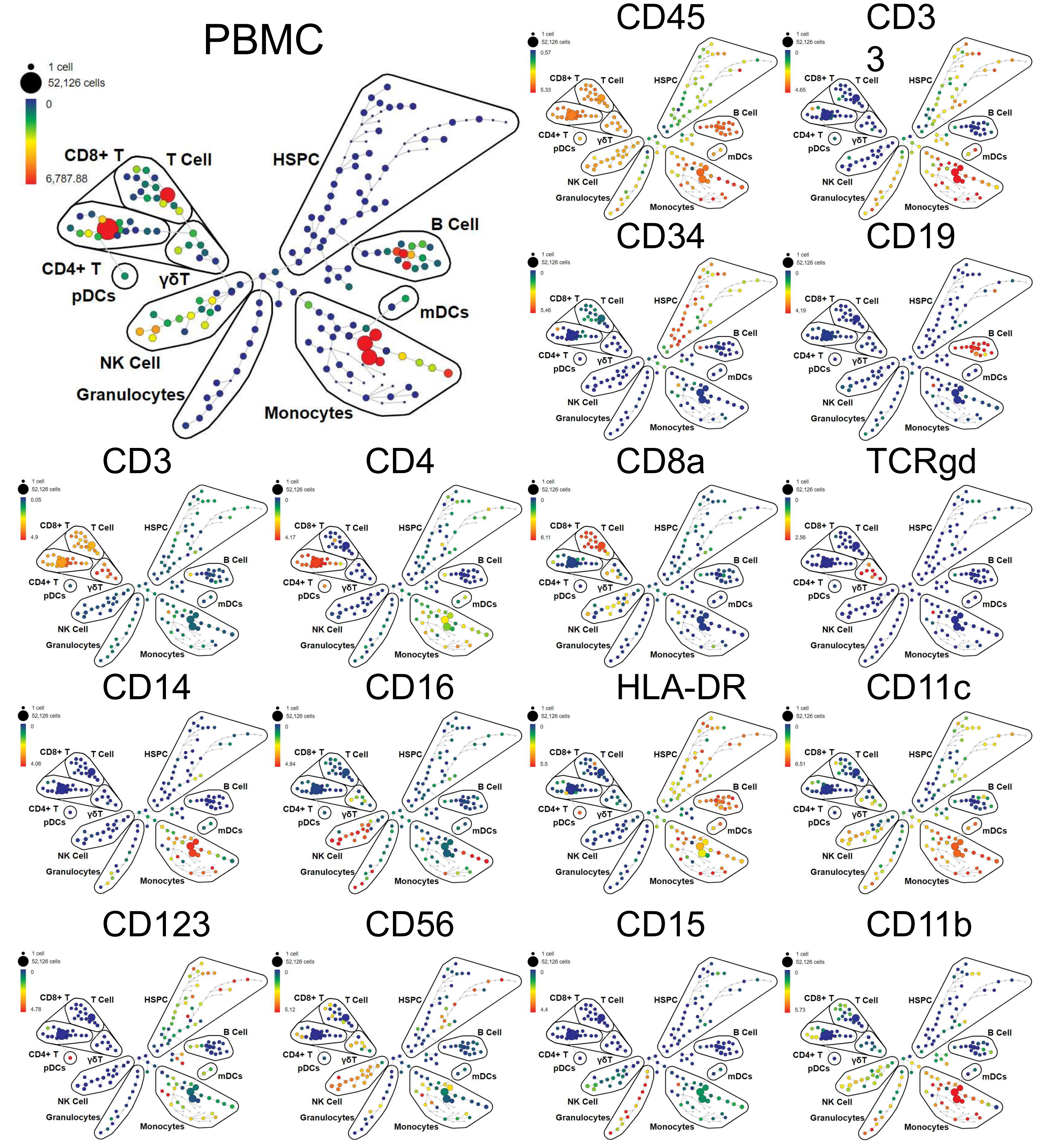

Supplementary Fig.5 CyTOF analysis of PBMCs from a healthy donor.

SPADE tree view analysis of a normal PBMC sample from one healthy donor. SPADE clustering was performed on all samples (normal and AML) simultaneously to generate the same tree view structure for all samples after vehicle or LD2 treatment. All cell events from each sample were mapped to the common tree structure. Each node of the SPADE tree is colored as a median expression of indicated phenotypic markers from low (blue) to high (red). Node size positively correlates with the number of cells mapping to the node. Immunophenotypic grouping of nodes was performed manually based on median marker expression level of each node. Shown is expression of all markers used for phenotyping (such as CD3, CD19, CD34).

Supplementary Fig.6

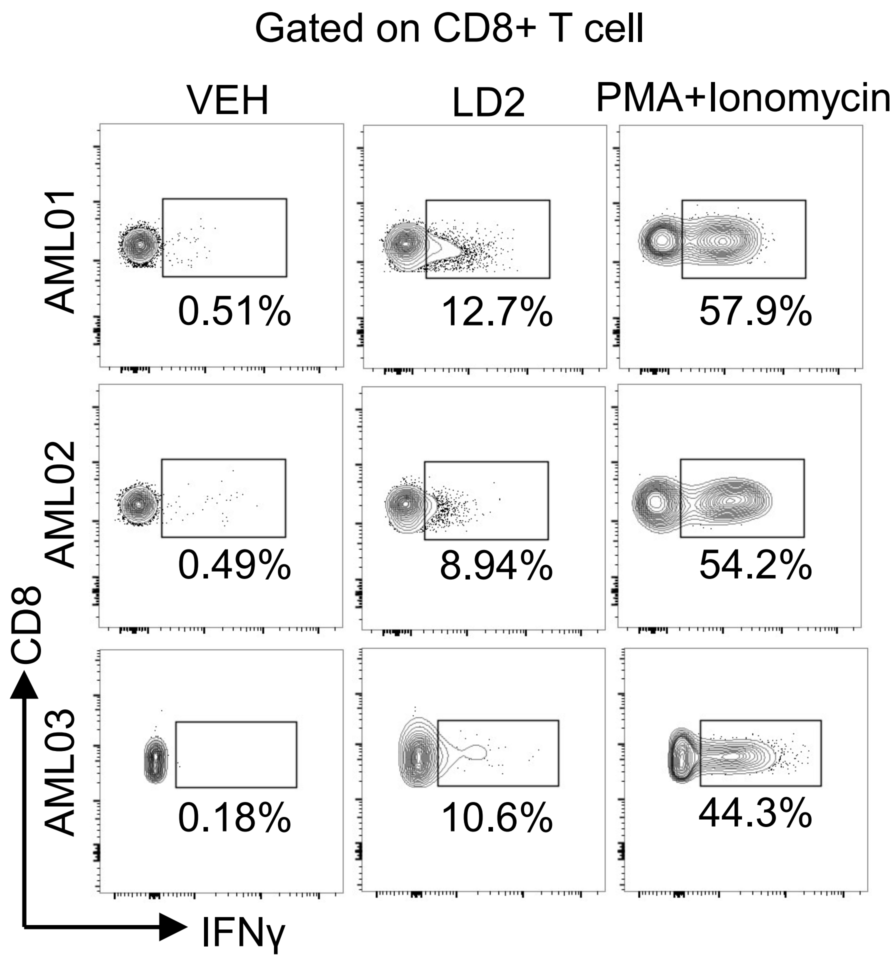

Supplementary Fig.6 Ex-vivo activation of T cells in AML samples .

Intracellular staining of IFN-γ in CD8+ T cells after LD2 treatment (or positive control) in primary AML samples described in SI. Fig.3. LD2 treatment in total of 4 days ex-vivo culture (under the BM stroma cytokine condition). For positive control, AML MNCs were first cultured without PMA/ionomycin for 90 hr, then exposed to PMA/ionomycin for last 6 hr. The plots showed the percentage of IFN-γ CD8+ T cells in each group.

Supplementary Fig.7

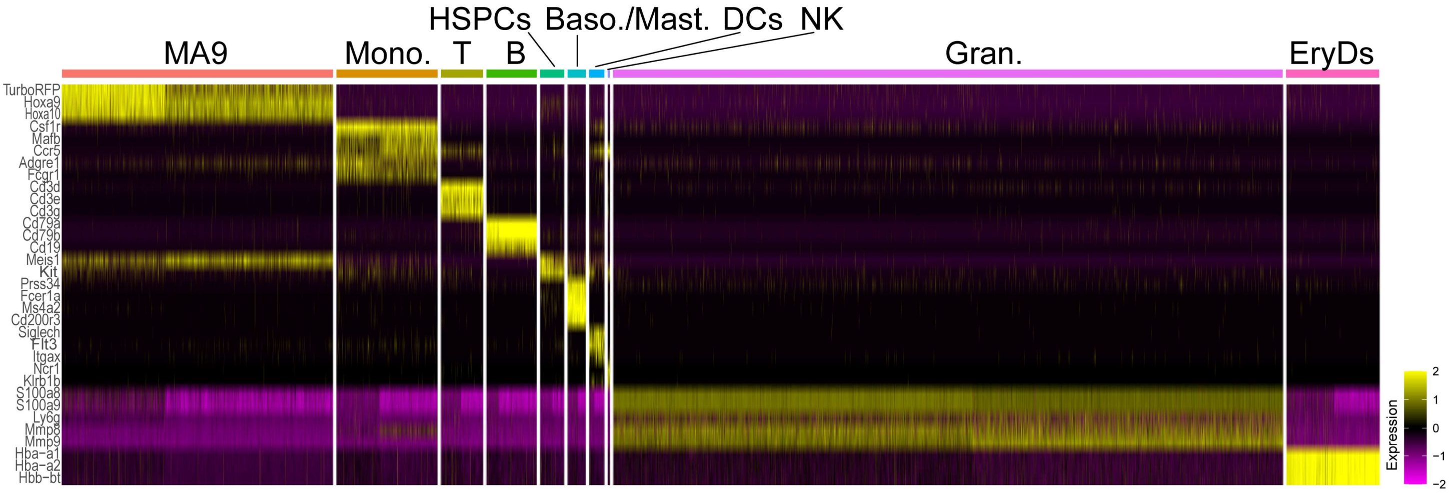

Supplementary Fig.7 Cell clustering of scRNAseq analysis.

Heatmap showing representative expression of markers of identified clusters, including MA9 cells (MA9), monocytes/macrophages (Mono.), T cells (T), B cells (B), hematopoietic stem and progenitor cells (HSPCs), basophils/mast cells (Baso./Mast.), dendritic cells (DCs), natural killer cells (NK), granulocytes (Gran.), and erythroid cells (EryDs).

Supplementary Fig.8

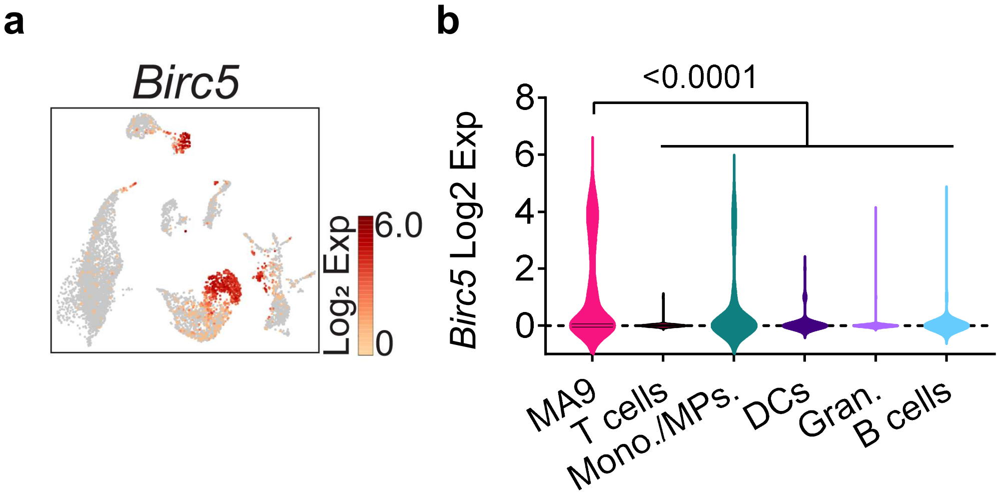

Supplementary Fig.8 Survivin is elevated in MA9 cells than other immune subsets.

(a) UMAP plot showing Birc5 (encoding Survivin) expression in different MA9 mouse BM subpopulations in Ctrl sample. (b) Histogram showed elevated expression of Birc5 in MA9 leukemia cells relative to other immune subsets. MA9 cells, n=1826; T cells, n=249; Monocytes/Macrophages, n=631; DCs, n=108; Granulocytes, n=3378; B cells, n=413. Data are presented as mean ± SEM, statistical comparisons were performed using unpaired two-sided t-test.

Supplementary Fig.9

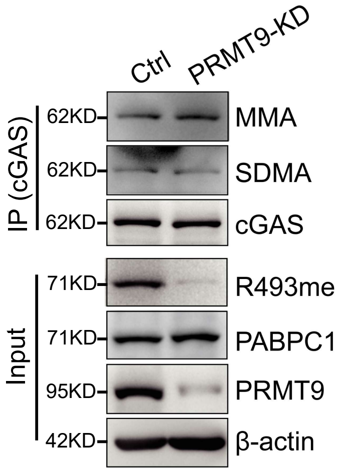

Supplementary Fig.9 PRMT9 does not catalyze cGAS methylation.

In inducible PRMT9-KD Molm13 cells, cGAS protein was IP'd after Dox induction and performed immunoblotting with mono-methylation (MMA) antibody or SDMA antibodies mixture (SYM10 [Millipore], panSDMA antibody mix [CST]). PABPC1 R493me was also blotted as a control for PRMT9 inhibition (n=1).

Supplementary Fig.1 Unprocessed western blots

Supplementary Fig. 1a

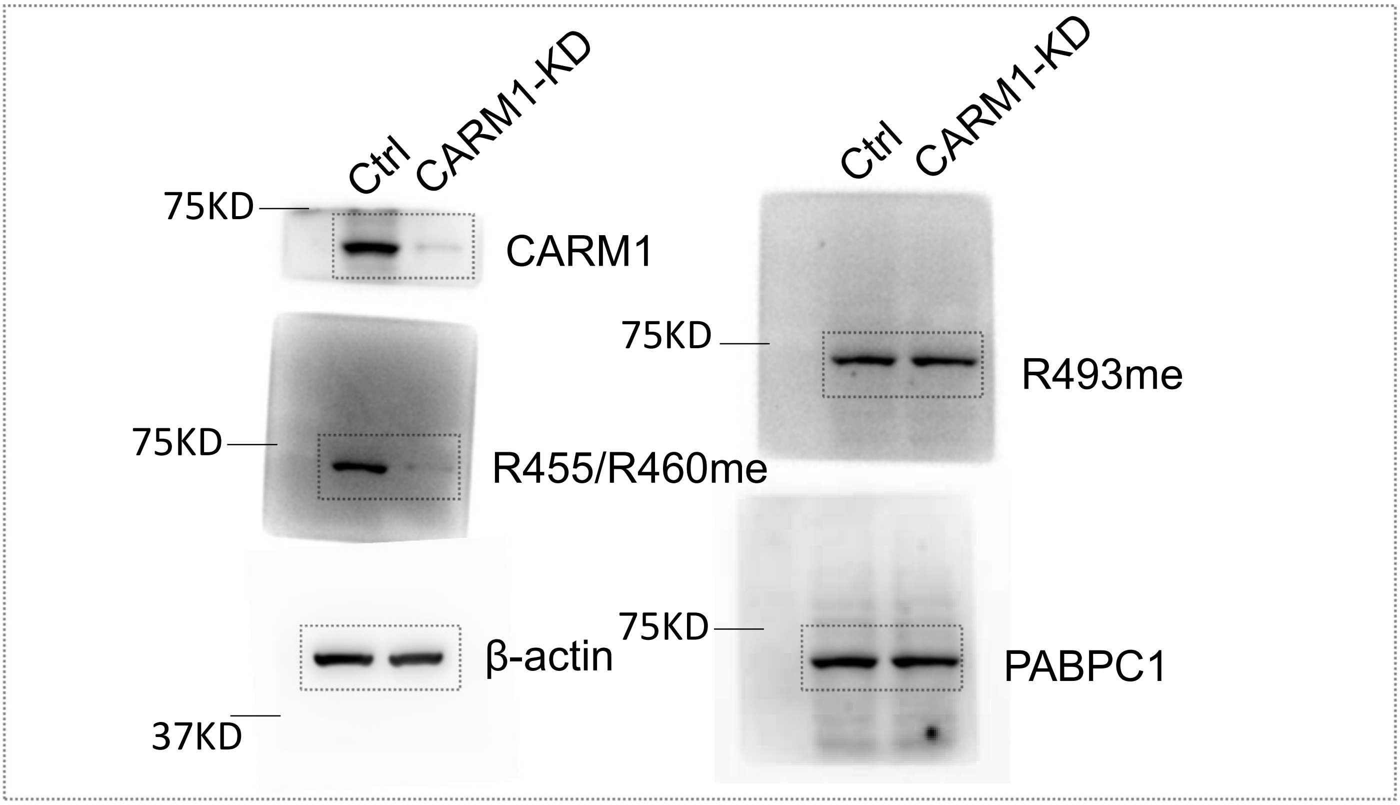

Supplementary Fig. 1b

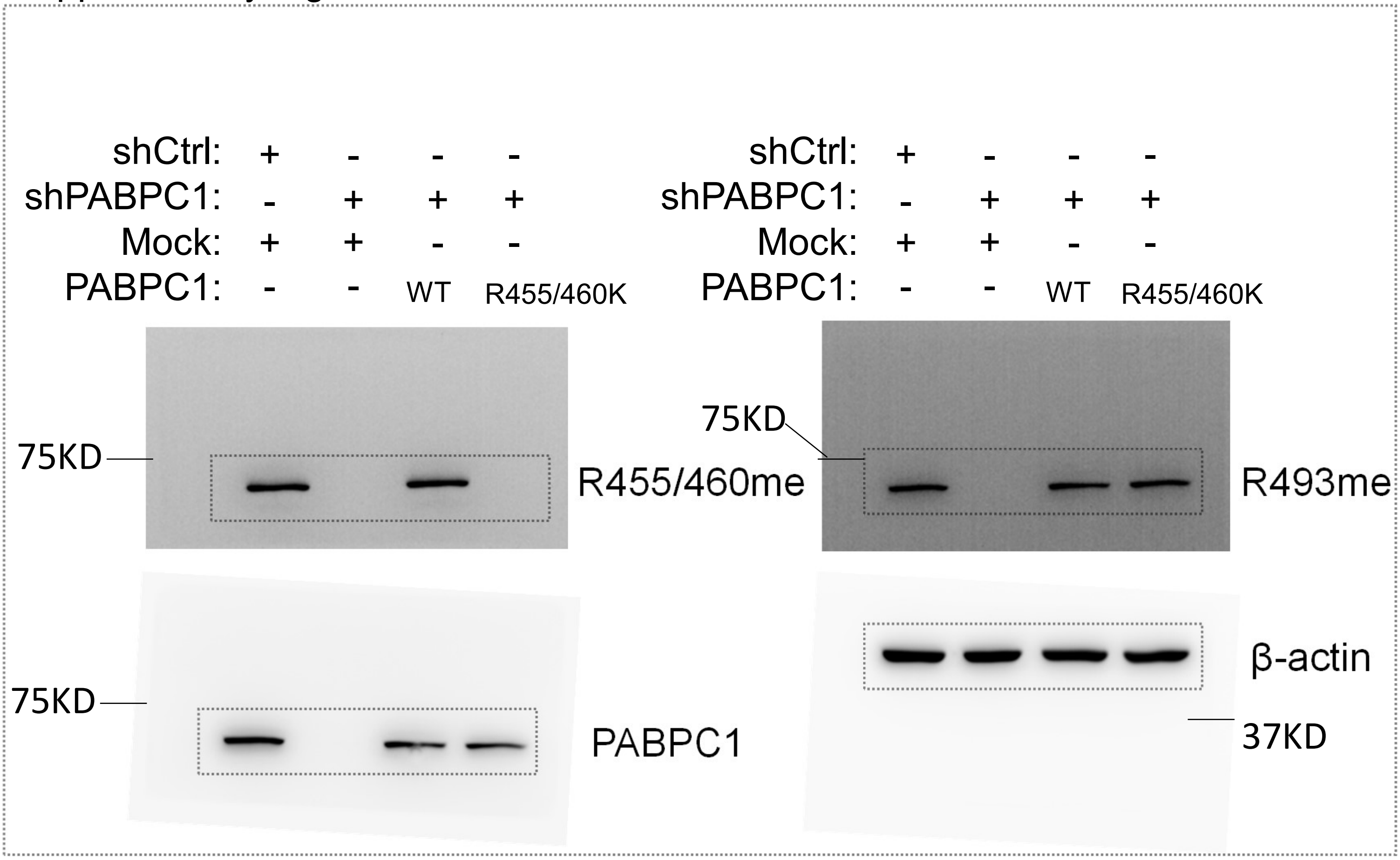

Supplementary Fig.2 Unprocessed western blots

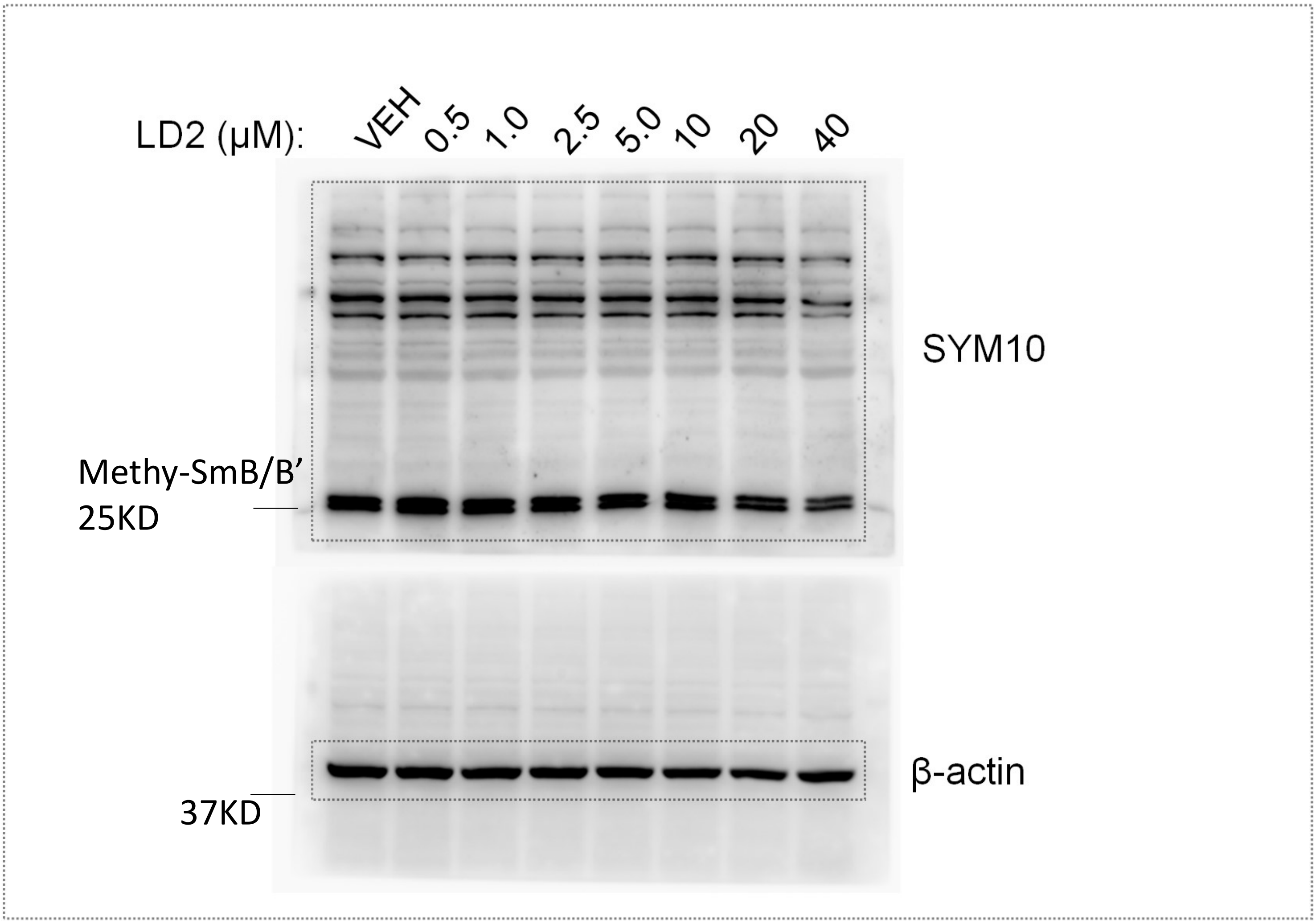

Supplementary Fig.9 Unprocessed western blots

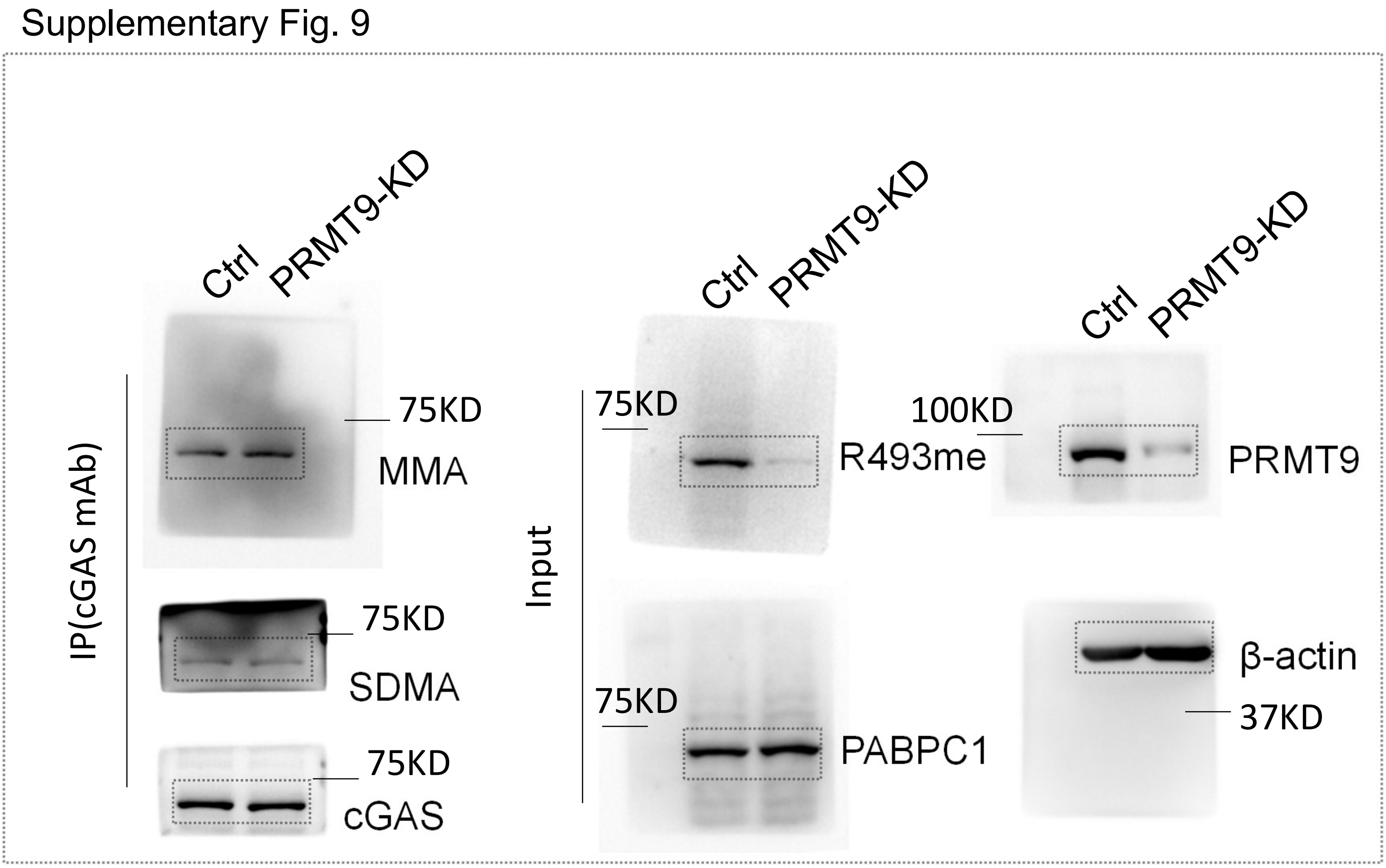

Supplement: Supplementary file 1 — Supplementary Figs. 1–9 and Tables 1–11. [file 43018_2024_736_MOESM1_ESM.pdf]
