# Supplementary material for: Targeting PRMT9-mediated arginine methylation suppresses cancer stem cell maintenance and elicits cGAS-mediated anticancer immunity
Source: Nat Cancer. 2024 Feb 27;5(4):601–24. doi: 10.1038/s43018-024-00736-x (PMC11056319; doi:10.1038/s43018-024-00736-x)
Supplement: Supplementary file 2 — Reporting Summary [file 43018_2024_736_MOESM2_ESM.pdf]

nature portfolio

Corresponding author(s): Ling Li

Last updated by author(s): Jan 17, 2024

Reporting Summary

Nature Portfolio wishes to improve the reproducibility of the work that we publish. This form provides structure for consistency and transparency in reporting. For further information on Nature Portfolio policies, see our Editorial Policies and the Editorial Policy Checklist.

Statistics

For all statistical analyses, confirm that the following items are present in the figure legend, table legend, main text, or Methods section.

n/a	Confirmed	
☐	☒	The exact sample size (*n*) for each experimental group/condition, given as a discrete number and unit of measurement
☐	☒	A statement on whether measurements were taken from distinct samples or whether the same sample was measured repeatedly
☐	☒	The statistical test(s) used AND whether they are one- or two-sided<br>*Only common tests should be described solely by name; describe more complex techniques in the Methods section.*
☒	☐	A description of all covariates tested
☐	☒	A description of any assumptions or corrections, such as tests of normality and adjustment for multiple comparisons
☐	☒	A full description of the statistical parameters including central tendency (e.g. means) or other basic estimates (e.g. regression coefficient) AND variation (e.g. standard deviation) or associated estimates of uncertainty (e.g. confidence intervals)
☐	☒	For null hypothesis testing, the test statistic (e.g. *F*, *t*, *r*) with confidence intervals, effect sizes, degrees of freedom and *P* value noted<br>*Give P values as exact values whenever suitable.*
☒	☐	For Bayesian analysis, information on the choice of priors and Markov chain Monte Carlo settings
☒	☐	For hierarchical and complex designs, identification of the appropriate level for tests and full reporting of outcomes
☐	☒	Estimates of effect sizes (e.g. Cohen's *d*, Pearson's *r*), indicating how they were calculated

Our web collection on statistics for biologists contains articles on many of the points above.

Software and code

Policy information about availability of computer code

Data collection	BD FACSDiva(V.8.0) was used to collect flow cytometric data. Aura 4.0.0 was used to collect in vivo Bioluminescence imaging data. GeneSys image acquisition software v1.5.7.0 (Syngene) was used to collect DNA gels and western blots data.
Data analysis	GraphPad Prism 9 was used to generate graphs for in vitro and in vivo assays. FlowJo (v10.8.0) was used for flow cytometric analysis. Image) software v1.53s was used to analyze the relative protein levels in western blot results. MaxQuant v1.6.17.0 was used for SILAC analysis. Cytobank software v10.3 (https://premium.cytobank.org/) was used for CyTOF analysis. Bruker stddiffgp19.3 was used for STD results analysis. Bruker Topspin 3.6 was used for CPMG results analysis. Cell Ranger v6.1.1, R version 4.0.2, package, Seurat v4.0, Harmony v1.1.0, MAST v1.26.0, SingleR v2.2.0 were used for single cell RNAseq analysis. GSEA v4.0.3, Tophat2 v2.0.8, HTseq v0.11.2, edgeR v.3.30.3, Cluster v3.0 were used for bulk RNA seq analysis. Interaction analysis of proteins carrying PRMT9-regulated R-methyl peptides was visualized by Cytoscape 3.10.1. Please see Methods section in manuscript for further details.

For manuscripts utilizing custom algorithms or software that are central to the research but not yet described in published literature, software must be made available to editors and reviewers. We strongly encourage code deposition in a community repository (e.g. GitHub). See the Nature Portfolio guidelines for submitting code & software for further information.

Data

Policy information about availability of data

All manuscripts must include a data availability statement. This statement should provide the following information, where applicable:

- Accession codes, unique identifiers, or web links for publicly available datasets
- A description of any restrictions on data availability
- For clinical datasets or third party data, please ensure that the statement adheres to our policy

All data supporting the findings of this study are available within the article and its Supplementary Information. Human AML cells Molm13 and THP1 RNA-seq raw data and Murine single-cell RNA-seq raw data have been deposited in the GEO database under accession code: GSE217195 and GSE217396. PRMT9 SILAC proteomics data have been deposited to PRIDE with he accession number: PXD039441. Previously published datasets and information info are available with the following links and accession codes: https://DepMap.org/portal/; https://www.cbioportal.org/ (TCGA PanCancer Atlas Studies); http://cancergenome.nih.gov/ (TCGA Research Network, pan-kidney transcriptomic, genomic and clinical data); BEAT AML dataset (http://vizome.org/aml/); https://servers.binf.ku.dk/bloodspot/ (BloodSpot); GEPIA (http://gepia.cancer-pku.cn/); TARGET-AML, GSE14468, GSE12417, GSE63270, GSE183415. Source data are provided with this paper. Requests for resources and reagents can be directed to the lead contact L.L.

Human research participants

Policy information about studies involving human research participants and Sex and Gender in Research.

Reporting on sex and gender	Sex and gender were not relevant in this study.
Population characteristics	Humanspecimens were obtained from the Hematopoietic tissue bank at City of Hope. The covariants associated with each sample were not obtained as they were not relevant for this study.
Recruitment	Participants were recruited by the Hematopoietic tissue bank as part of a standard procedure with informed content and no potential bias.
Ethics oversight	All subjects signed informed consent forms. Sample acquisition was approved by the COH Institutional Review Board in accordance with the Helsinki Declaration.

Note that full information on the approval of the study protocol must also be provided in the manuscript.

Field-specific reporting

Please select the one below that is the best fit for your research. If you are not sure, read the appropriate sections before making your selection.

☒ Life sciences ☐ Behavioural & social sciences ☐ Ecological, evolutionary & environmental sciences

For a reference copy of the document with all sections, see nature.com/documents/nr-reporting-summary-flat.pdf

Life sciences study design

All studies must disclose on these points even when the disclosure is negative.

Sample size	No sample size was pre-determined. Sample size and number of independent experiments are stated in the figure legend or in the Methods or Results section. Three or more independent results were used to perform statistical analyses. Studies involving independent cohorts of mice were typically performed once, with several exceptions stated in the figure legends. No specific statistical tests were applied to determine the sample size, the size was established according to our previous experience with the models used. Accordingly, we typically employed experimental cohorts of five to seven mice.
Data exclusions	No data were excluded from the analyses.
Replication	Replicates were used in all experiments as indicated in text, figure legends and methods. Animal experiments have been repeated as indicated in the figure legends. All other experiments have been repeated at least twice with consonant results. All attempts at replication were successful.
Randomization	The experiments were not randomized.
Blinding	Blinding was not done due to the requirements for case labeling and staffing needs, as knowledge of the grouping information was essential for the staff to conduct the studies.

Reporting for specific materials, systems and methods

We require information from authors about some types of materials, experimental systems and methods used in many studies. Here, indicate whether each material, system or method listed is relevant to your study. If you are not sure if a list item applies to your research, read the appropriate section before selecting a response.

Materials & experimental systems

n/a	Involved in the study
☐	☒ Antibodies
☐	☒ Eukaryotic cell lines
☒	☐ Palaeontology and archaeology
☐	☒ Animals and other organisms
☒	☐ Clinical data
☒	☐ Dual use research of concern

Methods

n/a	Involved in the study
☒	☐ ChIP-seq
☐	☒ Flow cytometry
☒	☐ MRI-based neuroimaging

Antibodies

Antibodies used

Anti-human CD3(BioLegend, Cat#317320; RRID:AB_10916519, 1:100); Anti-human CD45(BioLegend, Cat#368512; RRID:AB_2566372, 1:100); Anti-human CD33(BioLegend, Cat#366608; RRID:AB_2566107, 1:100); Anti-human CD71(BioLegend, Cat#334110; RRID:AB_2563117, 1:100); Anti-human CD11b(BioLegend, Cat#301322; RRID:AB_830644, 1:100); Anti-mouse CD11b(BioLegend, Cat#101212; RRID:AB_312795, 1:100); Anti-mouse CD3(BioLegend, Cat#100206; RRID:AB_312663, 1:100); Anti-mouse CD34(BioLegend, Cat#119310; RRID:AB_1236469, 1:100); Anti-mouse Sar1(BioLegend, Cat#108108; RRID:AB_313345, 1:100); Anti-mouse Ter119(BioLegend, Cat#116244; RRID:AB_2565872, 1:100); Annxin V(BioLegend, Cat#640920; RRID:AB_2561515, 1:100); Anti-human CD19(eBioscience, Cat#13-0199-82; RRID:AB_466388, 1:100); Anti-human CD34(eBioscience, Cat#47-0349-42; RRID:AB_2573956, 1:100); Anti-human CD38(eBioscience, Cat#11-0388-42; RRID:AB_10547895, 1:100); Anti-human CD14(eBioscience, Cat#45-0149-42; RRID:AB_1518736, 1:100); Anti-human CD15(eBioscience, Cat#48-0159-42; RRID:AB_2016661, 1:100); Anti-human CD45(eBioscience, Cat#48-9459-42; RRID:AB_1603240, 1:100); Anti-mouse CD3(eBioscience, Cat#13-0032-82; RRID:AB_2572762, 1:100); Anti-mouse CD4(eBioscience, Cat#13-0041-85; RRID:AB_466326, 1:100); Anti-mouse CD8a(eBioscience, Cat#13-0081-85; RRID:AB_466347, 1:100); Anti-mouse B220(eBioscience, Cat#13-0452-85; RRID:AB_466450, 1:100); Anti-mouse IGM(eBioscience, Cat#13-5790-85; RRID:AB_466676, 1:100); Anti-mouse CD19(eBioscience, Cat#13-0193-85; RRID:AB_657658, 1:100); Anti-mouse CD11b(eBioscience, Cat#13-0112-85; RRID:AB_466360, 1:100); Anti-mouse CD11c(eBioscience, Cat#13-0114-82; RRID:AB_466363, 1:100); Anti-mouse NK1.1(eBioscience, Cat#13-5941-85; RRID:AB_466805, 1:100); Anti-mouse GR1(eBioscience, Cat#13-5931-85; RRID:AB_466801, 1:100); Anti-mouse Ter119(eBioscience, Cat#13-5921-85; RRID:AB_466798, 1:100); Anti-mouse CD127(eBioscience, Cat#13-1271-82; RRID:AB_466588, 1:100); Anti-mouse GR1(eBioscience, Cat#12-5931-82; RRID:AB_466045, 1:100); Anti-mouse CD117(eBioscience, Cat#47-1172-82; RRID:AB_1582226, 1:100); Anti-mouse CD45.2(eBioscience, Cat#11-0454-85; RRID:AB_465062, 1:100); APC anti-mouse CD34 Antibody(Biolegend, Cat#119310, 1:100); Brilliant Violet 605 anti-mouse Ly-6A/E (Sca-1) Antibody(Biolegend, Cat#108134, 1:100); APC/Cyanine7 anti-mouse CD117 (c-kit) Antibody(Biolegend, Cat#135136, 1:100); PE/Cy7 anti-mouse CD16/32 Antibody(Biolegend, Cat#101318, 1:100); Brilliant Violet 605 Streptavidin(Biolegend, Cat#405229, 1:100); APC Annexin V(Biolegend, Cat#640941, 1:100); percp/cyanin5.5 anti-human CD14(Biolegend, Cat#325622, 1:100); APC anti-human CD45(Biolegend, Cat#368512/2D1, 1:100); PE/CY7 anti-human CD11B(Biolegend, Cat#301322/ICRF44, 1:100); FITC anti-human CD15 (SSEA-1) Antibody(Biolegend, Cat#301904/HI98, 1:100); APC anti-mouse CD115(Biolegend, Cat#135510, 1:100); APC/CY7 anti-mouse CD3(Biolegend, Cat#100222, 1:100); CD11B-APC anti-mous and human clone:M1/70(Biolegend, Cat#101212, 1:100); Pacific Blue Anti-mouse Ly-6G/Ly-6C (Gr-1) Clone RB6-8C5(Biolegend, Cat#108430, 1:100); APC/Cyanine7 anti-human CD34(Biolegend, Cat#343614, 1:100); FITC Annexin V(Biolegend, Cat#640906, 1:100); FITC anti-human CD38 Antibody(Biolegend, Cat#356610, 1:100); FITCBV421 anti-human CD15 (SSEA-1) Antibody(Biolegend, Cat#323040/W6D3, 1:100); PE anti-mouse IFN-γ Antibody(Biolegend, Cat#505807, 1:100); FITC anti-human/mouse Granzyme B Recombinant Antibody(Biolegend, Cat#372205, 1:100); Pacific Blue™ anti-mouse CD8a Antibody(Biolegend, Cat#100728, 1:100); APC anti-mouse NK-1.1 Antibody(Biolegend, Cat#108709, 1:100); PE/Cyanine7 anti-mouse CD45.1 Antibody(Biolegend, Cat#110730, 1:100); Alexa Fluor® 700 anti-mouse CD4 Antibody(Biolegend, Cat#100429, 1:100); Alexa Fluor® 700 anti-human CD4 Antibody(Biolegend, Cat#300526, 1:100); Brilliant Violet 605™ anti-human CD8a Antibody(Biolegend, Cat#301039, 1:100); Pacific Blue™ anti-human CD14 Antibody(Biolegend, Cat#301816, 1:100); PE anti-human CD197 (CCR7) Antibody(Biolegend, Cat#353203, 1:100); FITC anti-human CD25 Antibody(Biolegend, Cat#356105, 1:100); PE/Cyanine7 anti-human HLA-DR Antibody(Biolegend, Cat#307615, 1:100); Pacific Blue™ anti-mouse CD45.2 Antibody(Biolegend, Cat#109819, 1:100); PE anti-human CD16(Biolegend, Cat#980102, 1:100); BV510- mouse anti-human CD38 Antibody(Biolegend, Cat#356611, 1:100); AF700 anti-mouse CD4 antibody(Biolegend, Cat#100430, 1:100); PE anti-human IFN-gamma Antibody(Biolegend, Cat#506506, 1:100); Purified anti-human FOXP3 Antibody(Biolegend, Cat#320102, 1:100); Pacific Blue anti-mouse/human CD11b Antibody(Biolegend, Cat#101224, 1:100); FITC anti-human CD279 (PD-1)(Biolegend, Cat#329903, 1:100); PE/Dazzle 594 anti-human CD152 (CTLA-4)(Biolegend, Cat#369615, 1:100); Pacific Blue anti-human CD366 (Tim-3)(Biolegend, Cat#345041, 1:100); PE/Cyanine7 anti-human TIGIT (VSTM3)(Biolegend, Cat#372713, 1:100); FITC anti-mouse CD279 (PD-1) Antibody(Biolegend, Cat#135213, 1:100); PE/Cyanine7 anti-mouse CD223 (LAG-3) Antibody(Biolegend, Cat#125226, 1:100); Brilliant Violet 605™ anti-mouse CD152 Antibody(Biolegend, Cat#106323, 1:100); Alexa Fluor® 647 anti-mouse CD366 (Tim-3) Antibody(Biolegend, Cat#119744, 1:100): PE/Dazzle™ 594 anti-mouse TIGIT (Vstm3) Antibody(Biolegend, Cat#142110, 1:100): APC anti-human CD15 (SSEA-1) Antibody(Biolegend, Cat#301908, 1:100); PE/Dazzle™ 594 anti-mouse CD69 Antibody(Biolegend, Cat#104536, 1:100); APC/Cyanine7 anti-human CD3 Antibody (SK7)(Biolegend, Cat#344818, 1:100); PE anti-human CD56 (NCAM) Antibody(Biolegend, Cat#362508, 1:100); APC anti-mouse NK-1.1 Antibody (S17016D) (Biolegend, Cat#156506, 1:100); Pacific Blue™ anti-human CD16 Antibody(Biolegend, Cat#302024, 1:100); Alexa Fluor® 700 anti-human CD20 Antibody(Biolegend, Cat#302322, 1:100); PE/Dazzle™ 594 anti-human CD34 Antibody(Biolegend, Cat#343533, 1:100); Brilliant Violet 605™ anti-mouse CD25 Antibody(Biolegend, Cat#102036, 1:100); PE anti-mouse/human CD44 Antibody(Biolegend, Cat#103024, 1:100); PE/Cyanine7 anti-mouse CD62L Antibody(Biolegend, Cat#104418, 1:100); Pacific Blue™ anti-mouse FOXP3

Antibody(Biolegend, Cat#126410, 1:100); PE/Cyanine7 anti-human/mouse Granzyme B Recombinant Antibody(Biolegend, Cat#372214, 1:100); Brilliant Violet 605™ anti-mouse CD8a Antibody(Biolegend, Cat#100744, 1:100); Mouse mAb anti-β-Actin(Cell Singaling Technology, Cat#3700S; RRID:AB_2242334, 1:2000); Rabbit mAb anti-HSP90(Cell Singaling Technology, Cat#4877S; RRID:AB_2233307, 1:2000); Rabbit mAb anti-H3K27ac(Abcam, Cat#ab4729; RRID:AB_2118291, 1:1000); Rabbit mAb anti-Histon H3(Cell Singaling Technology, Cat#4499S; RRID:AB_10544537, 1:1000); Rabbit pAb anti-Histon H4(Abcam, Cat#ab10158; RRID:AB_296888, 1:1000); Rabbit pAb anti-HA tag(Abcam, Cat#ab9110; RRID:AB_307019, 1:2000); Mouse mAb anti-FLAG(Sigma-aldrich, Cat#F1804; RRID:AB_262044, 1:5000); Mouse mAb anti-Myc(Santa Cruz, Cat#sc-40; RRID:AB_627268, 1:1000); Rabbit pAb anti-PABPC1-R493me(This paper, 1:1000); Rabbit pAb anti-PABPC1-R493 Ctrl(This paper, 1:1000); Mouse mAb anti-CREB1(Santa Cruz Cat#sc-240; RRID:AB_627302, 1:1000); Rabbit pAb anti-mouse Prmt9(This paper, 1:1000); Mouse mAb anti-human PRMT9(Millipore, Cat#MABE1112; RRID:AB_2801509, 1:1000); Rabbit anti-SAMHD1(Proteintech, Cat#12586-1-AP; RRID:AB_2183496, 1:1000); Rabbit anti-RUNX1(Proteintech, Cat#25315-1-AP; RRID:AB_2880026, 1:1000); Rabbit mAb anti-CEBPA(Cell Singaling Technology, Cat#8178T, 1:1000); Rabbit anti-pan-MMA(Cell Singaling Technology, Cat#8015S; RRID:AB_10891776, 1:1000); Rabbit anti-pan-SDMA(Cell Singaling Technology, Cat#13222S; RRID:AB_2714013, 1:1000); Rabbit anti-pan-ADMA(Cell Singaling Technology, Cat#13522S; RRID:AB_2665370, 1:1000); Rabbit anti-PABPC1(Cell Singaling Technology, Cat#4992; RRID:AB_10693595, 1:1000); Mouse mAb anti-PABPC1(Santa Cruz, Cat#sc-32318; RRID:AB_628097, 1:1000); Rabbit anti-PRMT1(Cell Singaling Technology, Cat#2449S; RRID:AB_2237696, 1:1000); Rabbit mAb anti-PRMT5(Abcam, Cat#ab109451; RRID:AB_10863428, 1:2000); Rabbit mAb anti-MEP50(Abcam, Cat#ab154190, 1:1000); Rabbit mAb anti-CARM1(Cell Singaling Technology, Cat#3379S; RRID:AB_2068433, 1:1000); Rabbit anti-GST(Proteintech, Cat#10000-0-AP;RRID:AB_11042316, 1:3000); Rabbit mAb anti-PABPC1 R455/R460me(Cell Singaling Technology, Cat#3505S; RRID:AB_2298971, 1:1000); Rabbit anti-FLT3 (S-18)(Santa Cruz, Cat#sc-480; RRID:AB_2104968, 1:1000); FLT3 R972/R973me(Xin He et al., 2018, 1:1000); Rabbit pAb anti-H3R8me(Abcam, Cat#ab130740; RRID:AB_2801510, 1:1000); Rabbit mAb anti-p54(nrb)(Cell Singaling Technology, Cat#10162S, 1:1000); Rabbit mAb anti-γH2AX(Cell Singaling Technology, Cat#9718S; RRID:AB_2118009, 1:1000); Rabbit mAb anti-cGAS(Cell Singaling Technology, Cat#79978S; RRID:AB_2905508, 1:1000); Rabbit mAb anti-XRN2(Cell Singaling Technology, Cat#13760S; RRID:AB_2798309, 1:1000); Rabbit mAb anti-pCHK1 (S345)(Cell Singaling Technology, Cat #2348, 1:1000); Rabbit mAb anti-CHK1(Cell Singaling Technology, Cat #37010, 1:1000); Rabbit mAb anti-pCHK2 (T68)(Cell Singaling Technology, Cat#2197, 1:1000); Rabbit mAb anti-CHK2(Cell Singaling Technology, Cat#6334, 1:1000); Rabbit pAb anti-DDX3X(Millipore Cat#09-860; RRID:AB_1977147, 1:1000); InVivoMAb anti-mouse PD-1 (CD279)(BioXCell, Cat#BE0146; RRID:AB_10949053).

Validation

The antibodies used are established in the field and have been used by a number of groups. RRID was provided in antibody information listed above.

Eukaryotic cell lines

Policy information about cell lines and Sex and Gender in Research

Cell line source(s)

Molm13 (ACC 554, DSMZ), MV4-11 (CRL-9591, ATCC), THP1 (TIB-202, ATCC), NB4 (ACC 207, DSMZ), U937 (CRL-1593.2, ATCC), HL-60 (CCL-240, ATCC), and MA9.6ITD (Gift from Dr. James Mulloy), RAJI (ACC 319, DSMZ), UPN1 (CVCL_A795, Cellosaurus), BL41 (ACC 160, DSMZ), Rec1 (ACC 584, DSMZ), OCI-Ly3 (ACC 761, DSMZ), A20 (Gift from Dr. Yangxin Fu), HEK293FT (R70007, Thermo Fisher), DMS273 (Gift from Dr. Ravi Salgia), DMS114 (Gift from Dr. Ravi Salgia), SW1573 (Gift from Dr. Edward Wang), A549 (Gift from Dr. Edward Wang), SW620 (CCL-227, ATCC), HCT116 (CCL-247, ATCC), HepG2 (HB-8065, ATCC), PC3 (Gift from Dr. Saul Priceman), DU145 (Gift from Dr. Saul Priceman), MDA-MB-231 (CRM-HTB-26, ATCC), HT1197 (CRL-1473, ATCC), A172 (CRL-1620, ATCC), MIAPACA2 (CRM-CRL-1420, ATCC), and HT1080 (CCL-121, ATCC)

Authentication

MA9.6ITD were established by Dr. James Mulloy. DMS273 and DMS114 are gift from Dr. Ravi Salgia, SW1573 and A549 are gift from Dr. Edward Wang, PC3 and DU145 are gift from Dr. Saul Priceman. A20 is gift from Dr. Yangxin Fu. These cells were not authenticated further. All other cell lines are from ATCC, DSMZ, Thermo Fisher, or Cellosaurus.

Mycoplasma contamination

All cell lines were tested negative for mycoplasma.

Commonly misidentified lines
(See ICLAC register)

A549 was gift from Dr. Edward Wang, PC3 and DU145 were gift from Dr. Saul Priceman.

Animals and other research organisms

Policy information about studies involving animals; ARRIVE guidelines recommended for reporting animal research, and Sex and Gender in Research

Laboratory animals

In all the experiments, male and female, 6-10 wks old, WT C57BL/6J (JAX #000664), B6(Cg)-Rag2tm1.1Cgn/J (JAX #008449, Rag2-/-), B6(Cg)-Ifnar1tm1.2Ees/J (JAX #028288, Ifnar1-/-), Kmt2atm2(MLLT3)Thr/KsyJ (JAX #009079, MLL-AF9 knockin), B6.129S(C)-Batf3tm1Kmm/J (JAX #013755, Batf3-/-), NOD.Cg-Prkdcscid Il2rgtm1Wjl/SzJ (JAX #005557, NSG), NOD.Cg-Prkdcscid Il2rgtm1Wjl Tg(CMV-IL3,CSF2,KITLG)1Eav/MloySzJ (JAX #013062, NSGS), and NOD.Cg-Prkdcscid H2-K1tm1Bpe H2-Ab1em1Mvw H2-D1tm1Bpe Il2rgtm1Wjl/SzJ JAX #025216, NSG-MHC I/II DKO) mice, B6-Ly5.1 (CD45.1, NCI 564) and BALB/c (NCI 028) mice were available from outside vendor. Male and female mice were housed at the COH Animal Resource Center. All care and experimental procedures followed established institutional guidelines. Mouse room is conditioned at 14-hour light/10-hour dark cycle, temperatures of 65-75° F with 40-60 humidity. The procedure was in accordance with a protocol approved by the Institutional Animal Care and Use Committee at COHCCC.

Wild animals

No wild animals used in this study.

Reporting on sex

Sex and genders were not considered in this study design.

Field-collected samples

No field-collected samples in this study.

Ethics oversight	Mice purchased were acclimated to housing conditions for at least one week at the COH Animal Resource Center prior to experiments. Colonies for each mouse strain were maintained in the same animal facility. All mouse experiments procedures were completed in accordance with the Guidelines for the Care and Use of Laboratory Animals and were approved by the Institutional Animal Care and Use Committees at COHCCC. The experiments were performed in accordance with a protocol approved by the COHCCC Institutional Animal Care and Use Committees (15046). The maximal tumor size (humane endpoint) permitted by IACUC is 15mm (diameter). All the animals were euthanized before the tumor size reached 15mm in diameter. All our animals' maximal tumor size did not exceed 15mm.

Note that full information on the approval of the study protocol must also be provided in the manuscript.

Flow Cytometry

Plots

Confirm that:

☒ The axis labels state the marker and fluorochrome used (e.g. CD4-FITC).

☒ The axis scales are clearly visible. Include numbers along axes only for bottom left plot of group (a 'group' is an analysis of identical markers).

☒ All plots are contour plots with outliers or pseudocolor plots.

☒ A numerical value for number of cells or percentage (with statistics) is provided.

Methodology

Sample preparation	BM was obtained from mouse tibias and femurs of both legs by aspiration. Spleens were removed from mice and pressed with the end of a syringe. Cells were washed with PBS containing 1% FBS and then passed through a 70-µm cell strainer and subjected to lysis of red blood cells. Before flow cytometry analysis, cells were washed twice in PBS containing 1% FBS and stained with indicated antibodies in the same buffer for 25 minutes at 4°C.
Instrument	BD LSRFortessa X-20; BD LSR II; BD Fusion
Software	BD FACSDiva Software (version 8.0); FlowJo (v10.8.0)
Cell population abundance	The abundance of the relevant cell populations within post-sort fractions were determined by analyzing again using BD Fusion, Fortessa X-20 or LSR II.
Gating strategy	All samples are FSC-A and SSC-A gated, followed by FSC-A/FSC-H, SSC-A/SSC-H gating to select singlets. Subsequent relevant gating was set based on appropriate compensation using single-stained compensation controls.

☒ Tick this box to confirm that a figure exemplifying the gating strategy is provided in the Supplementary Information.
