# Supplementary figures and images for: Targeting PRMT9-mediated arginine methylation suppresses cancer stem cell maintenance and elicits cGAS-mediated anticancer immunity
Source: Nat Cancer. 2024 Feb 27;5(4):601–24. doi: 10.1038/s43018-024-00736-x (PMC11056319; doi:10.1038/s43018-024-00736-x)

Fig. 1 Unprocessed western blots

Fig.1h

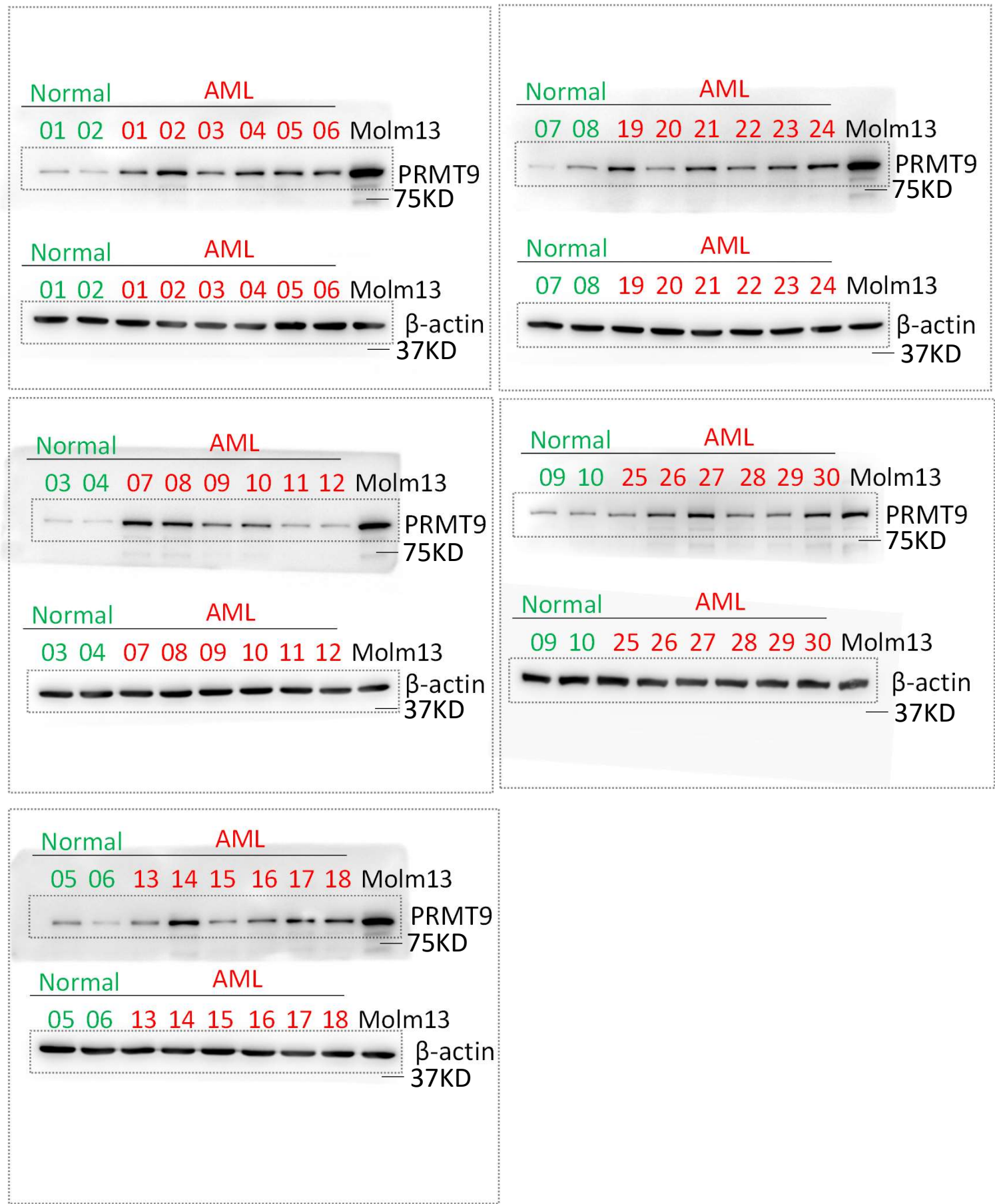

Supplement: Supplementary file 5 — Unprocessed immunoblots. [file 43018_2024_736_MOESM5_ESM.pdf]

Fig. 2 Unprocessed western blots

Fig.2a

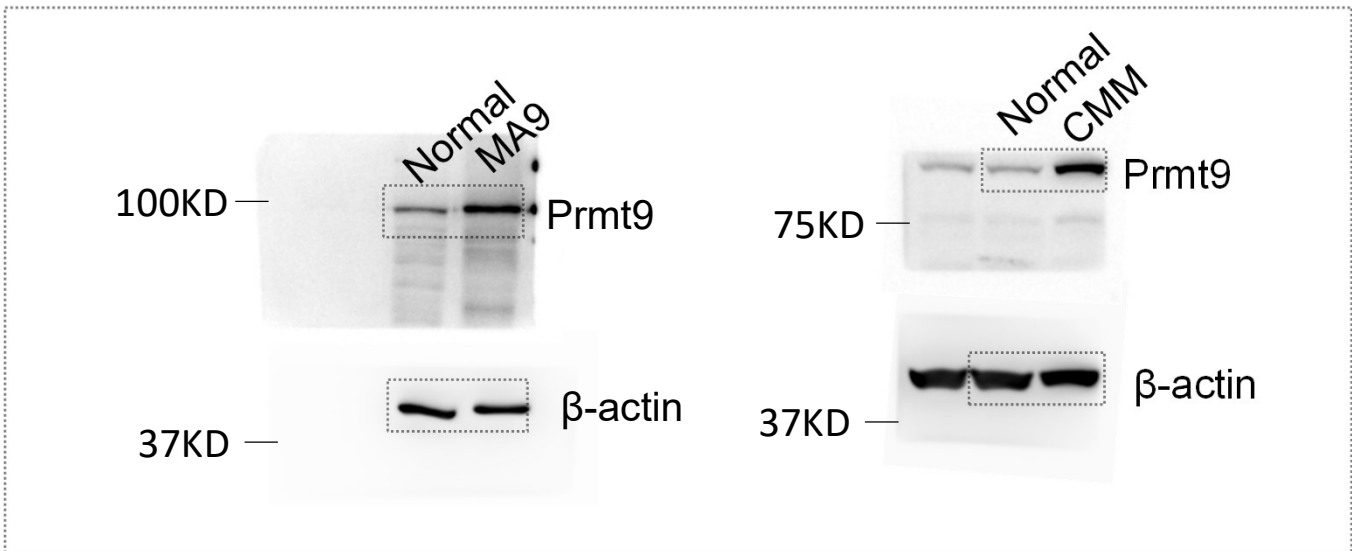

Fig.2c

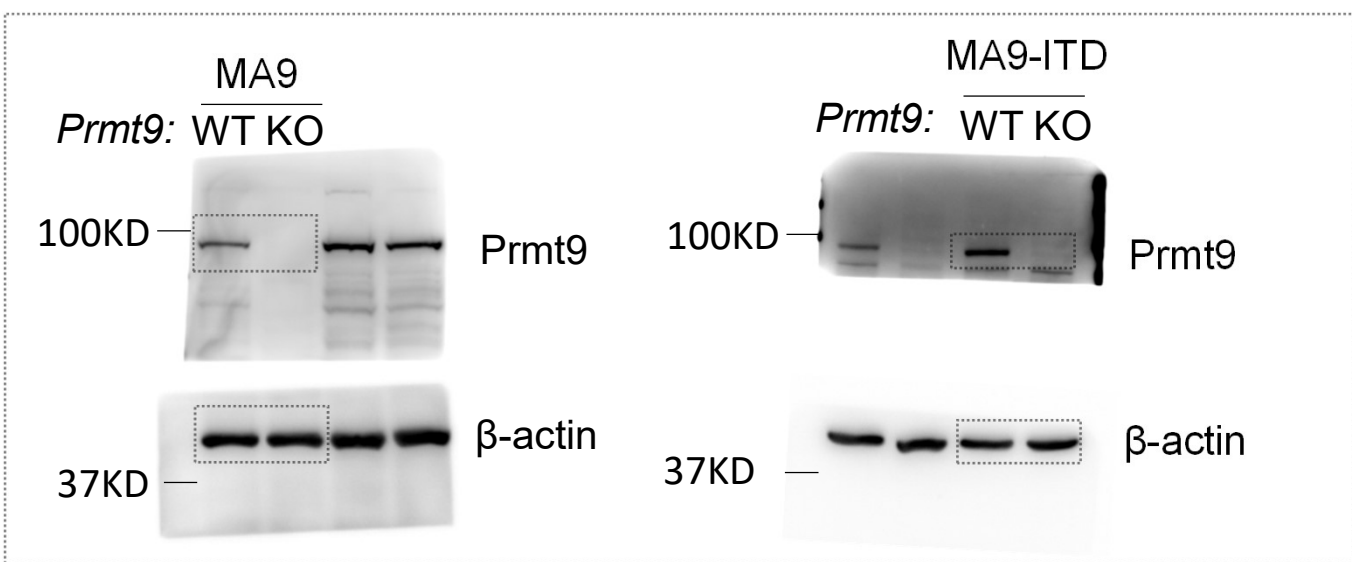

Fig.2i

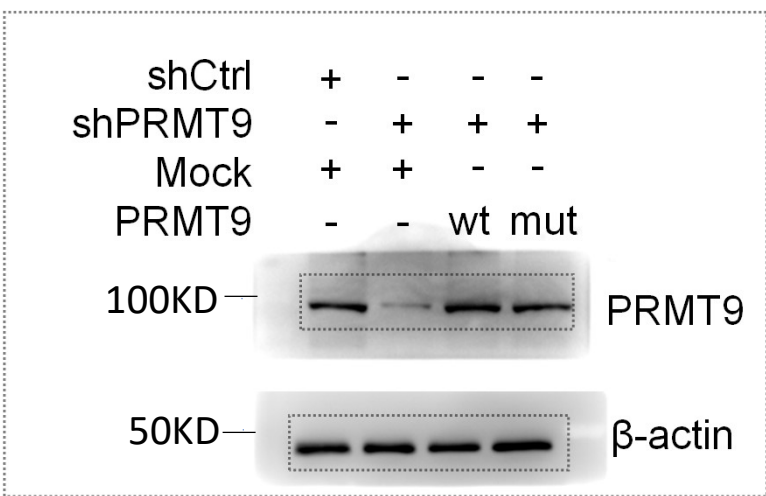

Fig.2p

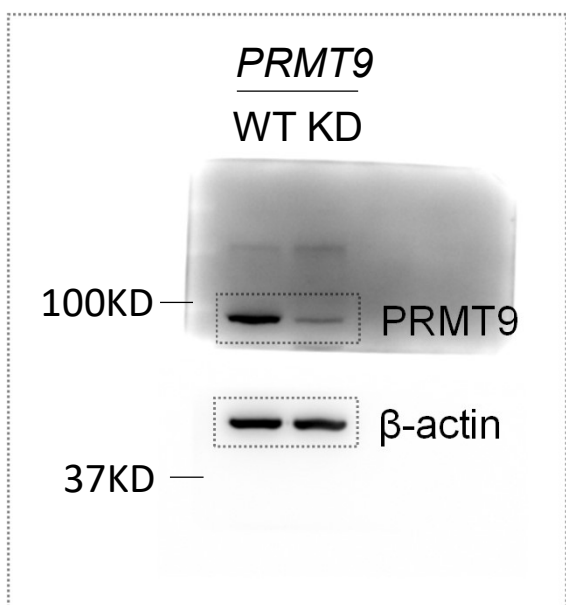

Supplement: Supplementary file 7 — Unprocessed immunoblots. [file 43018_2024_736_MOESM7_ESM.pdf]

Fig. 4 Unprocessed western blots

Fig. 4d

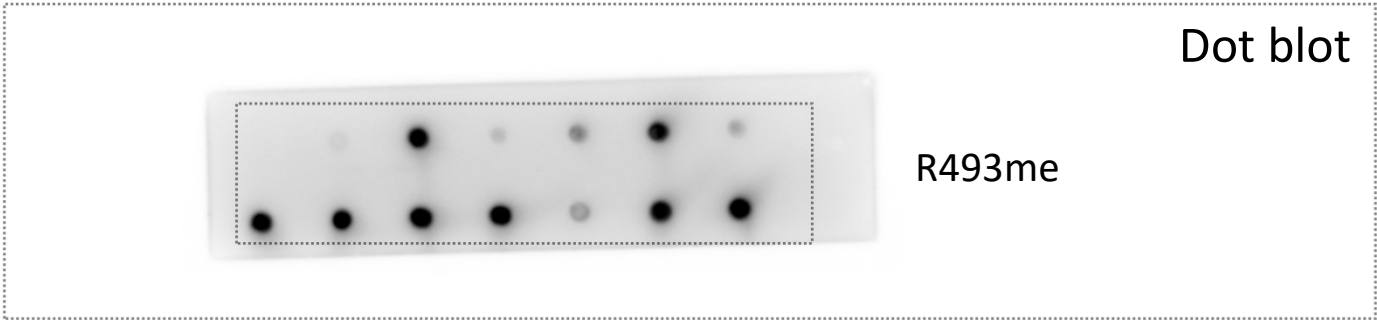

Fig. 4e

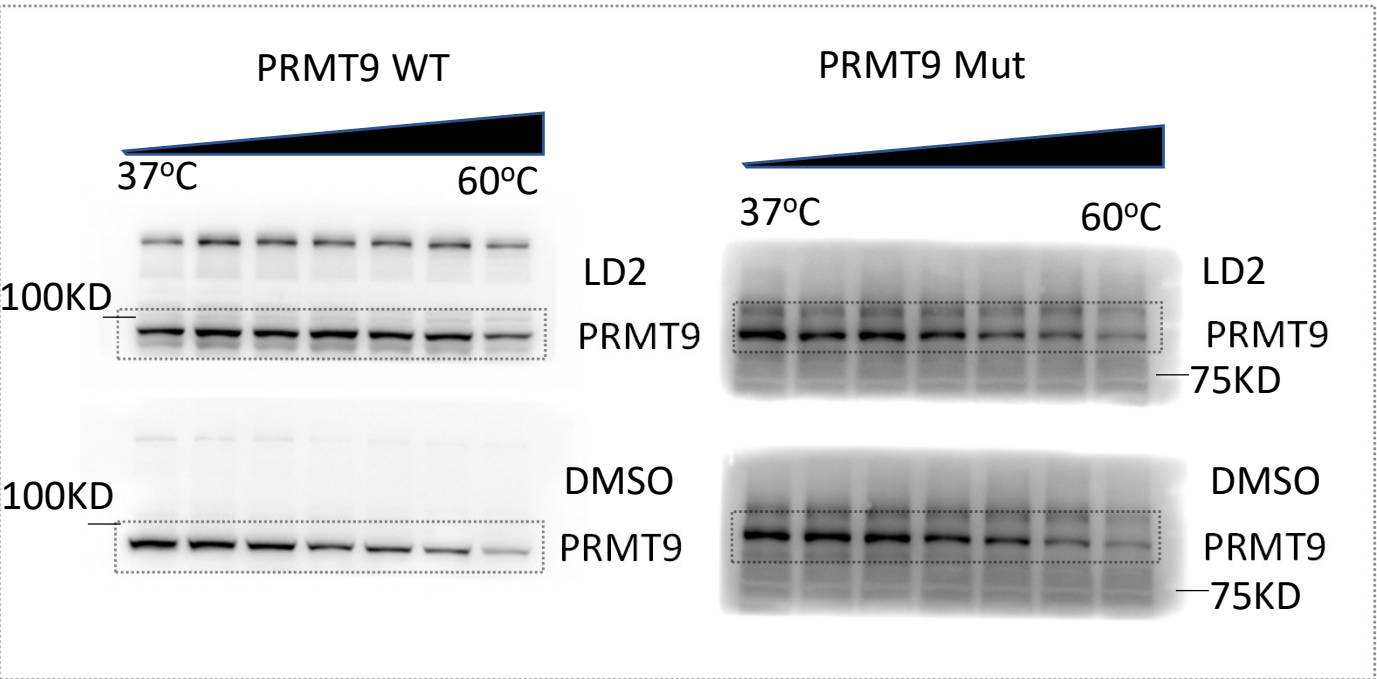

Fig. 4l

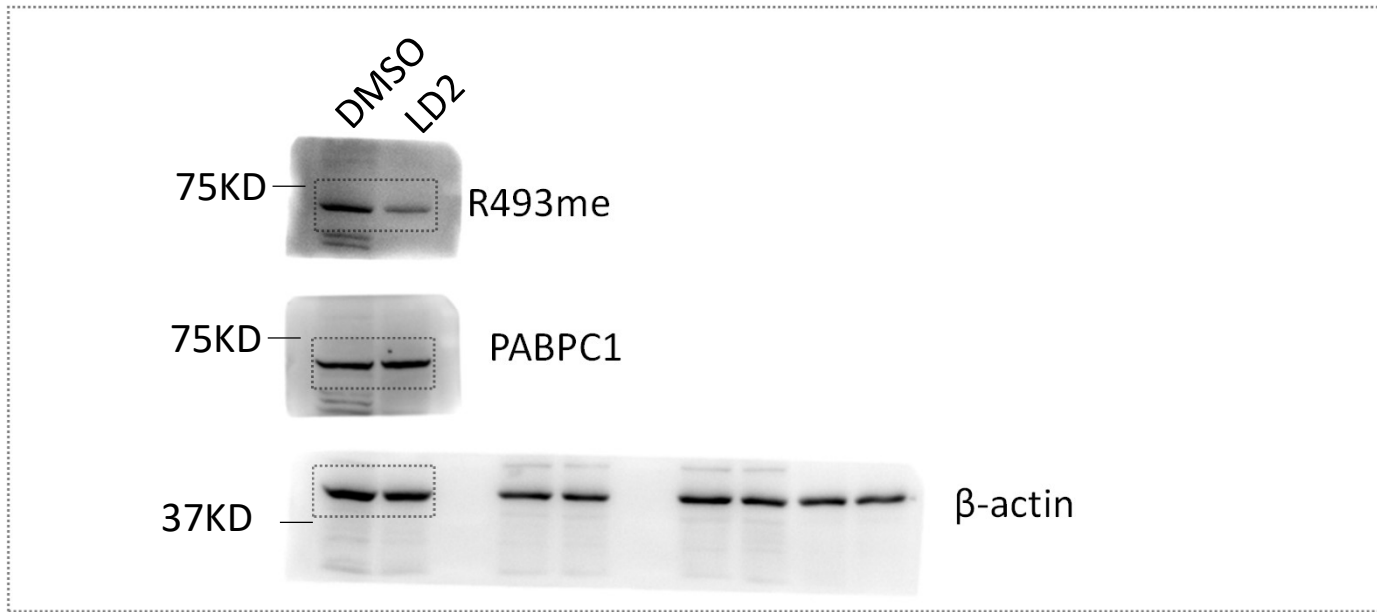

Supplement: Supplementary file 11 — Unprocessed immunoblots. [file 43018_2024_736_MOESM11_ESM.pdf]

Fig. 6 Unprocessed western blots

Fig. 6k

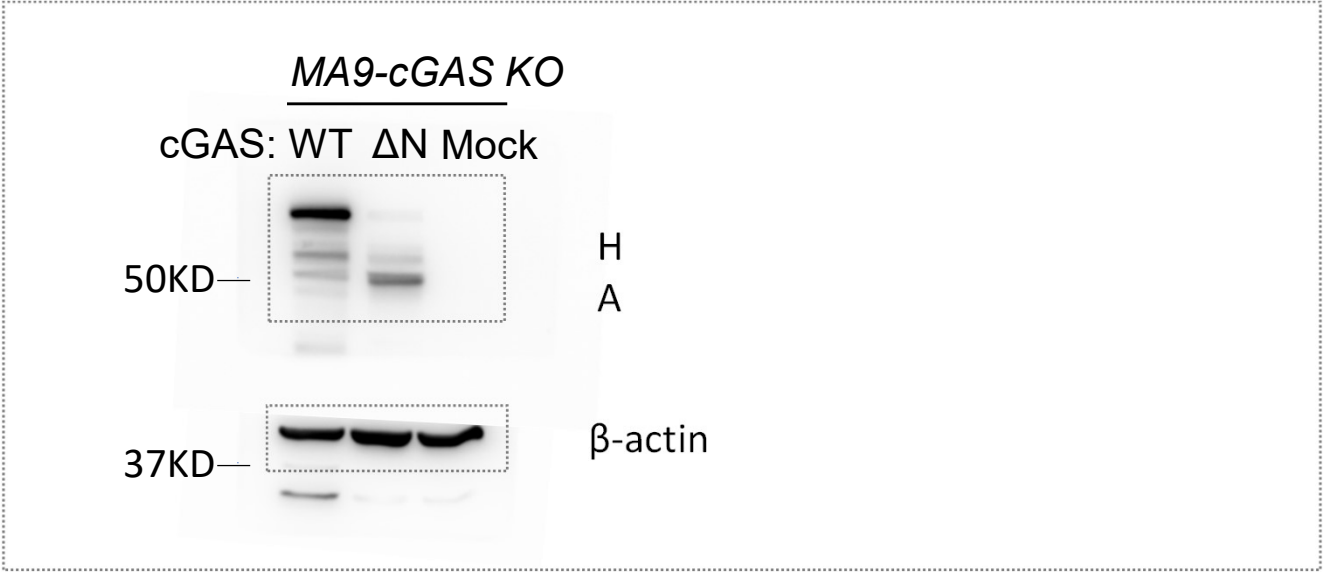

Supplement: Supplementary file 14 — Unprocessed immunoblots. [file 43018_2024_736_MOESM14_ESM.pdf]

# Extended Data Fig. 1 Unprocessed western blots

Extended Data Fig. 1p

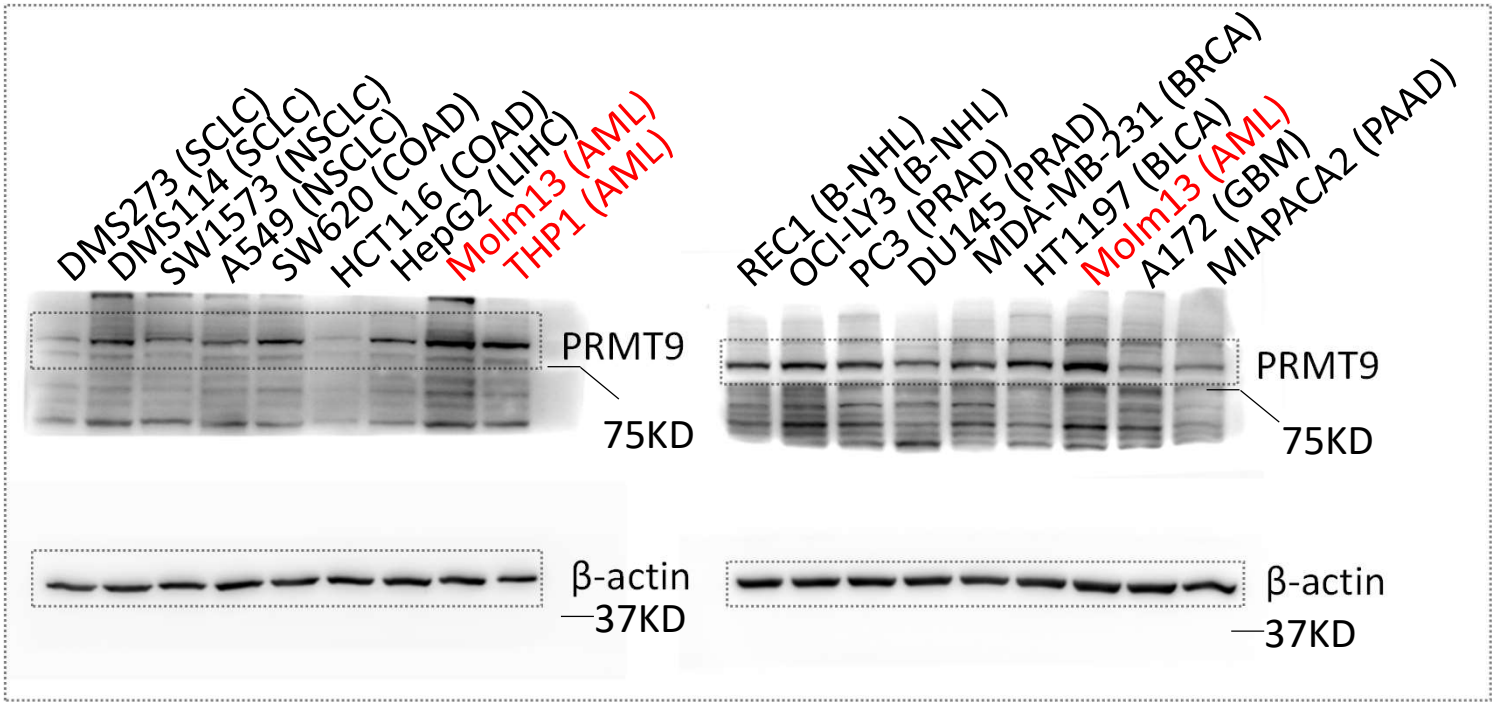

Supplement: Supplementary file 19 — Unprocessed immunoblots. [file 43018_2024_736_MOESM19_ESM.pdf]

Extended Data Fig. 5 Unprocessed western blots

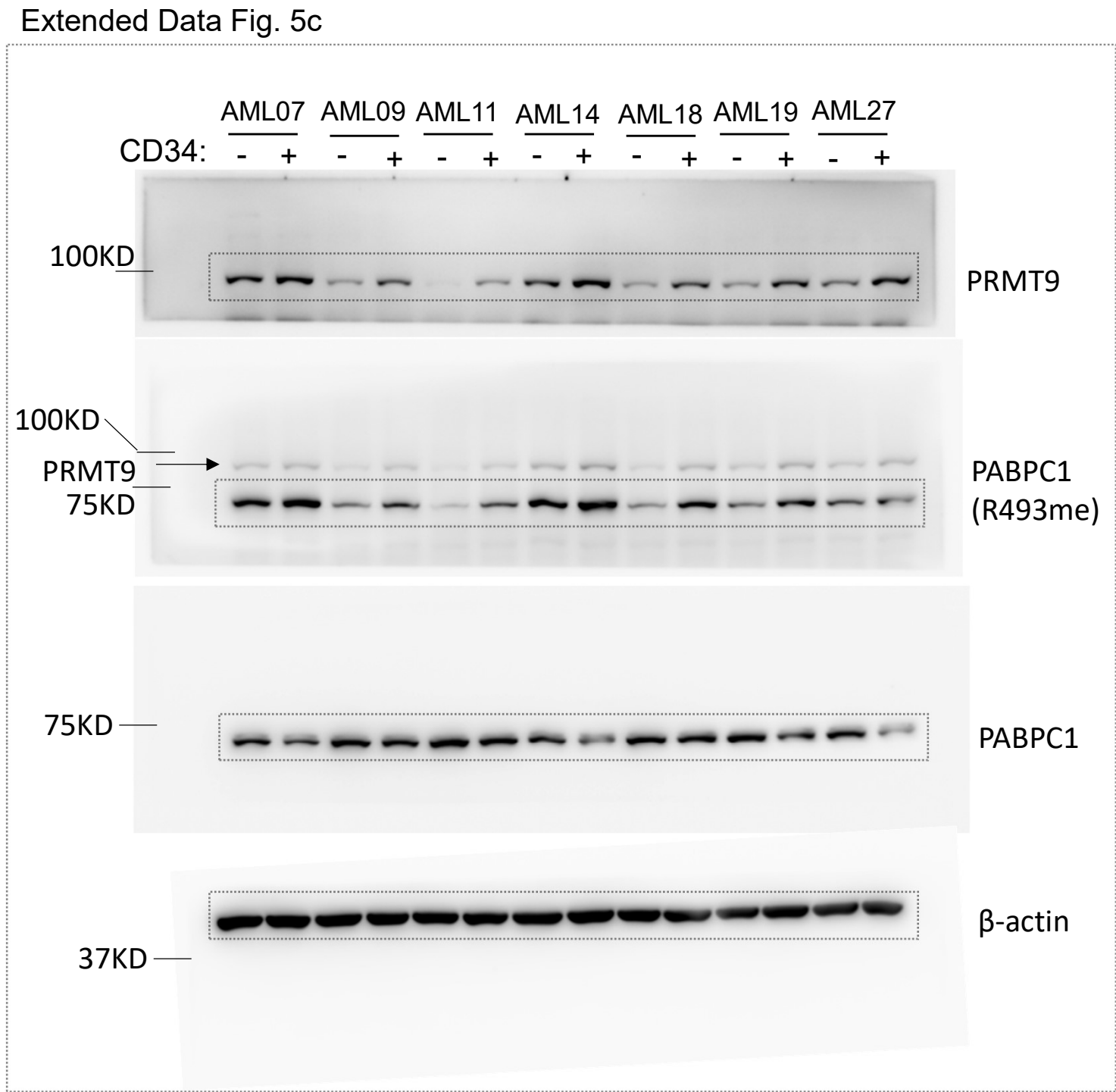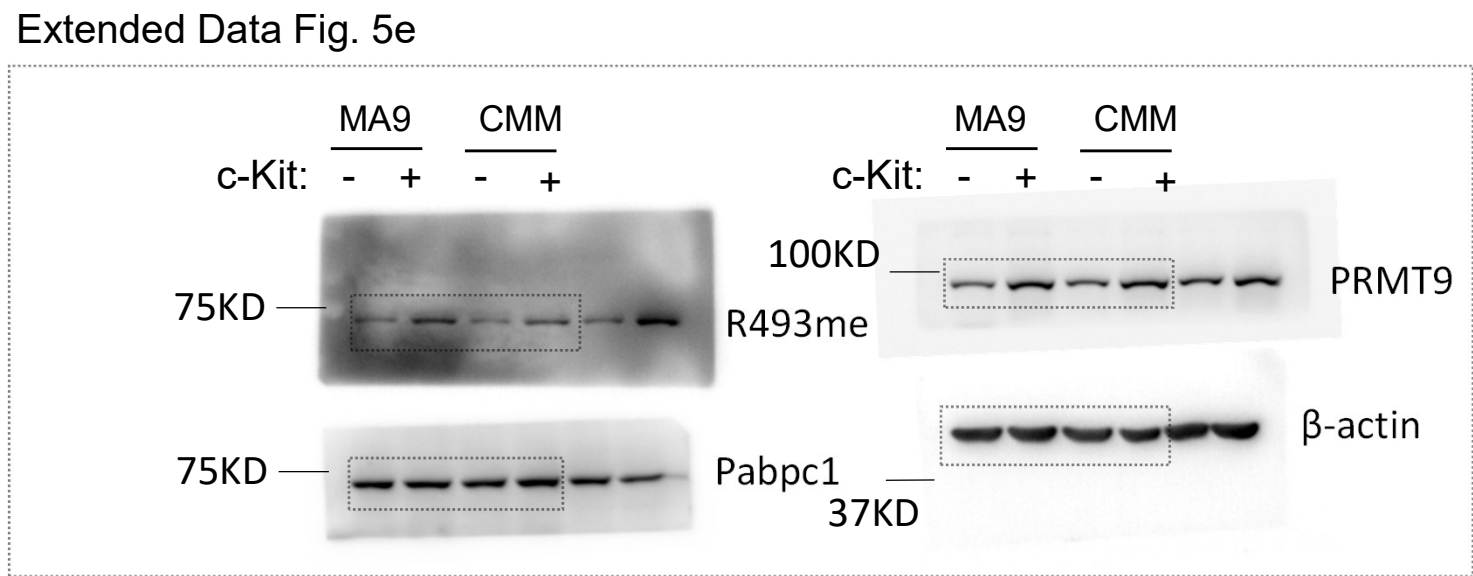

Supplement: Supplementary file 27 — Unprocessed immunoblots. [file 43018_2024_736_MOESM27_ESM.pdf]

# Extended Data Fig. 9 Unprocessed western blots

Extended Data Fig. 9f

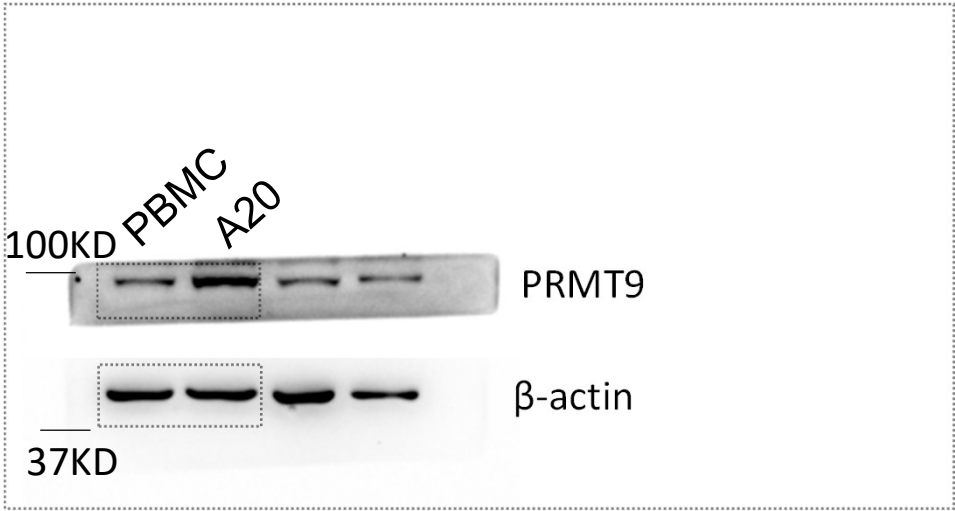

Extended Data Fig. 9l

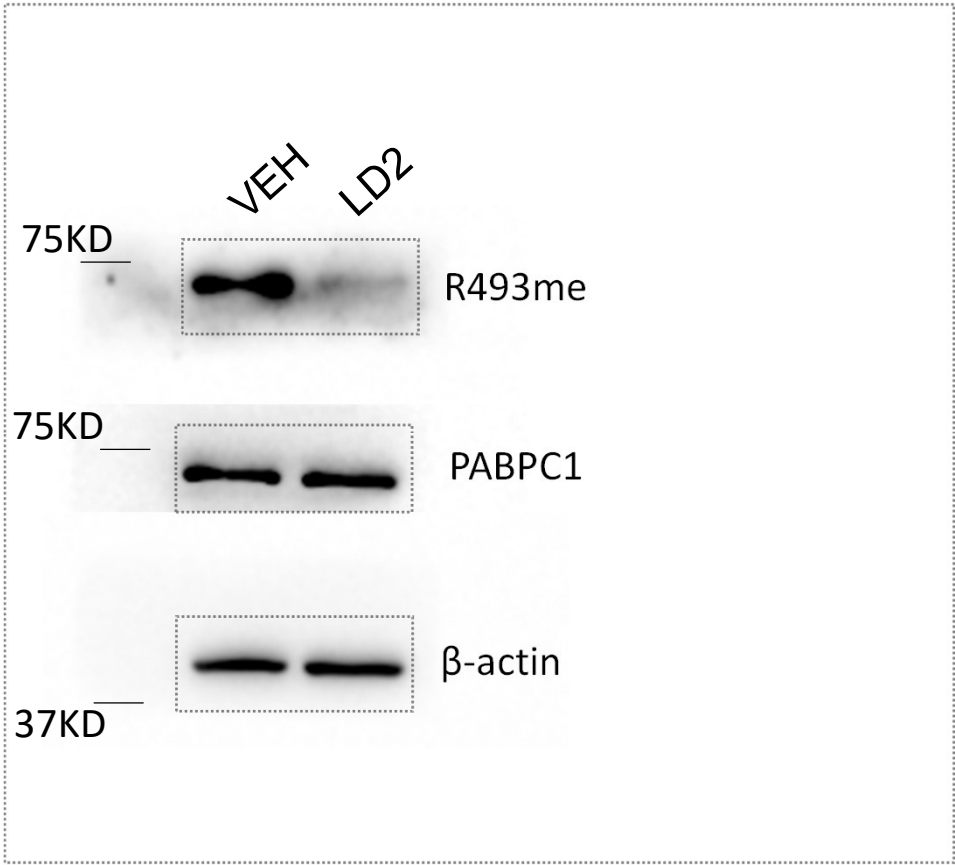

Supplement: Supplementary file 33 — Unprocessed immunoblots. [file 43018_2024_736_MOESM33_ESM.pdf]
