# Supplementary material for: Targeting PRMT9-mediated arginine methylation suppresses cancer stem cell maintenance and elicits cGAS-mediated anticancer immunity
Source: Nat Cancer. 2024 Feb 27;5(4):601–24. doi: 10.1038/s43018-024-00736-x (PMC11056319; doi:10.1038/s43018-024-00736-x)

Fig. 3 Unprocessed western blots

Fig.3g

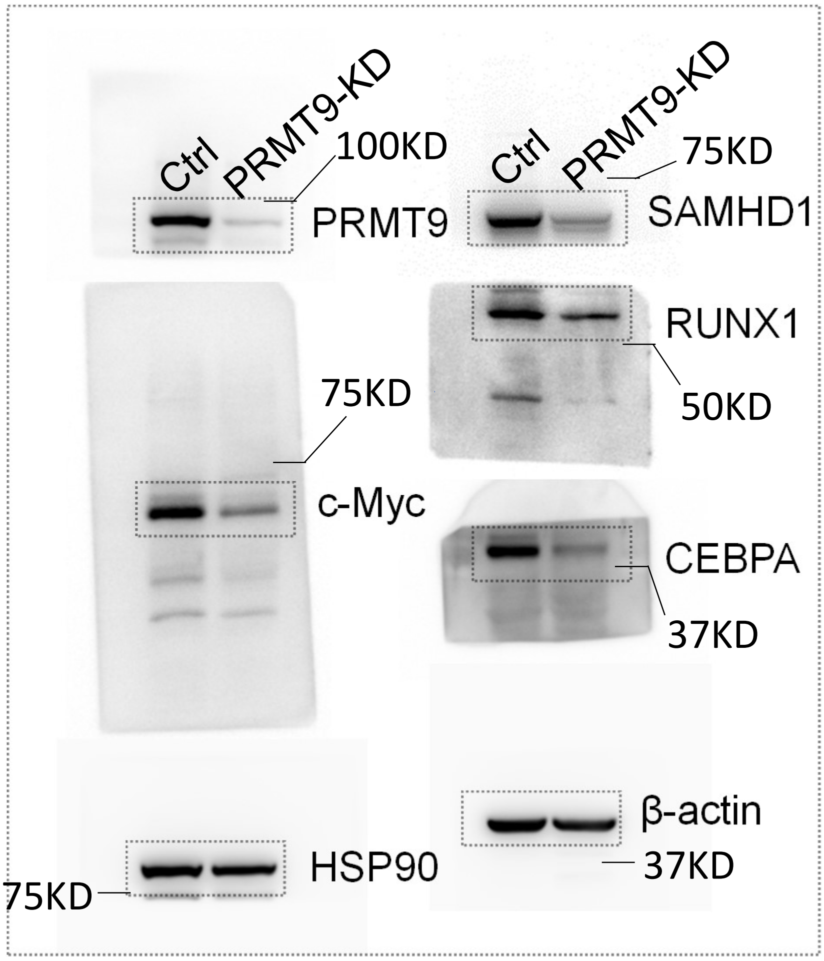

Fig.3k

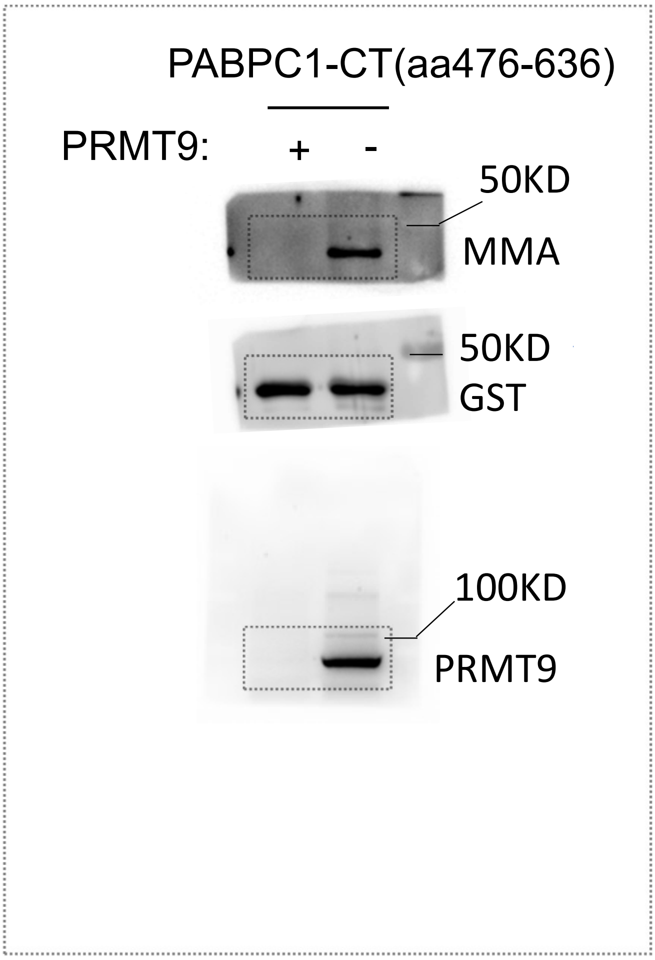

Fig.3l

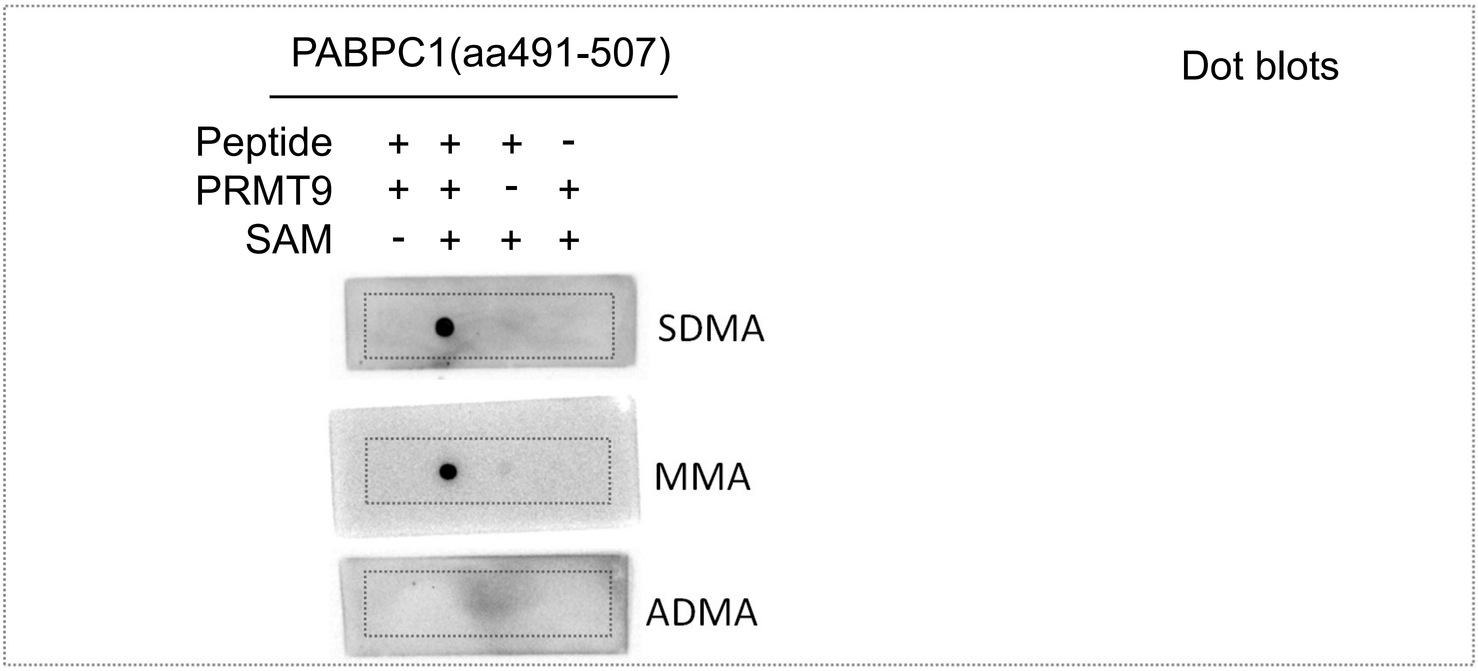

Fig. 3 Unprocessed western blots

Fig.3m

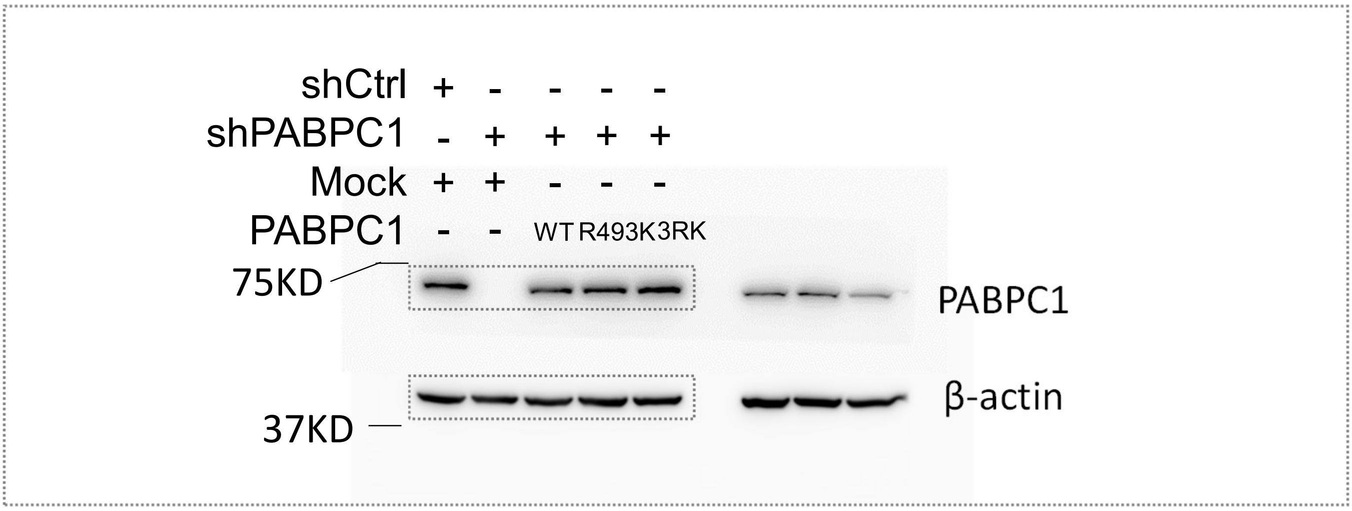

Fig.3q

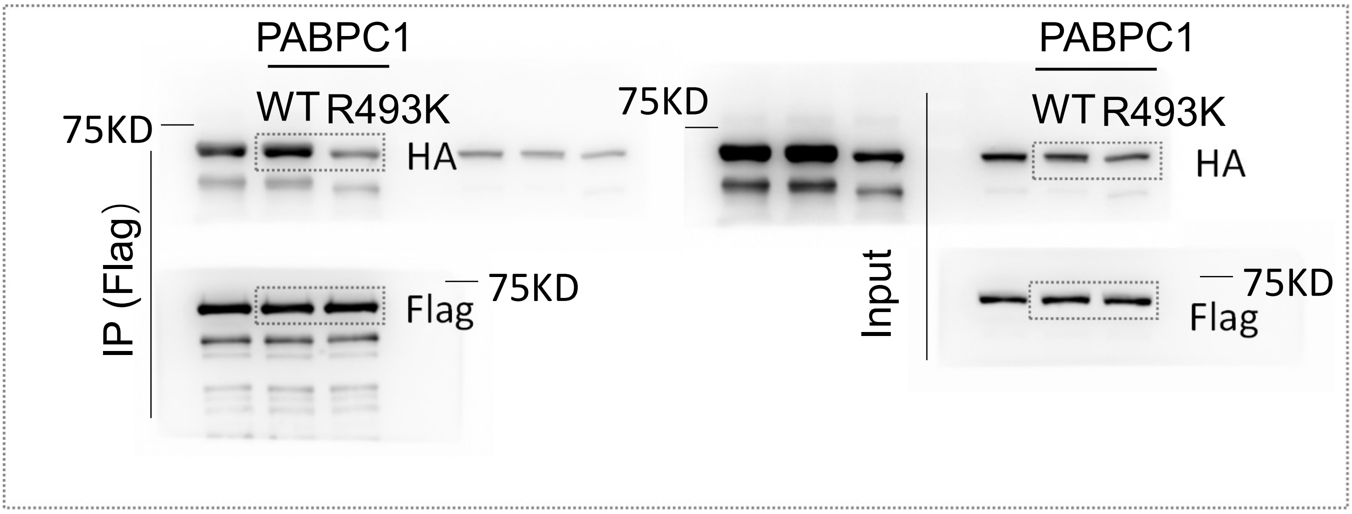

Fig.3r

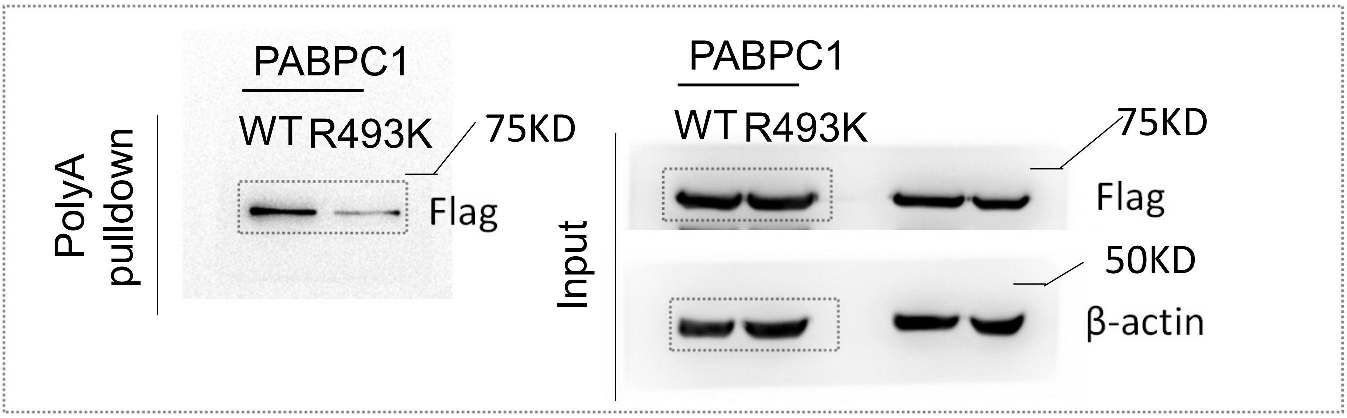

Fig. 3 Unprocessed western blots

Fig.3s

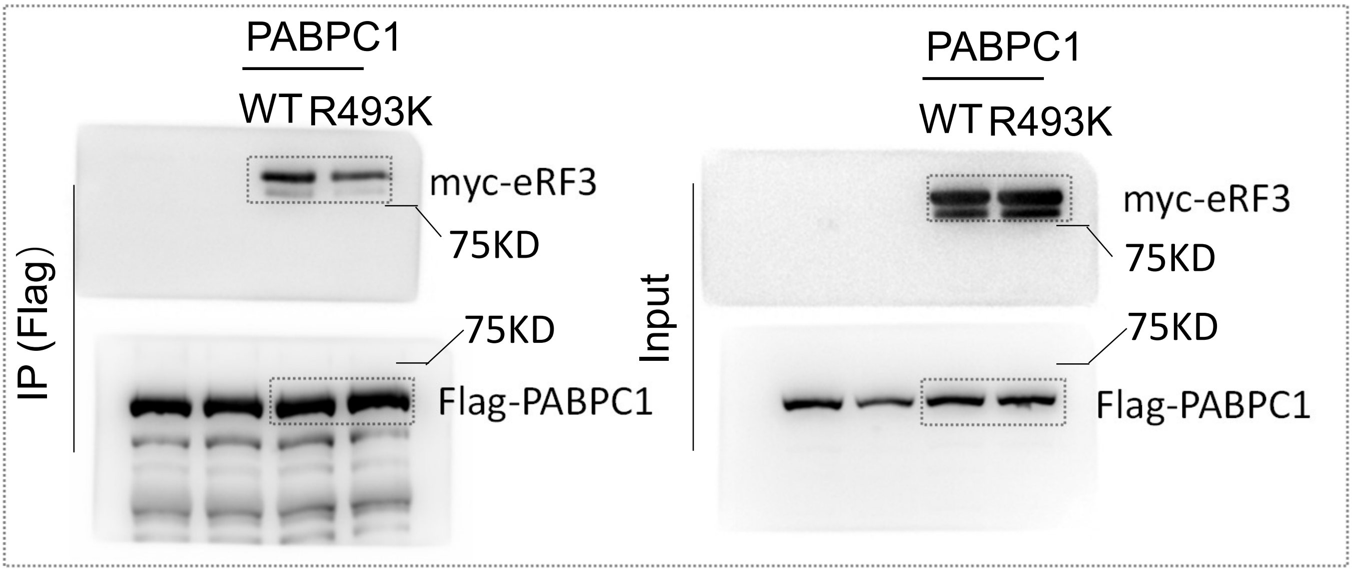

Fig.3t

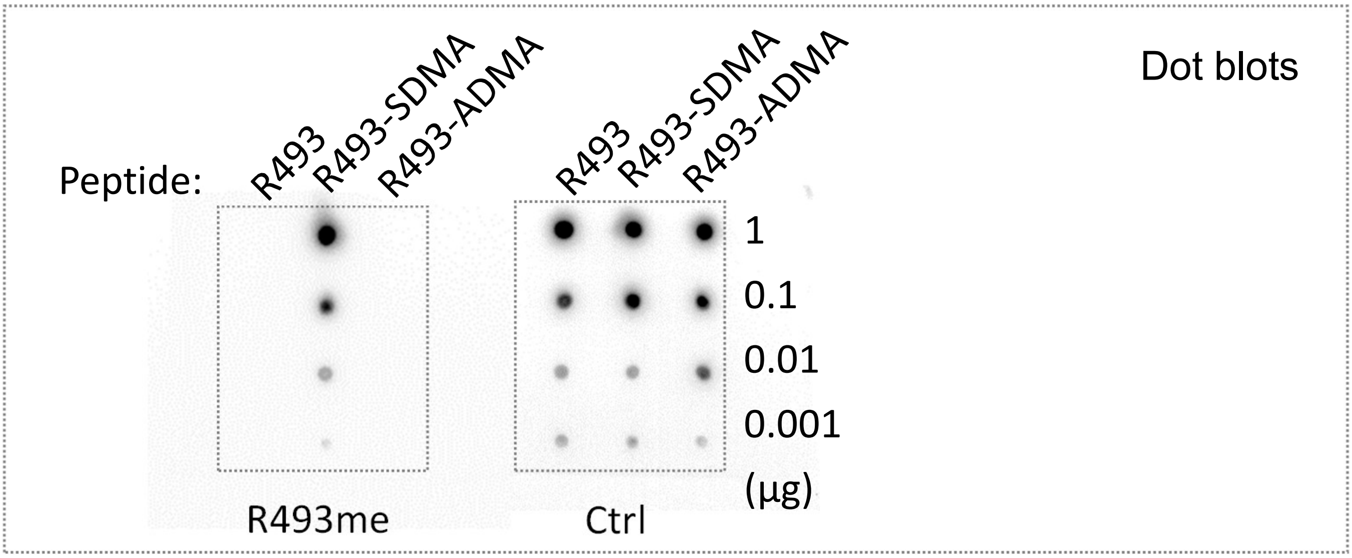

Fig.3u

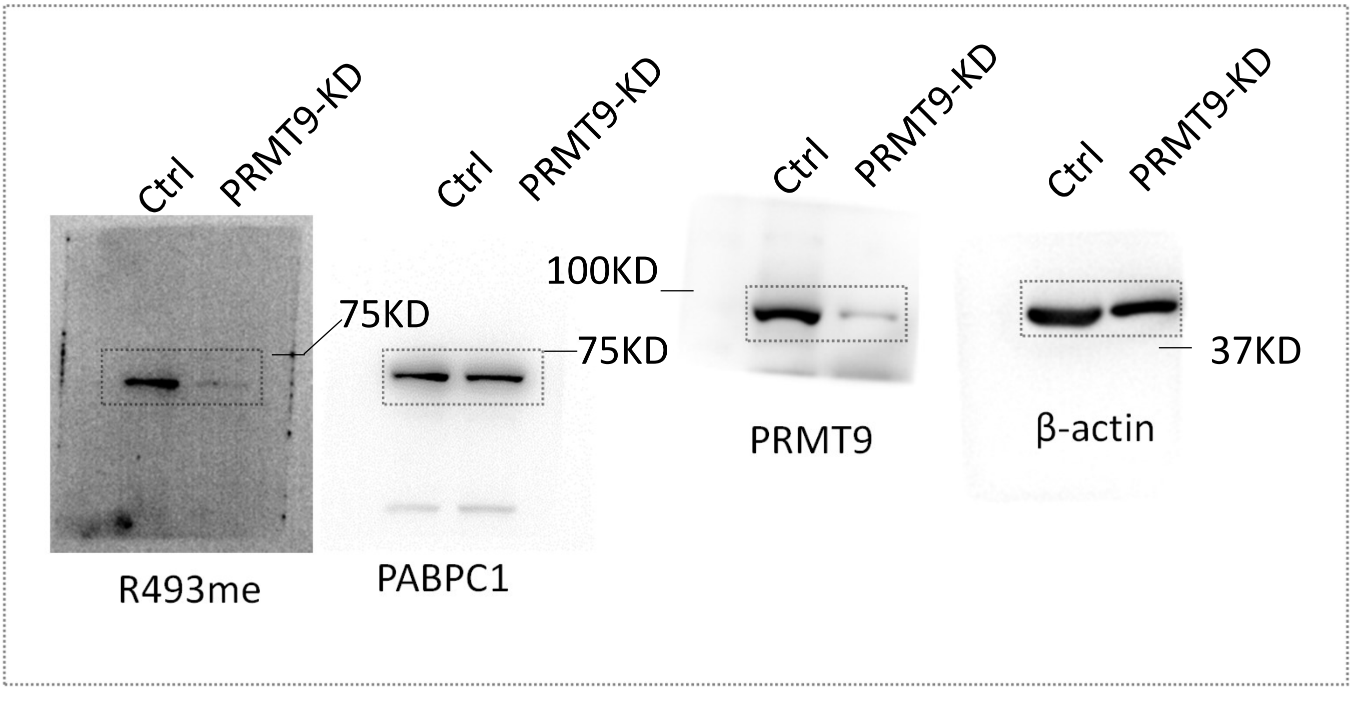

Supplement: Supplementary file 9 — Unprocessed immunoblots. [file 43018_2024_736_MOESM9_ESM.pdf]
