# Supplementary material for: Targeting PRMT9-mediated arginine methylation suppresses cancer stem cell maintenance and elicits cGAS-mediated anticancer immunity
Source: Nat Cancer. 2024 Feb 27;5(4):601–24. doi: 10.1038/s43018-024-00736-x (PMC11056319; doi:10.1038/s43018-024-00736-x)

Fig. 7 Unprocessed western blots

Fig. 7a

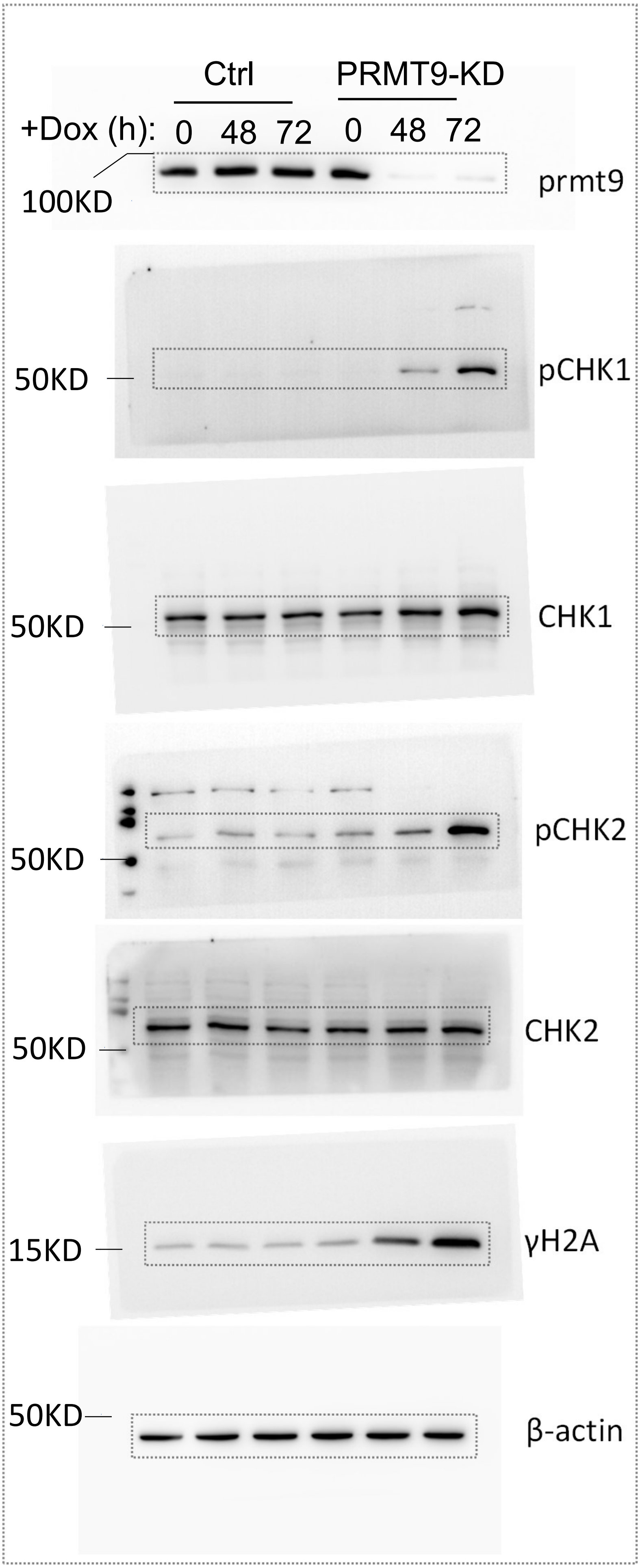

Fig. 7b

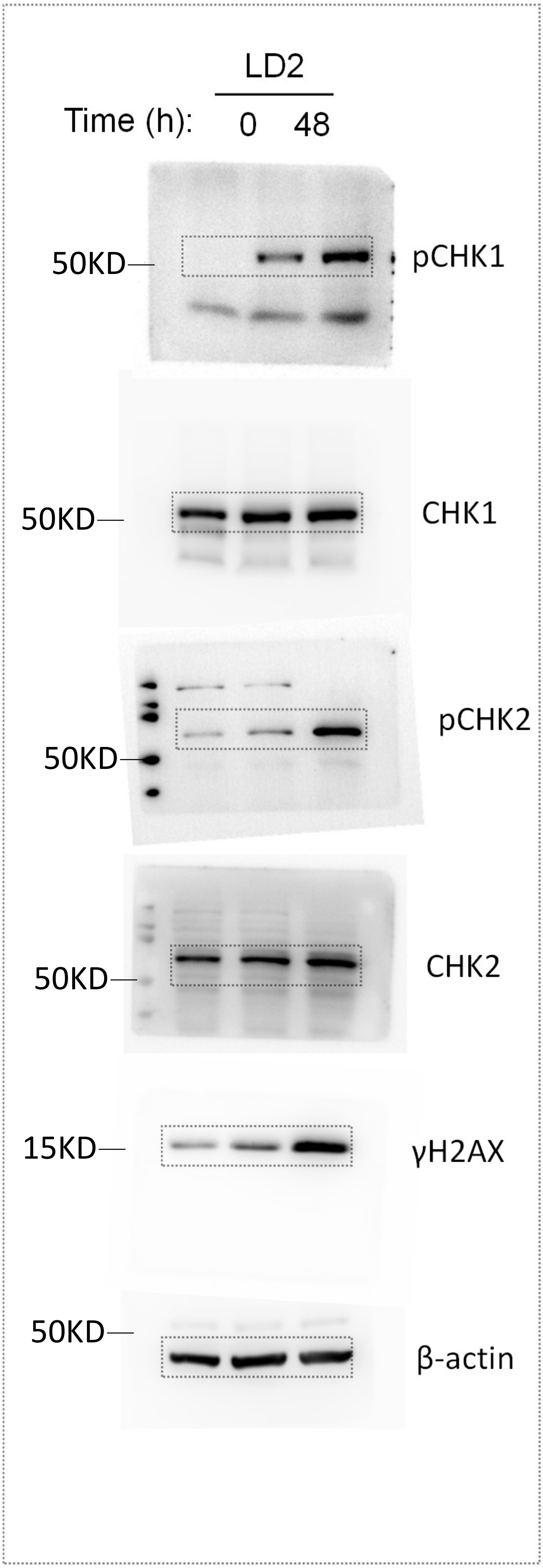

Fig. 7 Unprocessed western blots

Fig. 7e

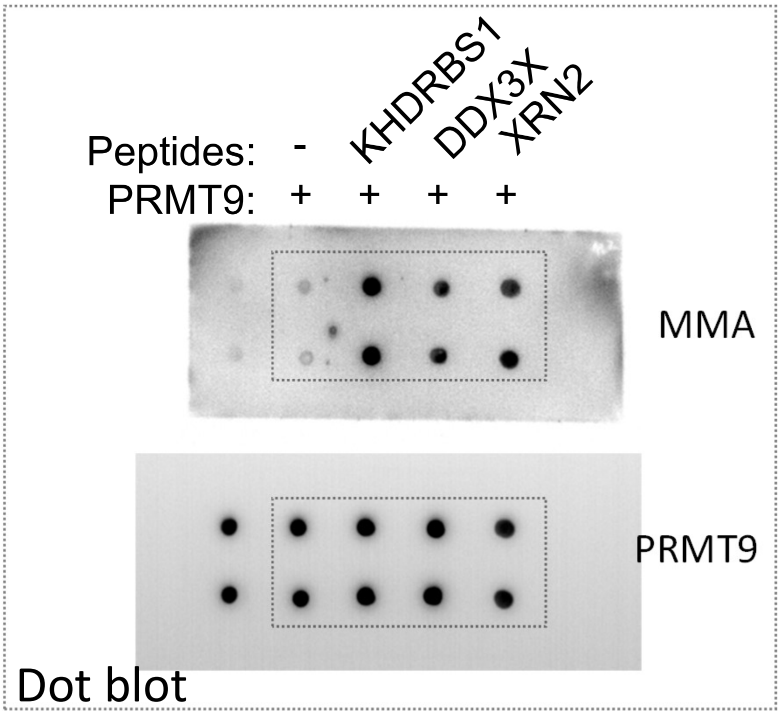

Fig. 7f

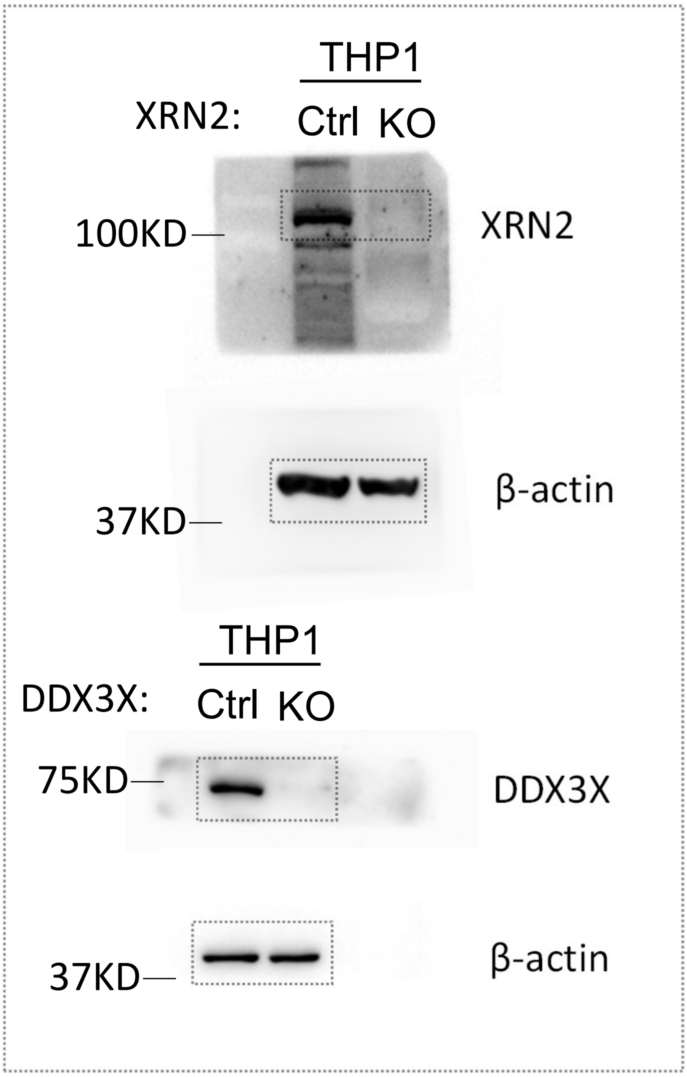

Fig. 7i

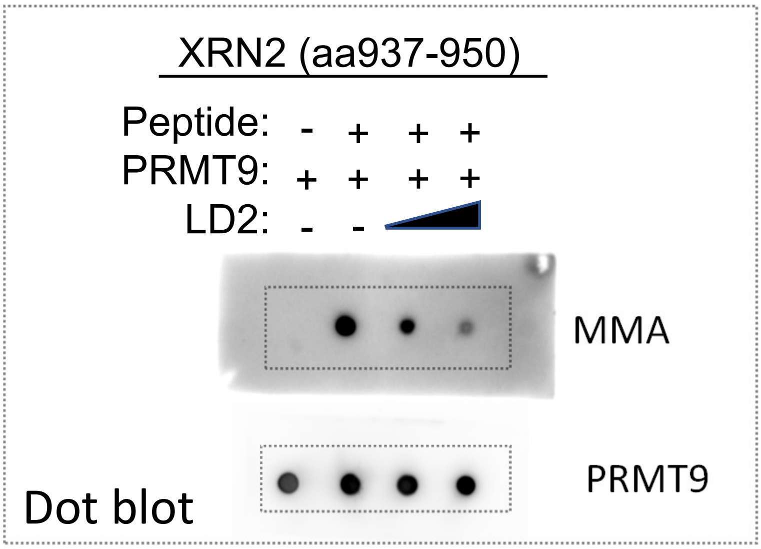

Fig. 7j

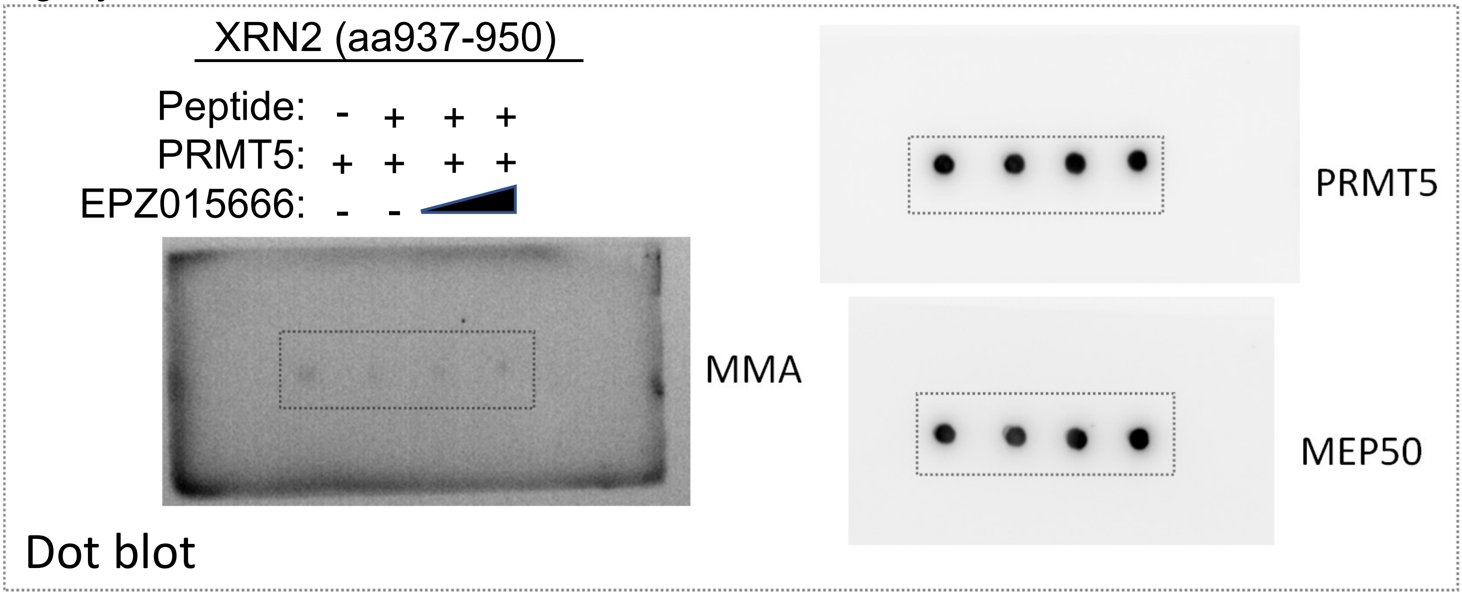

Fig. 7 Unprocessed western blots

Fig. 7k

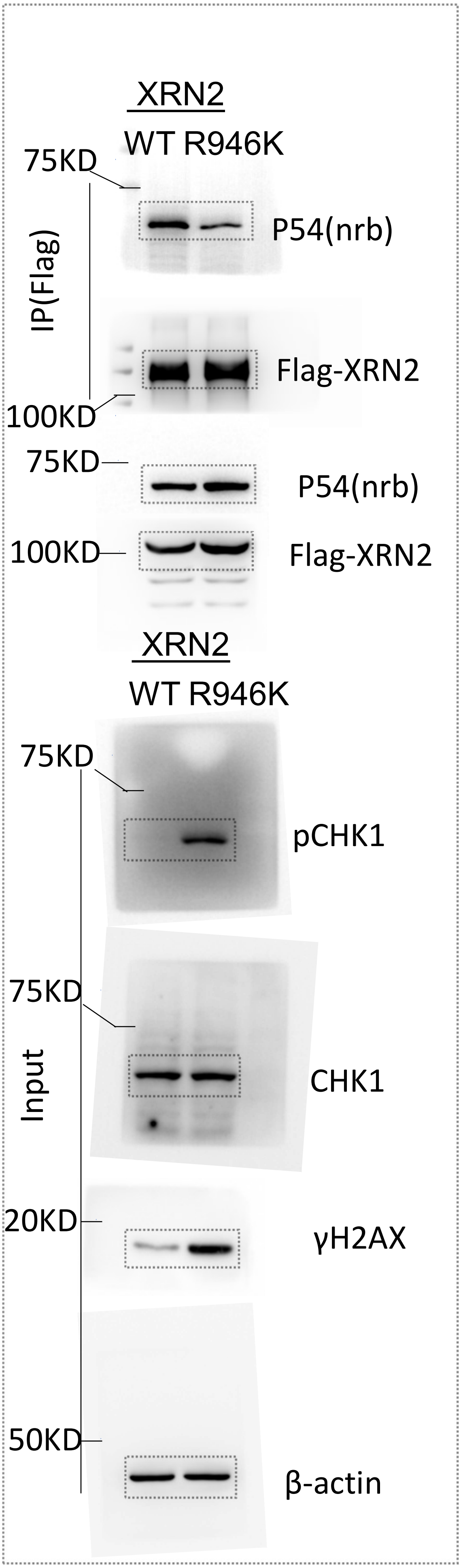

Fig. 7l

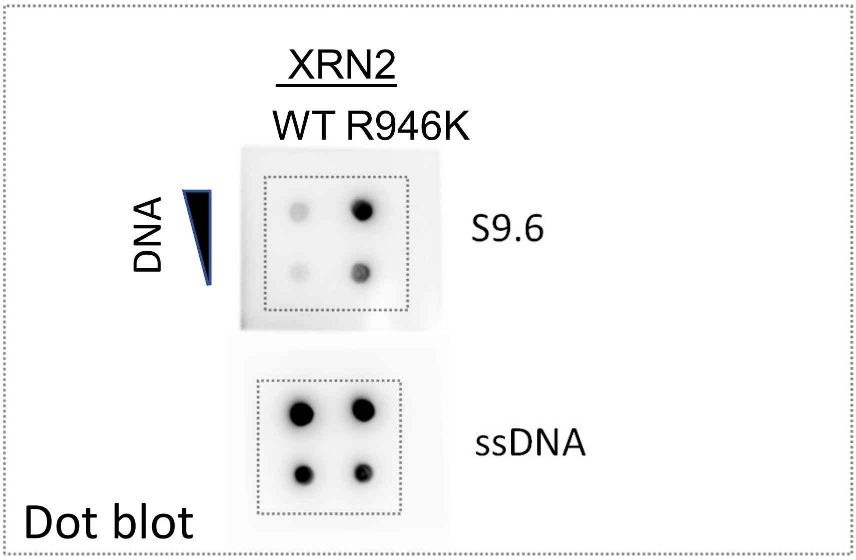

Fig. 7n

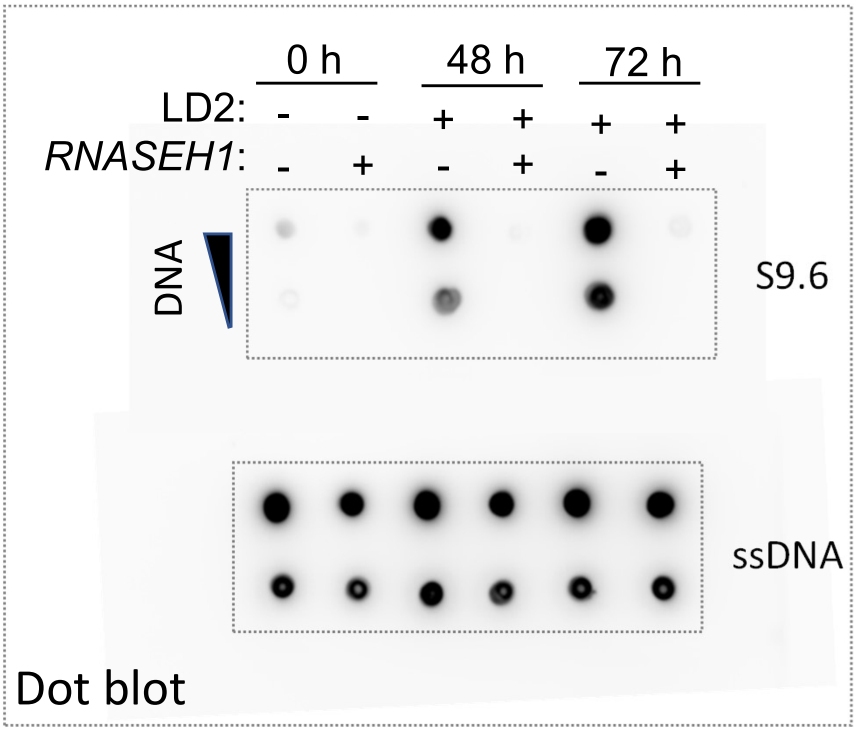

Fig. 7o

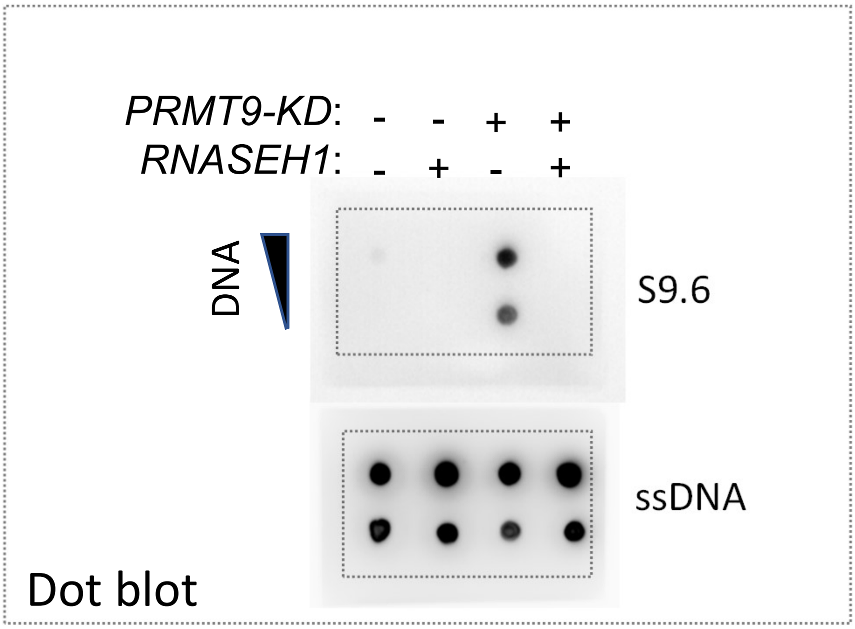

Fig. 7 Unprocessed western blots

Fig. 7q

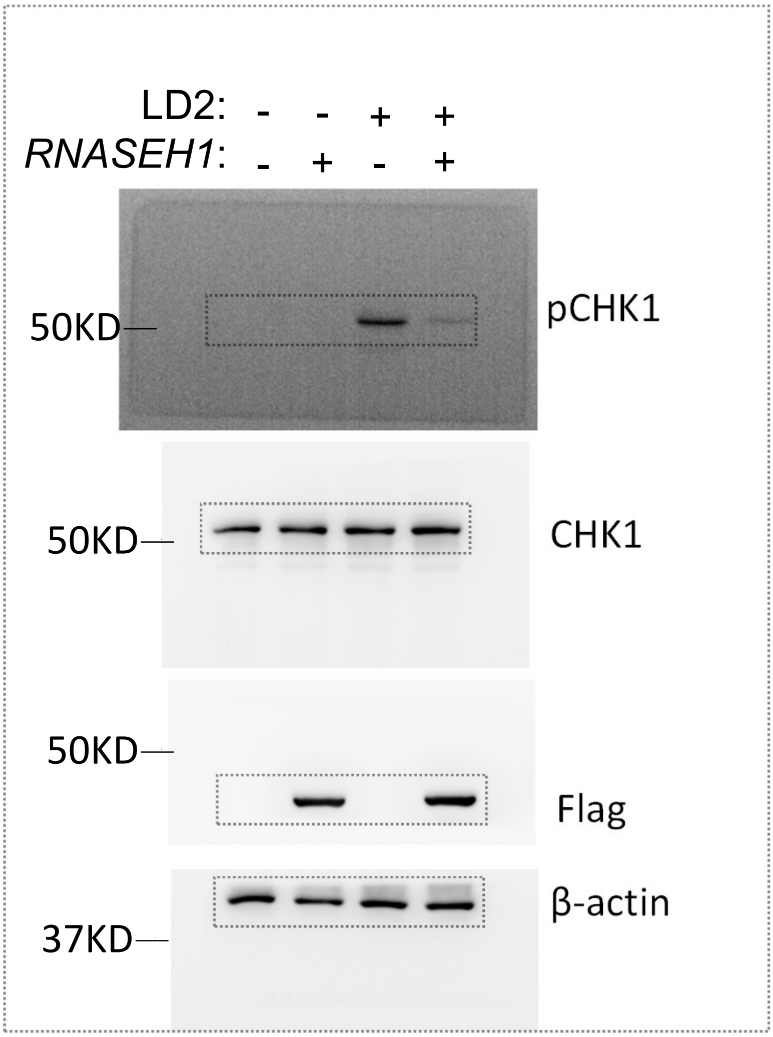

Supplement: Supplementary file 16 — Unprocessed immunoblots. [file 43018_2024_736_MOESM16_ESM.pdf]
