# Supplementary material for: Targeting PRMT9-mediated arginine methylation suppresses cancer stem cell maintenance and elicits cGAS-mediated anticancer immunity
Source: Nat Cancer. 2024 Feb 27;5(4):601–24. doi: 10.1038/s43018-024-00736-x (PMC11056319; doi:10.1038/s43018-024-00736-x)

Extended Data Fig. 3 Unprocessed western blots

Extended Data Fig. 3c

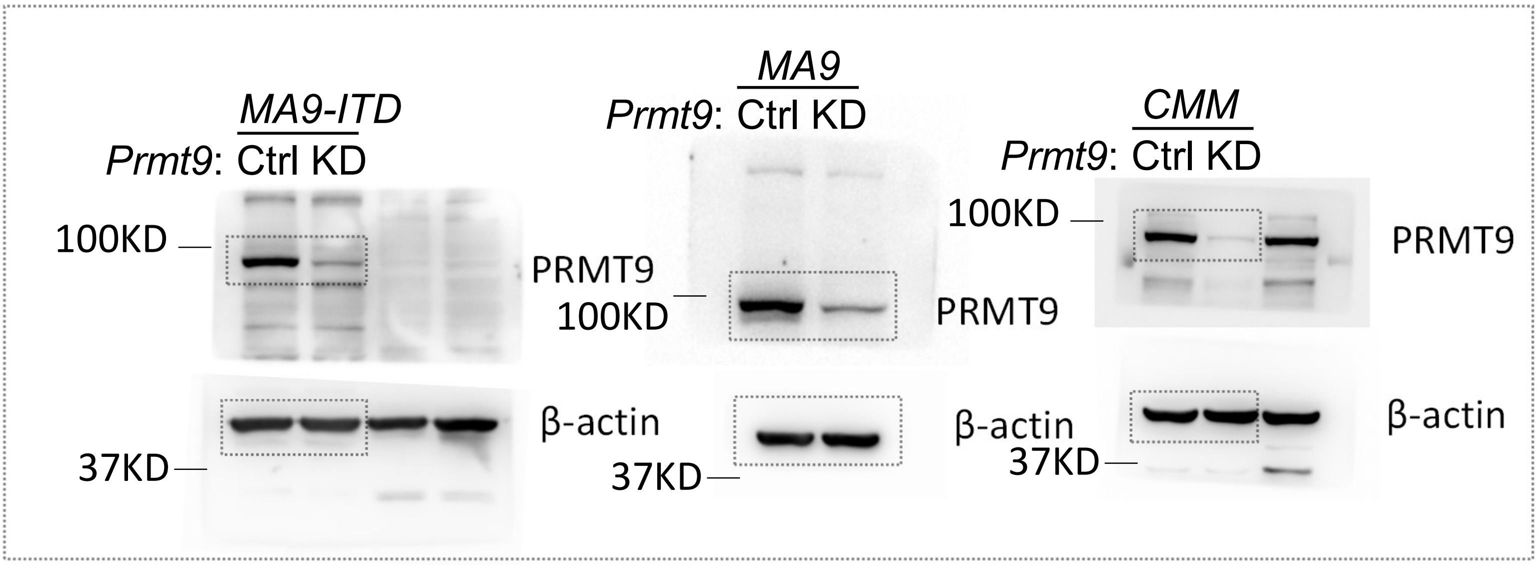

Extended Data Fig. 3h

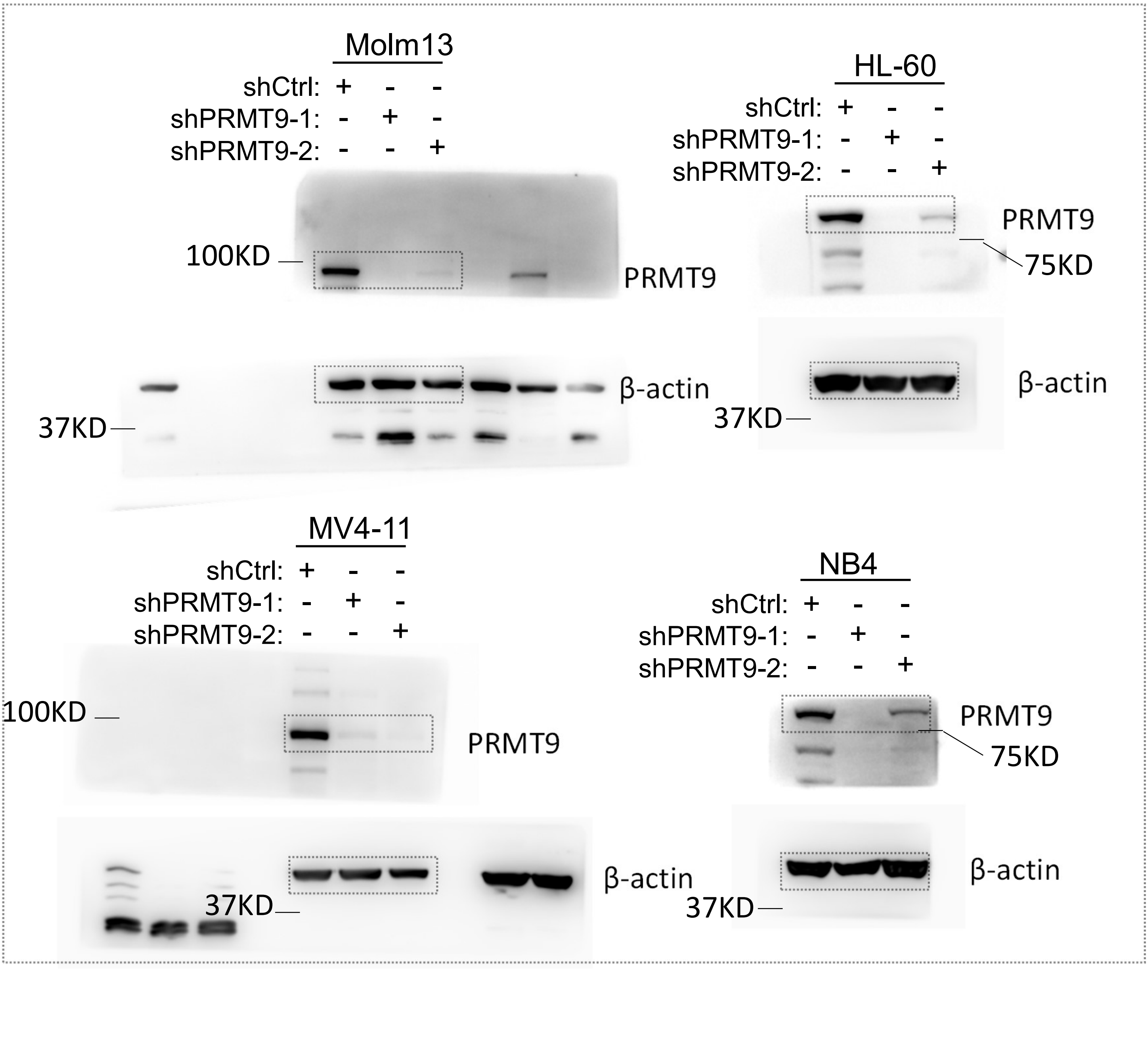

Extended Data Fig. 3 Unprocessed western blots

Extended Data Fig. 3i

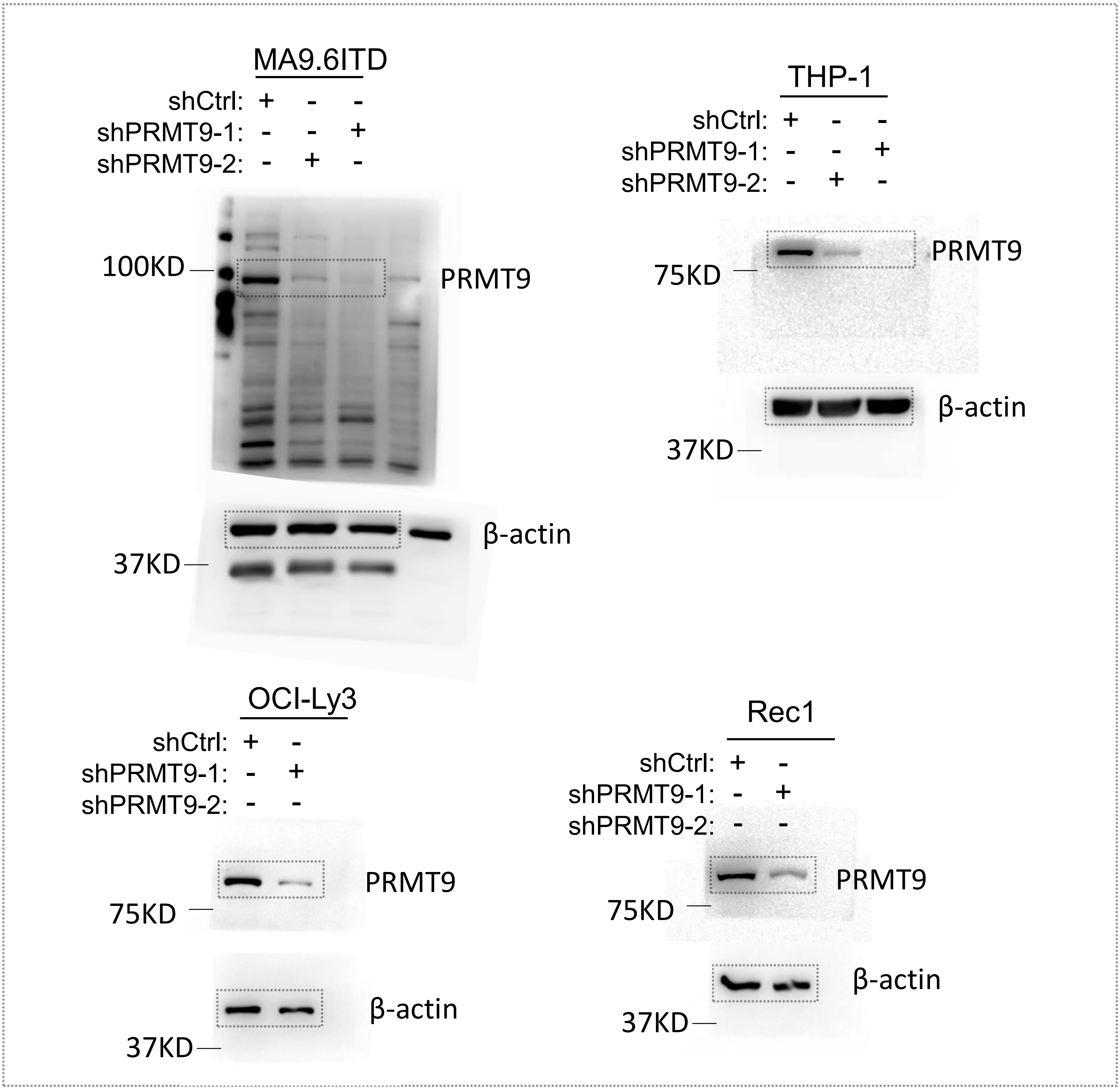

Supplement: Supplementary file 23 — Unprocessed immunoblots. [file 43018_2024_736_MOESM23_ESM.pdf]
