# Supplementary material for: Targeting PRMT9-mediated arginine methylation suppresses cancer stem cell maintenance and elicits cGAS-mediated anticancer immunity
Source: Nat Cancer. 2024 Feb 27;5(4):601–24. doi: 10.1038/s43018-024-00736-x (PMC11056319; doi:10.1038/s43018-024-00736-x)

Extended Data Fig. 4 Unprocessed western blots

Extended Data Fig. 4i

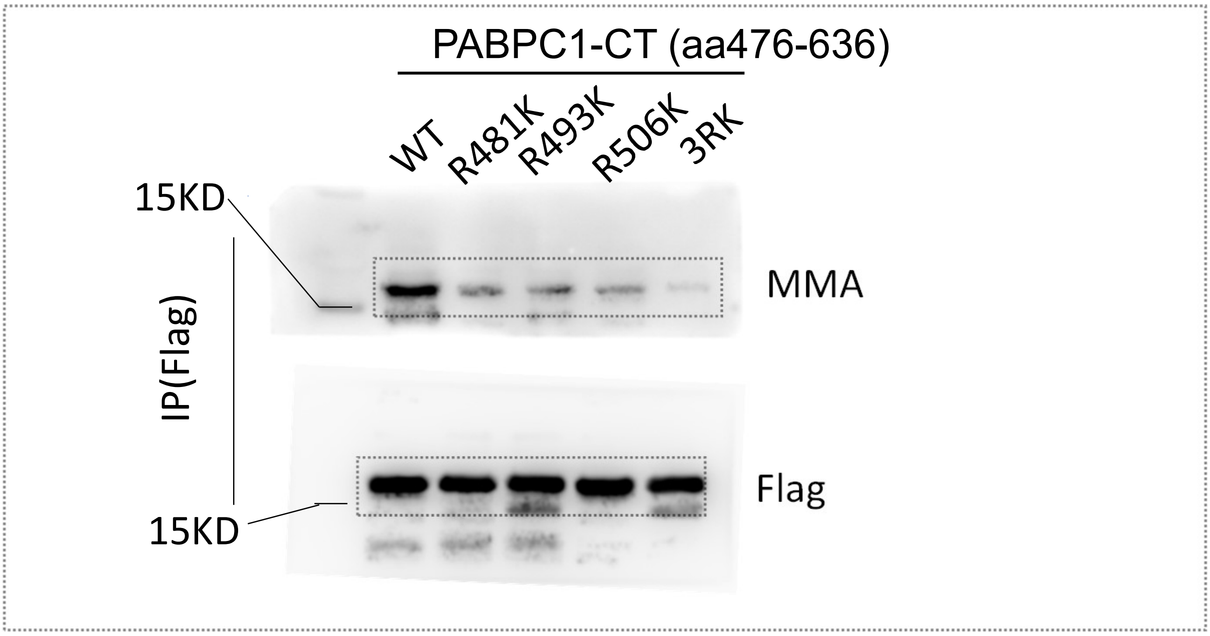

Extended Data Fig. 4j

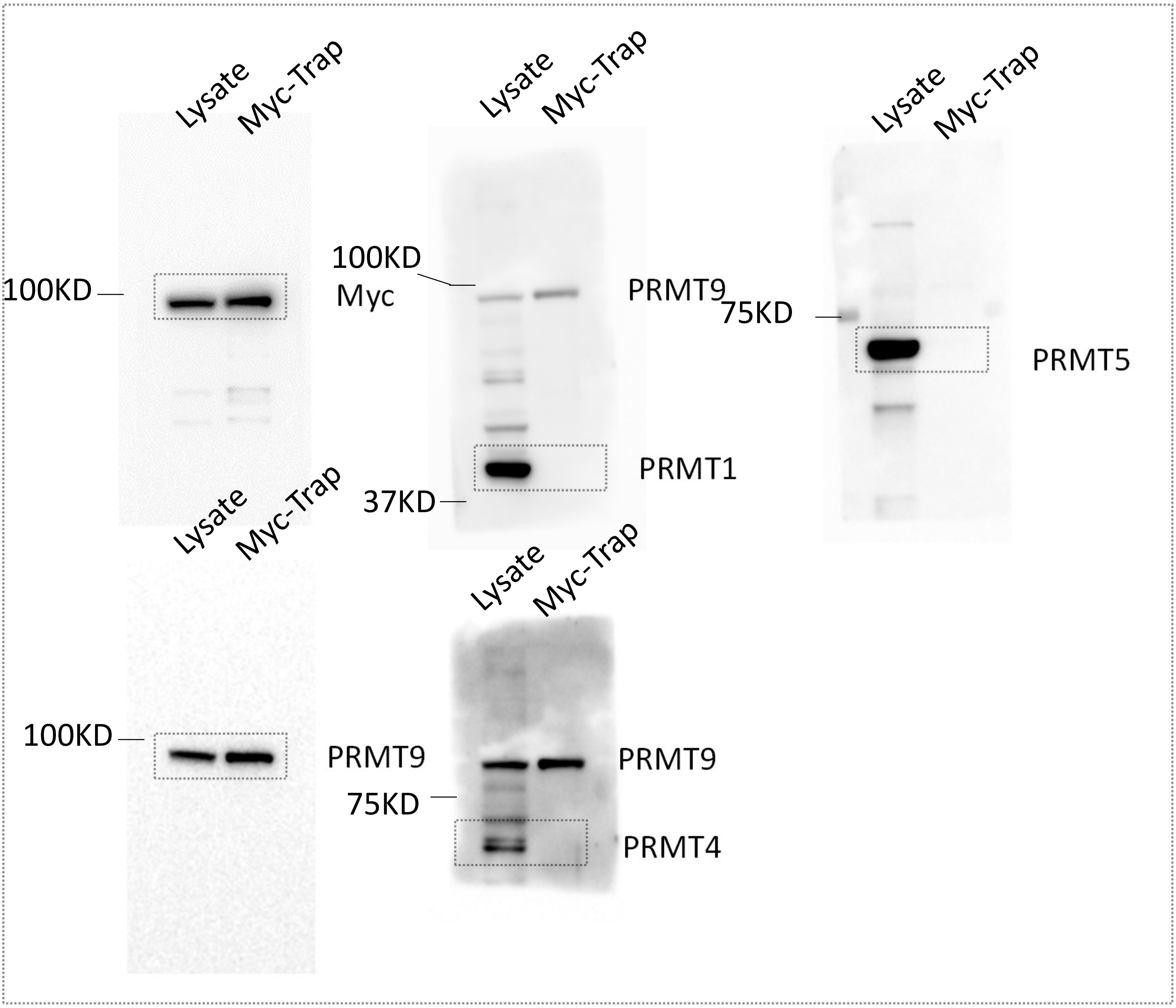

Extended Data Fig. 4 Unprocessed western blots

Extended Data Fig. 4k

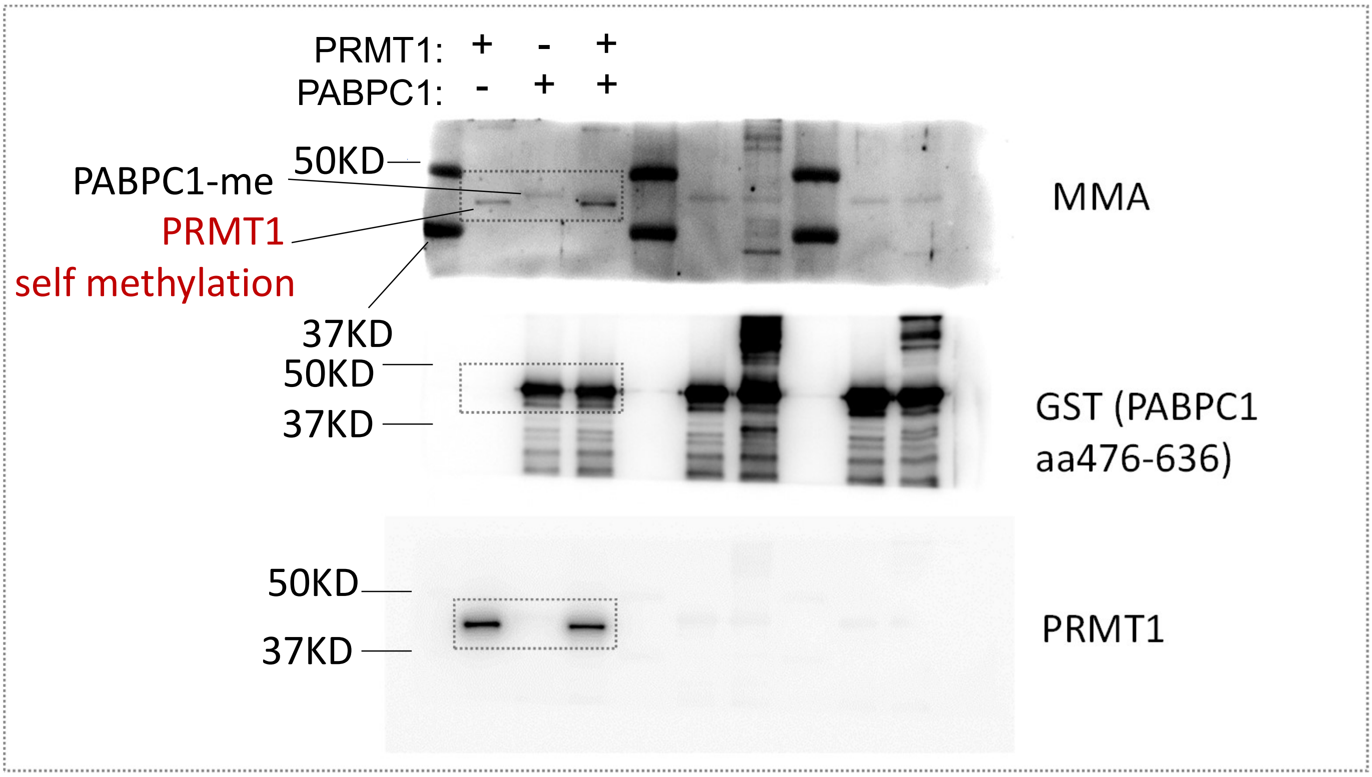

Extended Data Fig. 4l

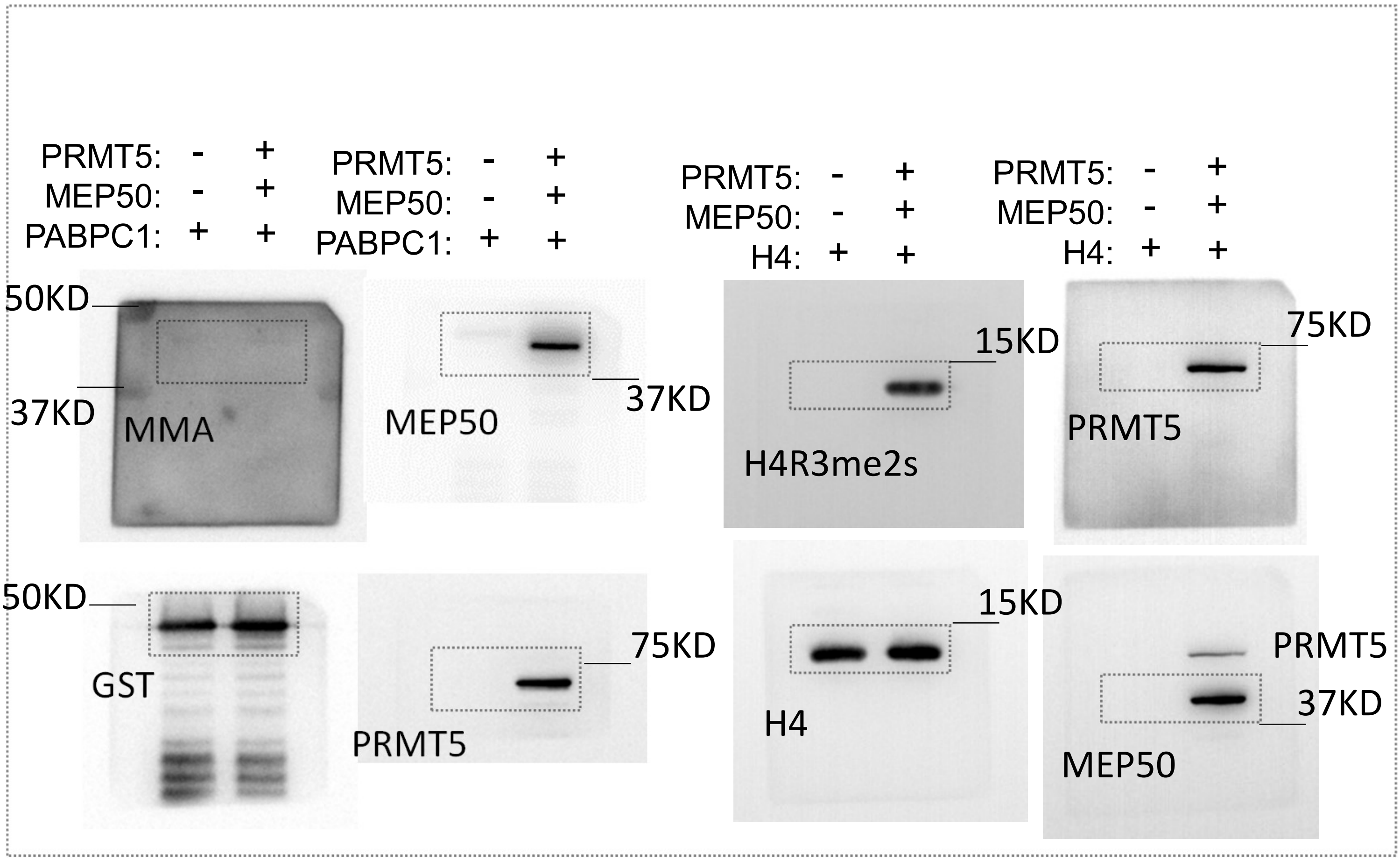

Extended Data Fig. 4 Unprocessed western blots

Extended Data Fig. 4m

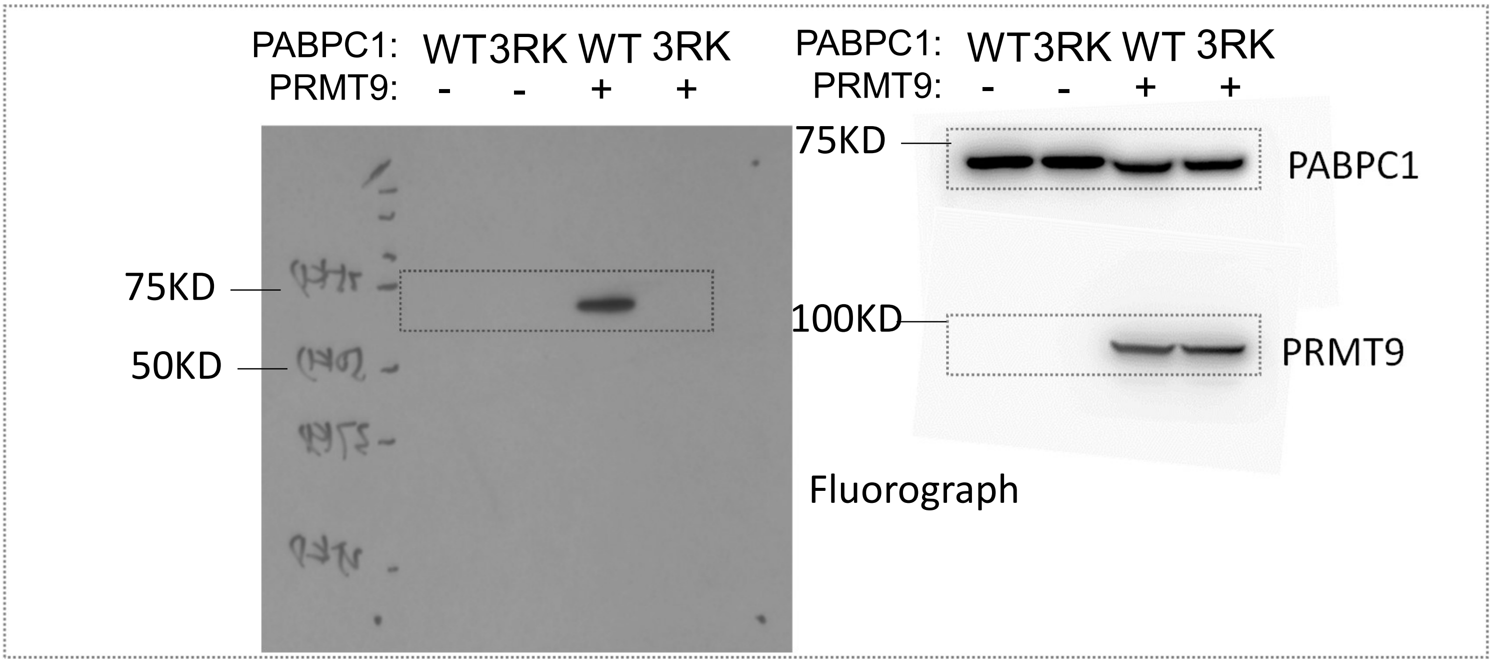

Extended Data Fig. 4n

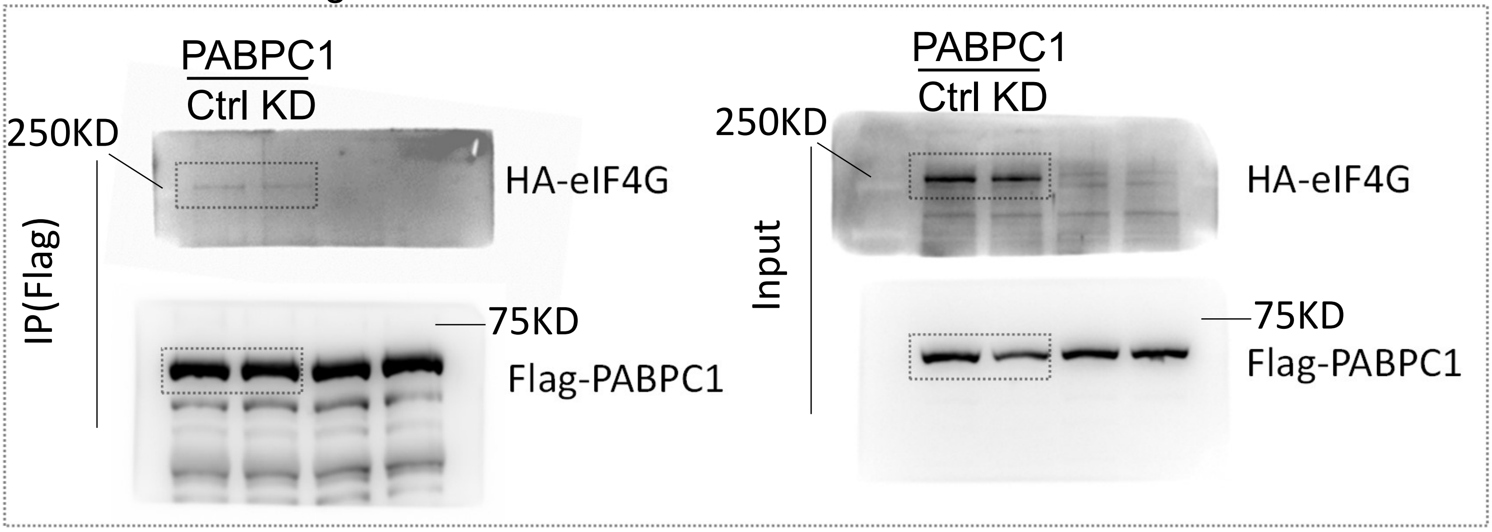

Extended Data Fig. 4o

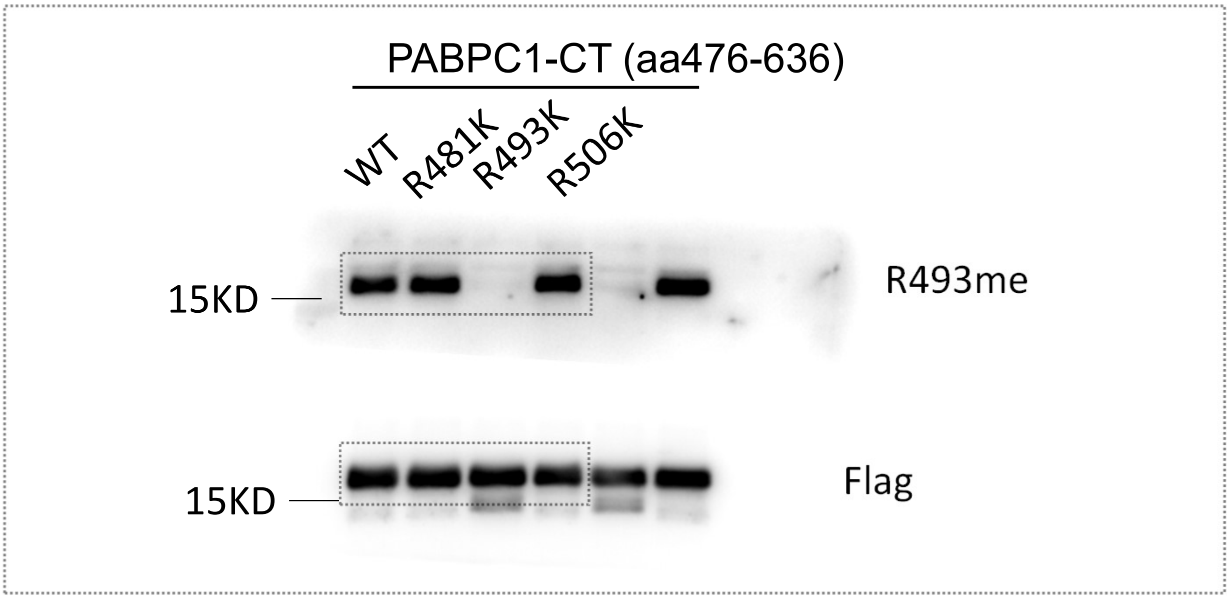

Extended Data Fig. 4 Unprocessed western blots

Extended Data Fig. 4p

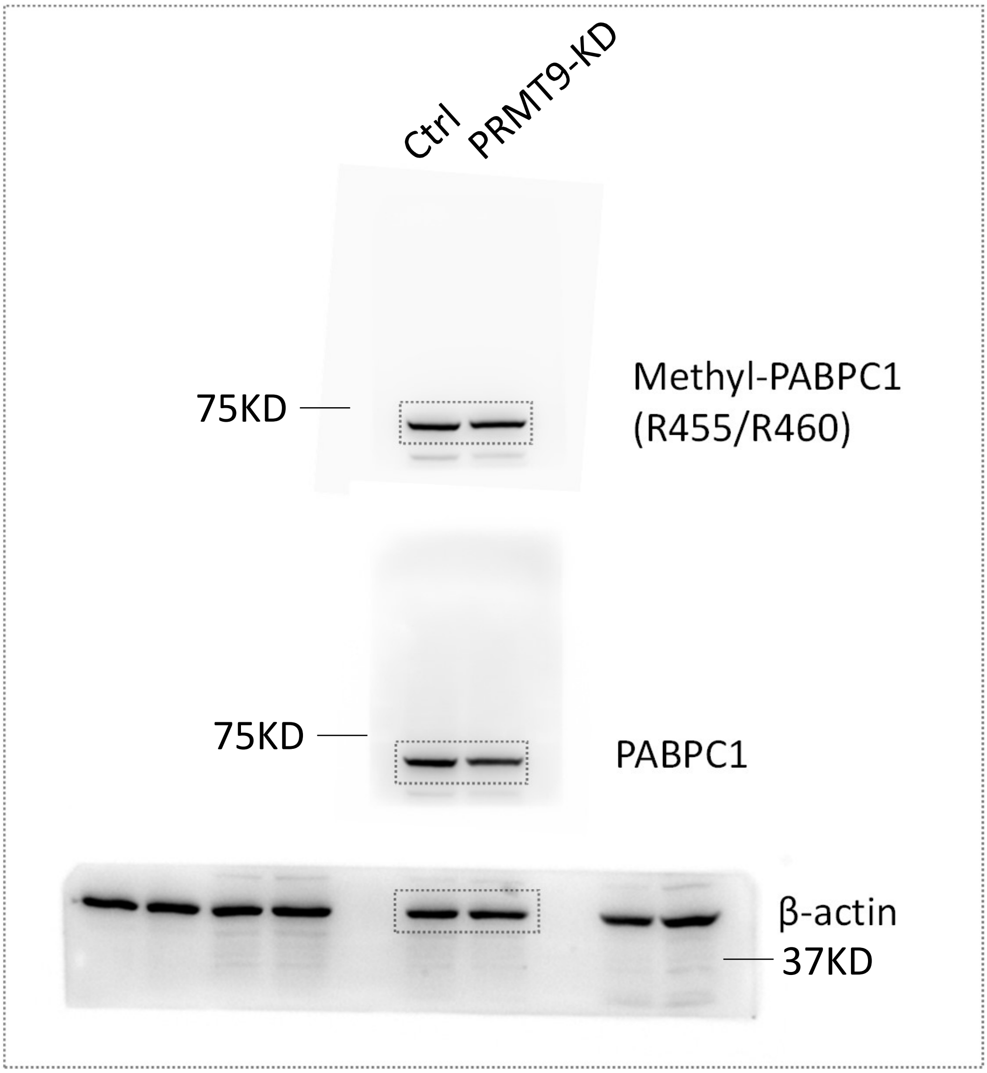

Extended Data Fig. 4q

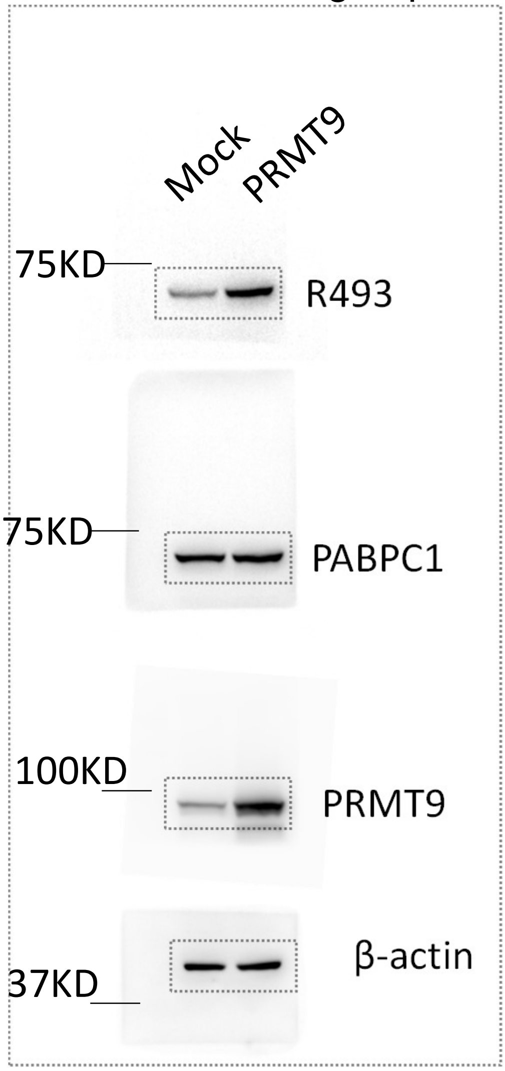

Extended Data Fig. 4r

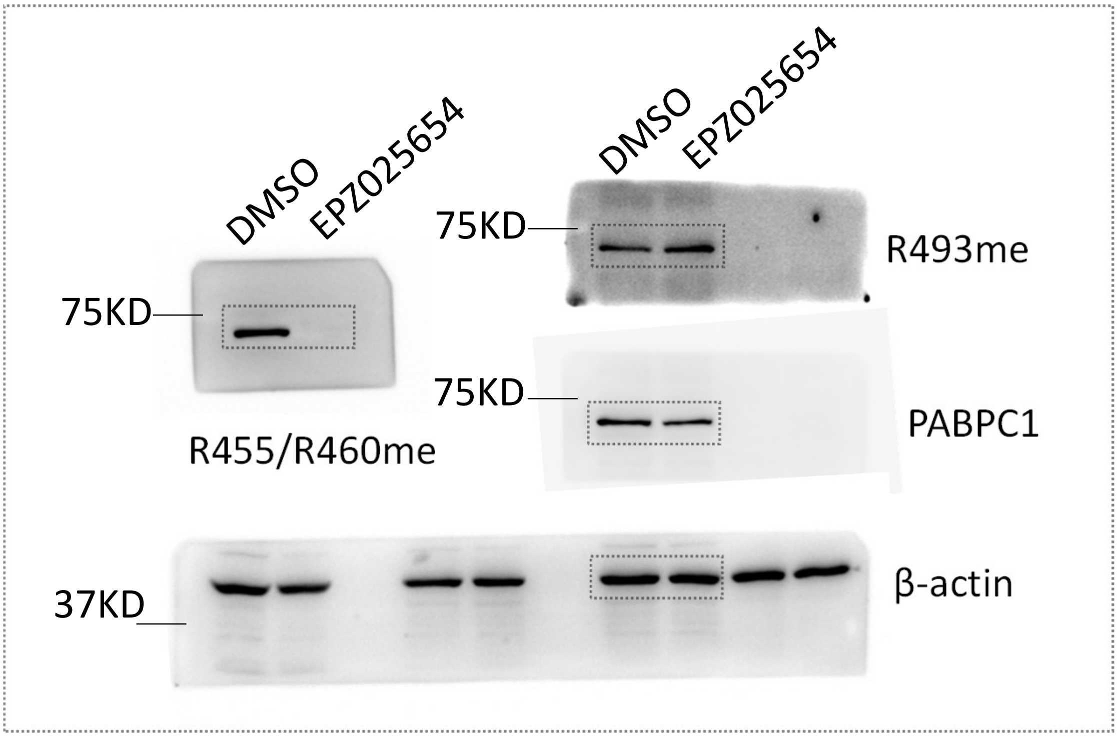

Extended Data Fig. 4 Unprocessed western blots

Extended Data Fig. 4s

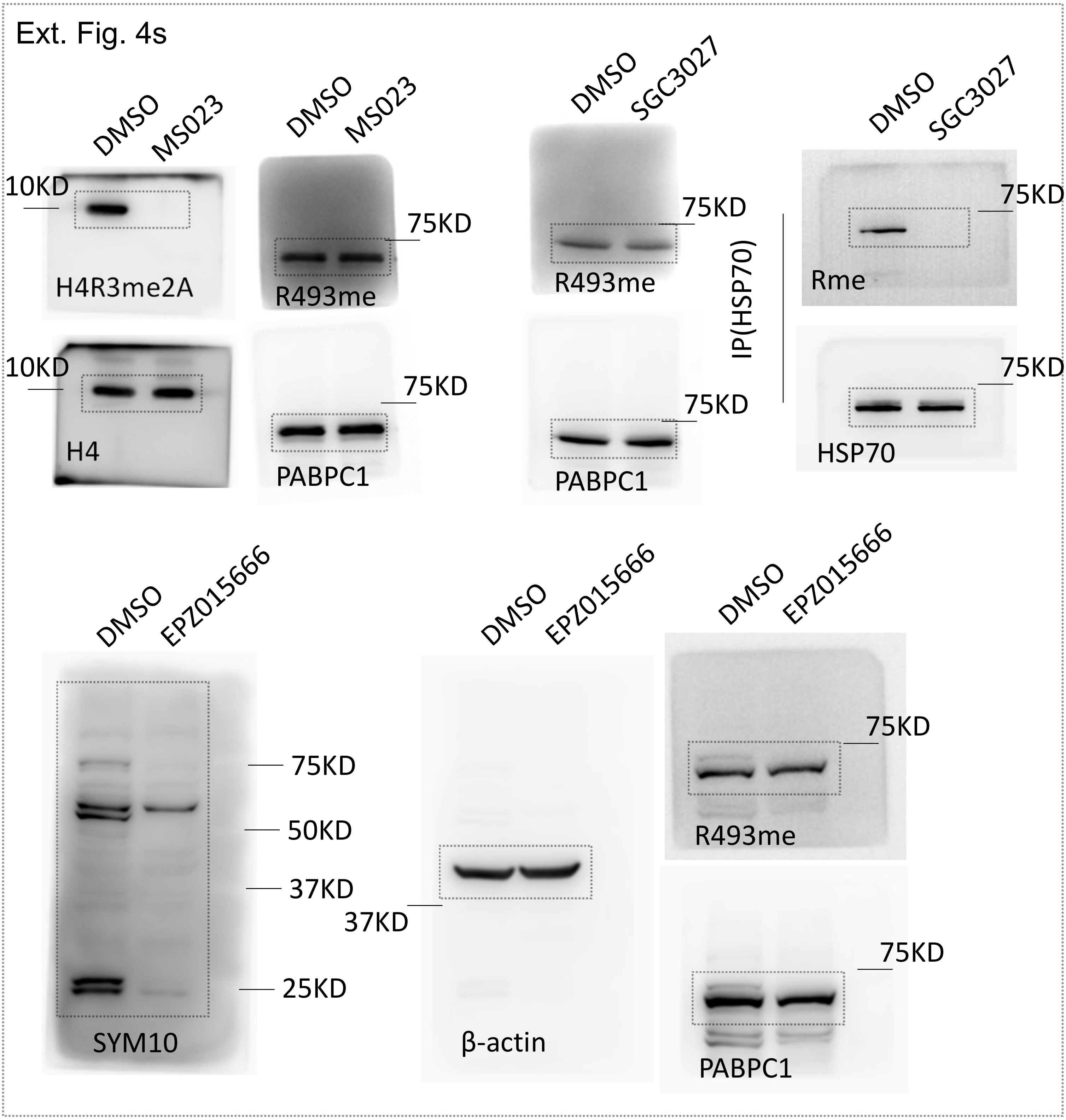

Supplement: Supplementary file 25 — Unprocessed immunoblots. [file 43018_2024_736_MOESM25_ESM.pdf]
