# Supplementary material for: Targeting PRMT9-mediated arginine methylation suppresses cancer stem cell maintenance and elicits cGAS-mediated anticancer immunity
Source: Nat Cancer. 2024 Feb 27;5(4):601–24. doi: 10.1038/s43018-024-00736-x (PMC11056319; doi:10.1038/s43018-024-00736-x)

Extended Data Fig. 8 Unprocessed western blots

Extended Data Fig. 8i

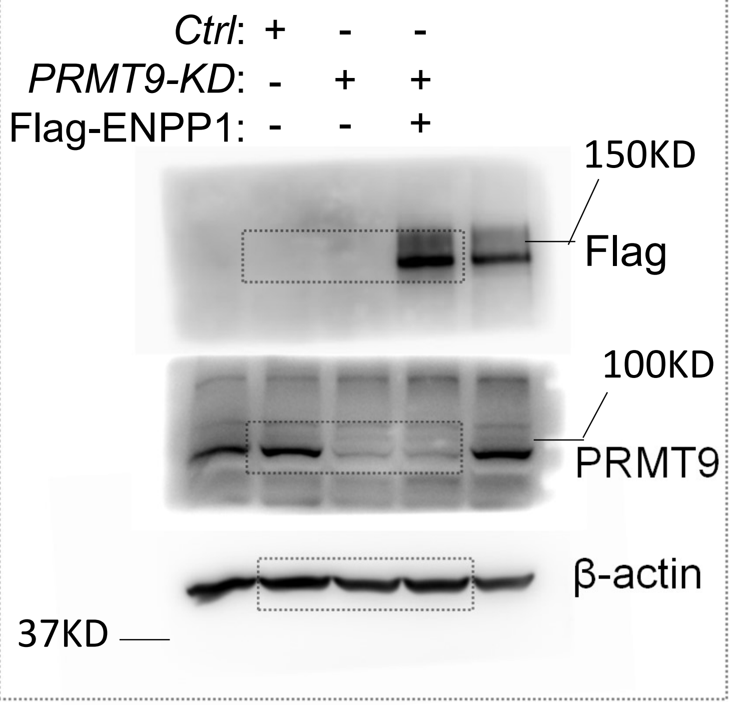

Extended Data Fig. 8n

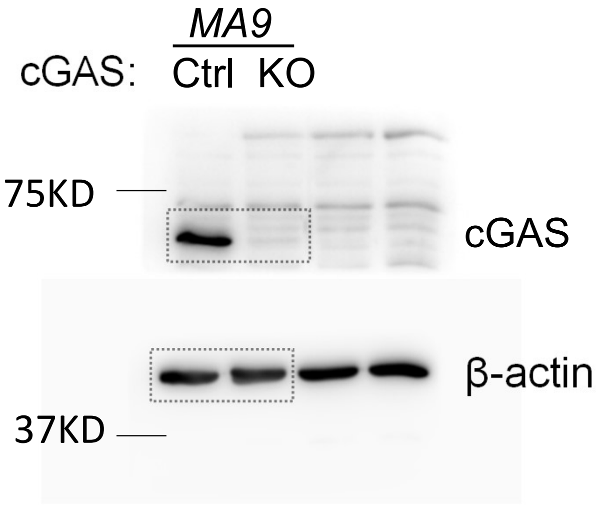

Extended Data Fig. 8j

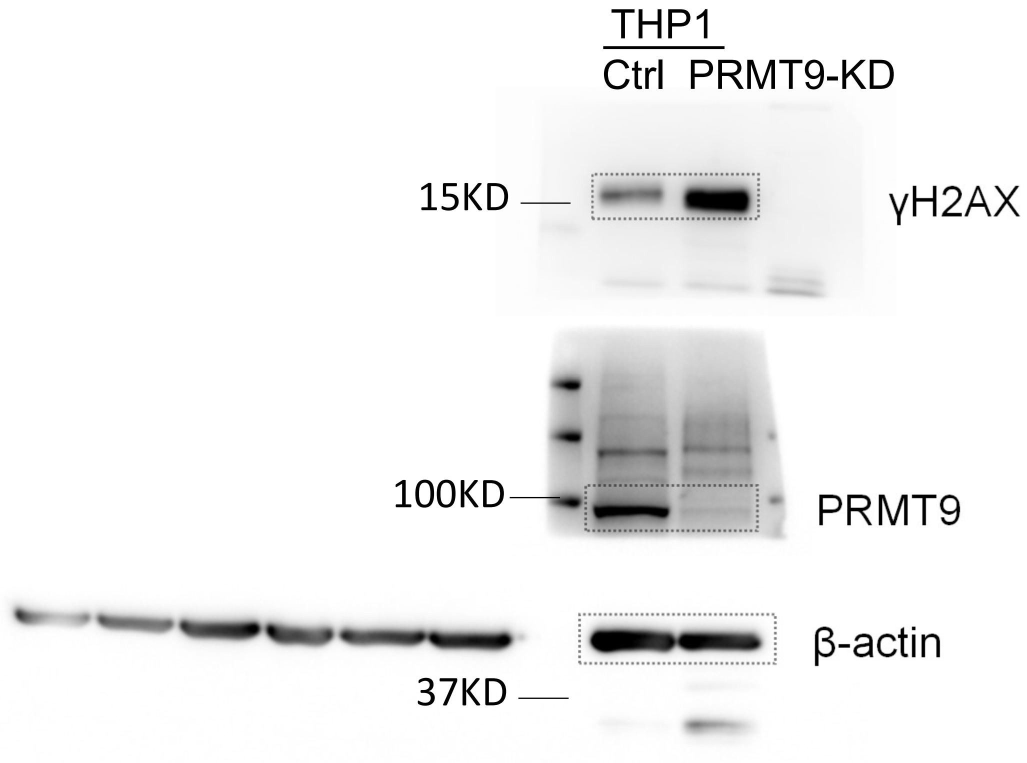

Extended Data Fig. 8 Unprocessed western blots

Extended Data Fig. 8t

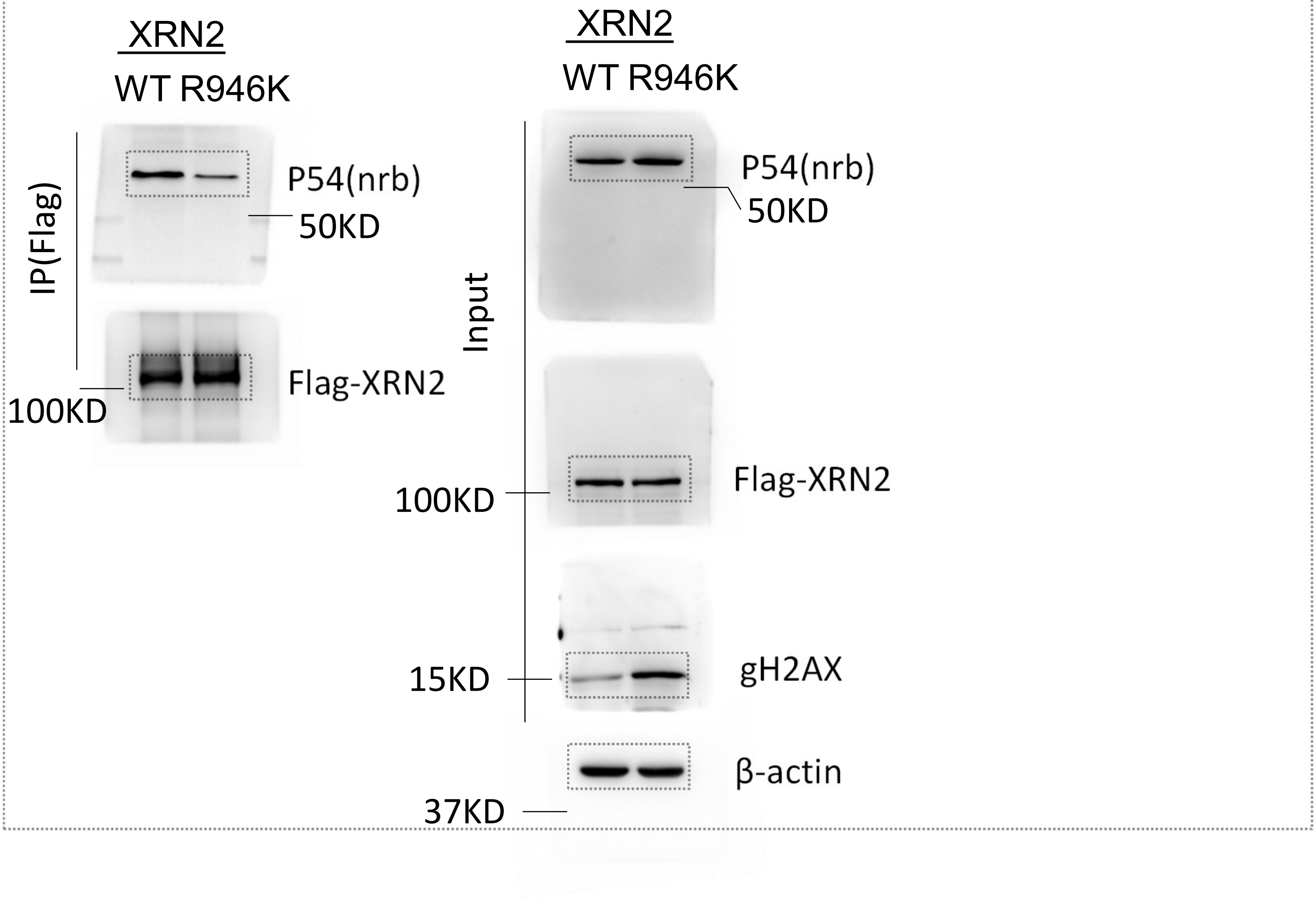

Supplement: Supplementary file 31 — Unprocessed immunoblots. [file 43018_2024_736_MOESM31_ESM.pdf]
